# Effects of Sodium Butyrate Treatment on Histone Modifications and the Expression of Genes Related to Epigenetic Regulatory Mechanisms and Immune Response in European Sea Bass (*Dicentrarchus Labrax*) Fed a Plant-Based Diet

**DOI:** 10.1371/journal.pone.0160332

**Published:** 2016-07-29

**Authors:** Genciana Terova, Noelia Díaz, Simona Rimoldi, Chiara Ceccotti, Emi Gliozheni, Francesc Piferrer

**Affiliations:** 1 Department of Biotechnology and Life Sciences, University of Insubria, Via J.H.Dunant, 3, 21100, Varese, Italy; 2 Institut de Ciències del Mar, Consejo Superior de Investigaciones Científicas (CSIC), Passeig Marítim, 37–49, 08003, Barcelona, Spain; 3 Inter-University Centre for Research in Protein Biotechnologies "The Protein Factory"- Polytechnic University of Milan and University of Insubria, Varese, Italy; Nord University, NORWAY

## Abstract

Bacteria that inhabit the epithelium of the animals’ digestive tract provide the essential biochemical pathways for fermenting otherwise indigestible dietary fibers, leading to the production of short-chain fatty acids (SCFAs). Of the major SCFAs, butyrate has received particular attention due to its numerous positive effects on the health of the intestinal tract and peripheral tissues. The mechanisms of action of this four-carbon chain organic acid are different; many of these are related to its potent regulatory effect on gene expression since butyrate is a histone deacetylase inhibitor that play a predominant role in the epigenetic regulation of gene expression and cell function. In the present work, we investigated in the European sea bass (*Dicentrarchus labrax*) the effects of butyrate used as a feed additive on fish epigenetics as well as its regulatory role in mucosal protection and immune homeostasis through impact on gene expression. Seven target genes related to inflammatory response and reinforcement of the epithelial defense barrier [*tnf*α (tumor necrosis factor alpha) *il1β*, (interleukin 1beta), *il-6*, *il-8*, *il-10*, and *muc2* (mucin 2)] and five target genes related to epigenetic modifications [*dicer1*(double-stranded RNA-specific endoribonuclease), *ehmt2* (euchromatic histone-lysine-N-methyltransferase 2), *pcgf2* (polycomb group ring finger 2), *hdac11* (histone deacetylase-11), and *jarid2a* (jumonji)] were analyzed in fish intestine and liver. We also investigated the effect of dietary butyrate supplementation on histone acetylation, by performing an immunoblotting analysis on liver core histone extracts. Results of the eight-week-long feeding trial showed no significant differences in weight gain or SGR (specific growth rate) of sea bass that received 0.2% sodium butyrate supplementation in the diet in comparison to control fish that received a diet without Na-butyrate. Dietary butyrate led to a twofold increase in the acetylation level of histone H4 at lysine 8, but showed no effect on the histone H3 at Lys9. Moreover, two different isoforms of histone H3 that might correspond to the H3.1 and H3.2 isoforms previously found in terrestrial animals were separated on the immunoblots. The expression of four (*il1 β*, *il8*, *irf1*, and *tnf*α) out of seven analyzed genes related to mucosal protection and inflammatory response was significantly different between the two analyzed tissues but only *il10* showed differences in expression due to the interaction between tissue and butyrate treatment. In addition, butyrate caused significant changes *in vivo* in the expression of genes related to epigenetic regulatory mechanisms such as *hdac11*, *ehmt2*, and *dicer1*. Statistical analysis by two-way ANOVA for these genes showed not only significant differences due to the butyrate treatment, but also due to the interaction between tissue and treatment.

## Introduction

Bacteria associated with the epithelium of an animal’s digestive tract play a critical role in establishing and maintaining their host’s health. The intestinal microbiota is involved in the anaerobic fermentation of complex dietary carbohydrates (cellulose, hemicellulose, pectin), and oligosaccharides that are otherwise indigestible as well as of digestible simple carbohydrates such as starch, and glucose that escape digestion and absorption in the small intestine [1]. Intestinal mucus, sloughed cells from the epithelia, lysed microbial cells, and endogenous secretions provide other sources of fermentable substrates, especially proteins and polysaccharides [1]. Nearly 75% of the energy content of the fermented carbohydrates is used to produce metabolic end products such as short chain fatty acids (SCFAs), which are then readily absorbed by the host, whereas the remaining 25% is used for microbial growth and maintenance or lost as hydrogen, carbon dioxide, and methane [2,3,4,5].

SCFAs, also known as volatile fatty acids, are carboxylic acids with aliphatic tails of 1 to 6 carbon atoms that exist in straight- and branched-chain conformations. Common SCFAs include acetic (C2), propionic (C3), butyric (C4), valeric (C5), and caproic (C6) acid [4]. The predominant anions in either the rumen or large intestine are the short, straight-chain FAs such as acetate, propionate, and butyrate, whereas the short branched-chain FAs, isobutyrate and isovalerate, which are produced by fermentation of the amino acids valine and leucine, respectively, are found in much smaller amounts [1,6].

Among the SCFAs, butyrate has received particular attention due to its numerous positive effects on the health of intestinal tract and peripheral tissues [7]. In addition to being the main respiratory fuel source of the colonic bacteria, and preferred to glucose or glutamine, butyrate plays a major role in enhancing epithelial cell proliferation and differentiation and in improving the intestinal absorptive function [8,9,4]. Furthermore, there are several lines of evidence suggesting that butyrate has potential immunomodulatory and anti-inflammatory properties in the intestine and may prevent colorectal cancer in humans [10, 11, 12].

Although the exact underlying mechanisms of action have not yet been elucidated, the influence of butyrate on cell proliferation may be explained, at least in part, by its potent regulatory effect on gene expression. This effect is often attributed to the ability of butyrate to inhibit the activity of many histone deacetylases, leading to hyperacetylation of histones [12]. Histone acetylation modifies chromatin structure, allowing the binding of transcription factors and polymerases and hence, the beginning of transcription. The modulation of gene expression through core histone acetylation is one of the most relevant means by which cell function and DNA methylation are epigenetically regulated [12,13,14]. A positive effect of butyrate on transcriptomic activity of some pivotal genes at the intestinal level has also been suggested in fish in two recent studies carried out on European sea bass (*Dicentrarchus labrax*) [15] and gilthead sea bream (*Sparus aurata*) [16].

Much of the research on butyrate has focused on its role in the gut, while less is known about whole-body metabolism of butyrate and, in particular, on how it might influence the metabolic potential of the liver *in vivo* [17, 18]. Although butyrate is largely taken up by the intestinal epithelium, a small fraction can also reach the liver through the blood stream via the portal vein [18, 19]. In liver, butyrate is readily converted in mitochondria to butyryl CoA to produce ketone bodies (rather unlikely in fed animals) and acetyl CoA, which then enters into the Krebs cycle [19, 20, 21]. Hepatic metabolism and clearance of butyrate are substantial since evidence shows that close to 100% was removed in the liver of rodents fed with a high-fiber diet [22], whereas butyrate released from the human gut *in vivo* into the circulatory system was counterbalanced by hepatic butyrate uptake [18], indicating that the liver is highly involved in butyrate metabolism [23,24].

For butyrate to exert its physiologic, cellular, and molecular effects, circulating concentrations would need to be maintained at a consistently high level. This is difficult to attain because plasma clearance of butyrate is very quick, with a half-life of about 6 min when given intravenously in humans [25]. A possible solution to circumvent problems associated with rapid metabolism of butyrate would be to administer it orally by giving multiple daily doses of stable derivatives of butyrate. Indeed, when stable derivatives of butyrate were given orally as opposed to intravenously in humans, its half-life was increased to 40 min, and circulating butyrate concentrations reached high enough values to be efficacious [25]. In farmed animals such as pigs and chickens, butyrate included in the diet has had a positive influence on body weight gain, feed utilization, and composition of intestinal microflora, as well as trophic effects on the intestinal epithelium through an increase in the villi length and crypt depth [26,27,28]. In poultry, butyrate applied as a nutritional supplement caused *in vivo* hyperacetylation of the hepatic core histones and modified the epigenetic regulation of hepatocyte’s function [7]. In addition, some authors have suggested significant improvements in fish growth and feed conversion rates when butyrate is included in diets of some species such as catfish [29], tilapia, carp [30], and sea bream [16], but not in others such as salmon [31,32]. However, except for these studies, literature concerning the use of butyrate or its derivatives as an additive in fish feed is very scarce.

Accordingly, the present study aimed to evaluate in the European sea bass (*Dicentrarchus labrax*) the potential effects of butyrate as a feed additive on fish growth, as well as butyrate’s regulatory role on the mucosal protection and immune homeostasis through its effects on gene expression. The target genes related to mucosal inflammatory response and reinforcement of the mucous defense barrier included: *tnf*αtumor necrosis factor alpha), which is a cell-signaling protein (cytokine) that makes up the inflammatory acute phase reaction and possesses a wide range of proinflammatory actions [33]; interleukins such as *il1β*, *il-6*, *il-8*, and *il-10*, which are well-known cytokines that regulate immune responses, inflammatory reactions, and hematopoiesis; *irf1* (interferon regulatory factor 1), which is a transcription factor that stimulates both innate and acquired immune responses by activating specific target genes expressed during inflammation, immune responses, and hematopoiesis [34]; and *muc2* (mucin 2), which is a major component of intestinal mucus gel secretions that serve as a barrier to protect the intestinal epithelium [35].

The second goal of the present study was to evaluate the epigenetic effects of dietary butyrate in sea bass by monitoring both the acetylation state of hepatic core histones and the hepatic and intestinal expression of a suite of genes related to epigenetic modifications [36]. These genes included: *dicer 1*, which encodes an active, small RNA component that represses the expression of other genes [37]; *ehmt2* (euchromatic histone-lysine-N-methyltransferase 2), which demethylates Lys9 in histone 3 in euchromatin, creating a tag for epigenetic transcription repression [38,39]; *pcgf2* (polycomb group ring finger 2), which acts via chromatin remodeling and histone modification [40]; *hdac11* (histone deacetylase-11), which can modify core histone octamer packing chromatin in dense structures or controls various histone methyltransferase complexes [41]; and *jarid2a* (jumonji), which is a nuclear factor that functions as a powerful transcriptional repressor [42].

## Materials and Methods

### Ethics Statement

This study was carried out in strict accordance with the recommendations in the Guide for the Care and Use of Laboratory Animals of the University of Insubria, Varese, Italy. The Committee on the Ethics of Animal Experiments of the same University approved all of the protocols performed. Fish handling was performed under tricaine methanesulfonate (MS222) anesthesia, and all efforts were made to minimize discomfort, and stress and to avoid pain to the animals.

### Fish and Experimental Set Up

Juvenile European sea bass (*Dicentrarchus labrax*) were purchased from a commercial hatchery (Civitavechia, Italy). Upon arrival to the laboratory, fish were stocked for 40 days into two rectangular indoor tanks of 2.5 cubic meters to acclimate.

At the beginning of the trial, after removing fish deviating from the average weight of approximately 15 g, we distributed fish into six circular experimental tanks (3 replicates) of 600 L each, at a density of 35 fish per tank and let them to acclimate over a period of one week. There were no significant differences in fish weight between the experimental tanks at the onset of the experiment (*P*>0.05; data not shown).

### Rearing Facility and Maintenance

All rearing tanks were located in an indoor facility. The tanks were equipped with re-circulating systems and photoperiod, temperature, and salinity could be strictly controlled with this equipment. The experimental layout consisted of six cylindrical 600 L fiberglass tanks, connected to a central main biofilter of 350 liters. The light source was the natural photoperiod enhanced with florescent light, providing a light intensity of 1200 lx during the day. The water was heated and maintained at 21 ± 1°C by using submersible aquarium heaters. The salinity was 22 ± 0.5 g/l throughout the experiment.

Twice a week the following parameters were measured: dissolved oxygen, pH, ammonia, and nitrite levels. The levels of all parameters remained within the range considered optimal for European sea bass growth throughout the experiment.

### Diet Formulation, and Feeding

As a control diet we used a formulation of 40% crude protein and 16% fat, which was based on plant protein and fishmeal. The control diet was similar to feed commercially available for growing European seabass. Control diet was supplemented with 2g/kg (0.2%) of sodium butyrate to produce the experimental butyrate diet. A detailed diet composition is presented in Table 1. Diets were prepared using small-scale machinery for mixing ingredients and preparing pellets of 3.5 mm in diameter. Na-butyrate substituted an equivalent amount of filler in the butyrate diet.

**Table 1:** Composition of the diets in g/100 g on a dry weight basis.

Ingredients (g/100g)	Control	Butyrate
Fish meal	10.00	10.00
Soybean meal	30.00	30.00
Pea concentrate	16.00	16.00
Corn gluten	14.20	14.20
Wheat gluten	5.00	5.00
Fish oil	14.00	14.00
Stay-C 35d	0.03	0.03
Vitamin Mix	0.40	0.40
Mineral Mix	1.00	1.00
DL-Methionine	0.25	0.25
Lysine (98%)	0.05	0.05
Fish Hydrolysate	2.00	2.00
Dextrin	1.56	1.56
Sodium alginate	0.79	0.79
Dicalcium phosphate	0.72	0.72
Filler (gelatin)	4.00	3.80
Na-butyrate	-	0.20
Total	100.00	100.00

Each diet was provided to fish in triplicate (3 tanks/diet). Fish were fed twice a day and feeding rates were restricted to 3.0% of biomass. The feeding experiment was based on four-weekly fish weight measurements to adjust the feed ration to a similar percentage of fish biomass in both treatments. Feed consumption (g) in each tank was estimated from the difference between feed delivered into the tank and uneaten feed, which was collected from the bottom of the tank. The feeding trial lasted 8 weeks. Fish SGR was calculated using the following formula: (ln Wf—ln Wi)/t x 100, where Wf is the final weight (g), Wi is the initial weight (g), and t is growth time (days).

### Fish Sampling

At the end of the eight-week-long feeding trial, fish of each tank were individually weighed after overnight food deprivation. Six fish from each treatment (three fish/tank) were then randomly fished, and sacrificed. Intestine and liver were excised from each sampled fish using sterile instruments, snap-frozen in dry ice, and then kept at minus 80°C until nucleic acid extraction and histone protein acetylation analysis.

#### Growth data statistical analysis

Growth data were analyzed by two-way analysis of variance (two-way ANOVA) considering diet, time and their interaction as sources of variation, followed by Tukey's HSD post hoc test. Significance level was set at *P*< 0.05.

### Preparation of Liver Nuclear Protein Fraction

Liver nuclear protein extracts were prepared from six fish per group using 3 ml/g of tissue of extraction buffer containing: 10 mM Tris/HCl, pH 7.8, 10 mM KCl, 1.5 mM MgCl2, 0.5 mM Pefabloc® (SIGMA-ALDRICH®), 0.5 mM DTT, 1 mM Na3VO4, and 1X protease inhibitor cocktail (SIGMA-ALDRICH®). Tissue lysis and homogenization were carried out in a closed system using the gentleMACS™ Dissociator and single-use gentleMACS™ M tubes (Miltenyi Biotec). Liver lysates were then centrifuged at 1500 *g* for 20 min at 4°C. The supernatants containing the cytosolic protein fraction were discarded while the nuclear pellets were stored at minus 80°C until further histone isolation procedure.

### Histone Isolation

Purified histone extracts were isolated from nuclear fractions using the Histone Purification Mini Kit (Active Motif, Carlsbad, CA, USA) according to the manufacturer’s protocol. Active Motif’s Histone Purification Kit preserves phosphoryl, acetyl, and methyl post-translational modifications on histones. Briefly, an equal volume of ice-cold extraction buffer was added to the nuclear suspension. After homogenization, samples were left overnight in the extraction buffer on a rotating platform at 4°C. Next day, tubes were centrifuged at maximum speed for 5 min in a microfuge at 4°C and the supernatants, which contained the crude histone extracts, were neutralized with one-fourth volume of 5x neutralization buffer (pH 8.0). Neutralized extracts were loaded to previously equilibrated histone isolation spin columns. After three washes with histone wash buffer, we eluted histones in 100 μl of histone elution buffer and precipitated overnight by adding 4% perchloric acid. On the following day, samples were centrifuged at maximum speed for 1 hour; histone pellets were washed first with 4% perchloric acid, later with acetone containing 0.2% HCl, and finally with pure acetone, after which they were air dried. Histones were suspended in sterile distilled water and the yield of total core histone proteins was quantified by measuring the absorbance at 230 nm.

### Histone Acetylation Western Blots

Western blotting analyses were performed on four samples of purified histones, given that the quantity of histones isolated from the other two nuclear protein extracts resulted not sufficient. For the analysis, we followed the instructions of the Acetyl Histone Antibody Sampler Kit (Cell Signaling) and the protocol applied by Mátis *et al*. [7]. Before using “Acetyl-Histone H4 (Lys8) Antibody #2594”and Histone H4 (L64C1) #2935 provided with the kit, we used ClustalW to perform a multiple sequence alignment between the human histone H4 peptide sequence that was used for the production of antibodies and the ortholog sequences in European seabass (*Dicentrarchus labrax*), and other teleosts such as zebrafish (*Danio rerio*), Nile tilapia (*Oreochromis niloticus*), and Atlantic salmon (*Salmo salar*). As shown in S1 Fig, the histone H4 peptide sequence in European sea bass presents 100% similarity with the human sequence, and it is the same for the other teleosts’ histone H4 sequences. This suggest that antibodies were suitable for the detection of the antigen in our target species.

Histone proteins were diluted by 2x SDS and β -mercaptoethanol containing loading buffer (supplemented with 50 mM DTT), sonicated for 15 s, and heat denatured at 95°C for 5 min. Histones were separated by SDS-PAGE on polyacrylamide (4–20%) precast gradient gels (Bio-Rad); 3 μg histone protein per lane were loaded for the detection of histones H2A, H2B, and H3, whereas 6 μg per lane were loaded for histone H4. After electrophoresis, proteins were blotted onto PVDF membranes (0.22-μm pore size, Bio-Rad). Before proceeding to the immunodetection process, a reversible Ponceau staining was applied to membranes to test equal loading of gels and protein transfer. Histones were identified using antibodies furnished by the Acetyl Histone Antibody Sampler Kit. After blocking with 5% fat-free milk containing PBST for 3 h, the immunoblots were incubated overnight at 4°C with primary antibodies against histone H2A (1:1000), H2B (1:500), H3 (1:1000), H4 (1:500), and their acetylated forms. Each acetyl histone antibody was specific for the target histone modified at the lysine residue of the most frequent acetylation site (AcH2A and AcH2B: Lys 5, AcH3: Lys 9, AcH4: Lys 8). The primary antibody was detected using an anti-rabbit secondary antibody (1:2000) or an anti-mouse secondary antibody (1:900) for the non-acetylated H4 histone. Both secondary antibodies were coupled with horseradish peroxidase. Primary antibodies were diluted in PBST containing 5% BSA, with the exception of anti H4, which was diluted in PBST containing 5% of defatted milk. Secondary antibodies were diluted in PBST containing 5% fat-free milk. Signals were detected using an enhanced chemiluminescence system (SuperSignal®west Dura Extended Duration Substrate, Thermo Scientific) and exposing to clear-blue X-ray film. After film exposure, densitometry was used to quantify protein levels on the western blots by means of Quantity One 1-D software (Bio-Rad). The protein levels were expressed as adjusted volume, Adj. Vol. [OD*mm^2^] = [{Sum of the intensities of the pixels inside the volume boundary} x {area of a single pixel in mm^2^}]–{the background volume}).

### RNA Extraction and cDNA Synthesis for Gene Expression Analysis

RNA from 12 sea bass livers and 12 intestines was extracted using a semi-automatic system (Maxwell^®^ 16 Instrument, Promega) and a total RNA purification kit (Maxwell^®^ 16 Tissue LEV). RNA purity and concentration were assessed by a ND-2000 spectrophotometer (NanoDrop product, Thermo Scientific).

One hundred nanograms of the total extracted RNA were reverse transcribed to cDNA using SuperScript III and random hexamers (Life Technologies, Italy) following the manufacturer’s instructions. Two rounds of cDNA synthesis per sample were carried out and then merged.

### Quantitative Real-Time PCR (qRT-PCR)

For the already cloned genes in European sea bass, FASTA sequences were taken from the NCBI repository (http://www.ncbi.nlm.nih.gov/) and primers were designed by using Primer3 Plus (http://www.bioinformatics.nl/cgi-bin/primer3plus.cgi). For the genes not cloned yet, exon sequences from other fish species (stickleback or tilapia) were taken from the Ensembl Genome Browser (http://www.ensembl.org/) and blasted against the European sea bass genome database (http://seabass.mpipz.de/) [43]. Only when the match was annotated in the sea bass genome the exon was considered for primer design (S1 Table and S2 Fig). Primer efficiency was evaluated by analyzing the slope of a linear regression from six different dilutions using a pool with all the samples involved in the analysis: six fish per treatment in the two different tissues. Efficiencies ranged from 1.8 to 2.4. In addition, the correct binding of the primers and hence the presence of a single amplicon generation was assessed by adding a melting-curve analysis (95°C for 15 s, 60°C for 15 s and 95°C for 15 s) after the amplification phase.

qRT-PCR was performed on an ABI 7900HT (Life Technologies) under a standard cycling program (UDG decontamination cycle: 50°C for 2 min; initial activation step: 95°C for 10 min; 40 cycles of 15 s denaturation at 95°C and 1 min annealing/extension at 60°C). A final dissociation step was also added (95°C for 15 s and 60°C for 15 s).

For qRT-PCR gene analysis, cDNA was diluted 1:10 for all the target genes except for the reference gene, *r18S*, which was diluted 1:500. All samples were run in triplicate in a 384-well plate in a final volume of 10 μl. Each well contained a mix of 5 μl SYBR Green Supermix (Life Technologies), 2 μl distilled water, 2 μl primer mix (forward and reverse at 10 μM concentration), and 1 μl cDNA. Negative controls were added in duplicate. The software SDS 2.3 and RQ Manager (Life Technologies) were used to collect data and calculate gene expression levels (cycle thresholds, Cts), respectively. The expression of housekeeping gene *r18S* (the endogenous control) was used to correct for intra- and inter-assay variations.

### Data Analysis

#### qRT-PCR Raw Data Analysis

Ct values were adjusted, taking into account primer efficiencies per each gene when calculating 2^ddCt values. Expression data for each target gene were also normalized to the housekeeping gene (*r18S*) and fold-change calculations were made based on the Schmittgen and Livak’s method [44].

#### qRT-PCR Statistical Analysis

qRT-PCR analyses were performed using 2^ddCt values in the IBM SPSS Statistics 19 software. Data were evaluated for normality and homoscedasticity of variance; outliers (no more than one per condition) were eliminated when needed. Treated versus control groups, in liver and intestine, were analyzed in two steps: 1) by analyzing fold-change differences with respect to the controls [44] and 2) by a Student *t*-test analysis. In addition, a two-way analysis of variance (ANOVA) was carried out, taking into consideration both treatment and tissue for analyzing not only the contributions of each variable but also their interactions.

## Results

### Effect of Butyrate on Growth Performance

**Fig 1:**
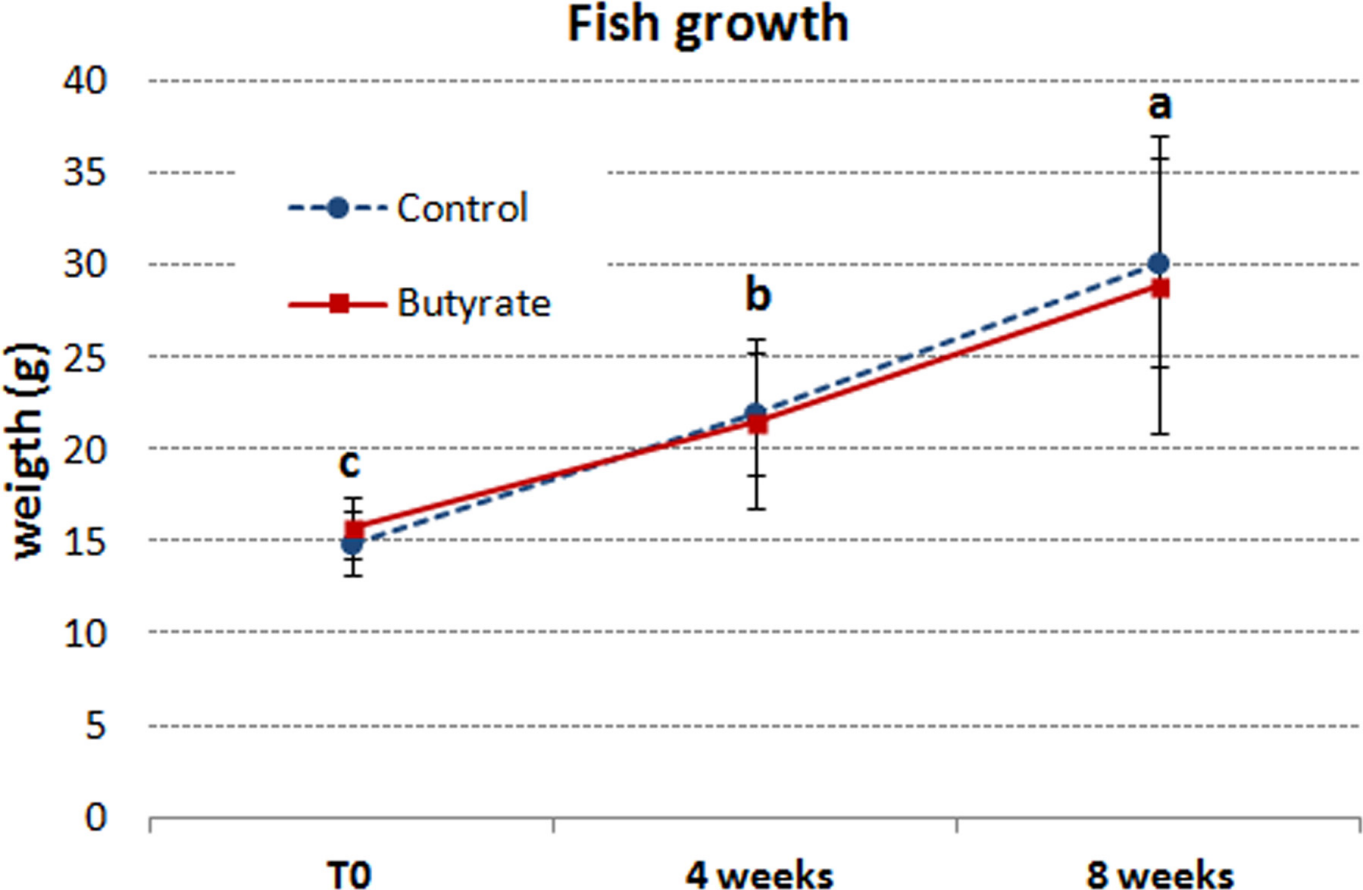
Effects of dietary butyrate on European sea bass growth. The data were tested by ANOVA followed by Tukey's HSD test to determine whether there were any significant differences between different groups. Fish were fed for 8 weeks two different diets, a control diet, and an experimental diet, which was the control diet supplemented with 2g/kg (0.2%) of Na- butyrate. Each histogram shows the mean ± SEM of 105 animals. Different letters indicate significant differences (*P* < 0.05).

The initial weight of 14.91±1.73 g of the control fish group (Fig 1) increased to 20.63±4.17 g after 4 weeks of feeding and to 30.22±5.61 g after 8 weeks of feeding. Fish receiving the butyrate-supplemented diet had an initial mean body weight of 15.80±1.60 g, which increased to 20.51±4.74 g after 4 weeks and to 28.97±8.09 g after 8 weeks of feeding. However, the results of two-way ANOVA showed that, starting from the 4^th^ week of the feeding trial, there was only a time effect on the fish growth, whereas the interaction effect between diet and time was not significant. By considering the main effect of time (S2 Table), the average weight of fish fed butyrate was not significantly different from that of the control fish from the 4^th^ week until the end of the feeding trial.

Survival was high (around 95%) with no significant differences between the groups of fish fed different diets. The SGR of fish fed the butyrate-supplemented diet was 1.06±0.02 after 4 weeks of feeding and 1.19±0.03 at the end of the experiment, whereas that of the control group was 1.34±0.04 and 1.33±0.07 after 4 and 8 weeks of feeding, respectively. There were no significant differences in SGR between the fish fed control and butyrate diet (data not shown).

### Effect of Butyrate on Core Histone Acetylation

**Fig 2:**
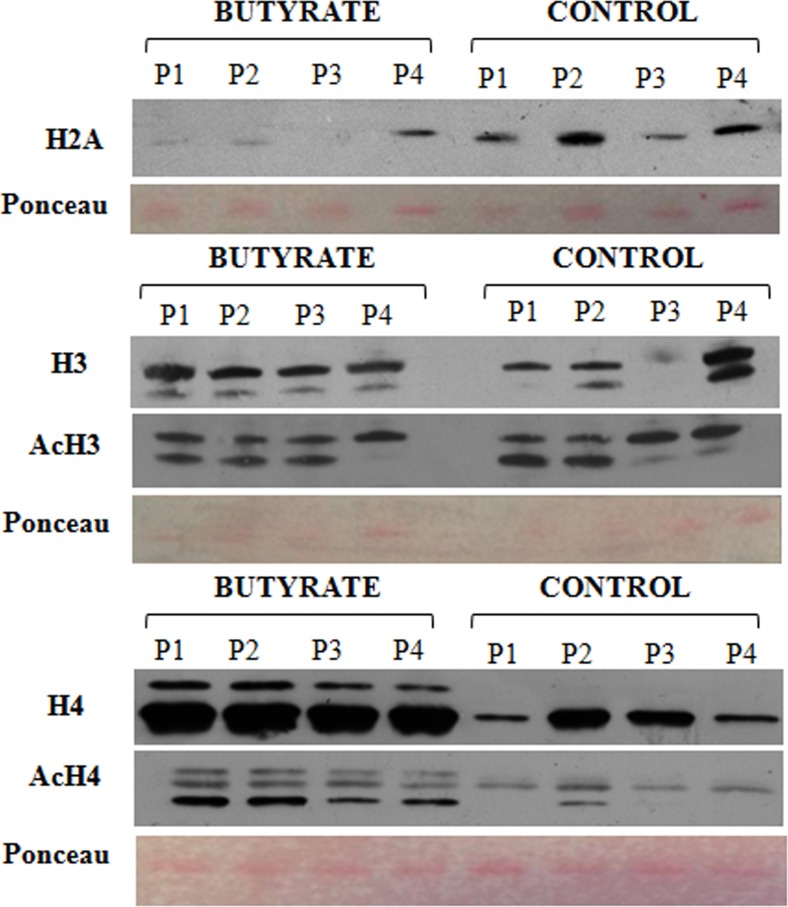
Effects of butyrate on the acetylation state of histones from isolated hepatocytes in European sea bass. One–dimensional immune-blotting analysis of histones H2A and H3 as well as H3, H4 acetylated histones is shown. Each column represents individual fish. 3 μg histone protein per lane were loaded for the detection of histones H2A, and H3, and 6 μg per lane for histone H4. Before immunodetection, a reversible Ponceau staining was applied to membranes to test equal loading of gels and protein transfer. After X-ray film exposure, densitometry was used to quantify protein levels on the western blots by means of Quantity One 1-D software (Bio-Rad). Putative isoforms for histone H3 [H3.1 (upper band) and H3.2 (lower band)] were accounted for the densitometry analysis.

To investigate the effect of dietary supplementation of sodium butyrate on histone acetylation in European sea bass, we performed an immunoblotting analysis on liver core histone extracts of four fish from each group. The results of this analysis are presented in Fig 2, whereas the intensity values (Adj. Vol **[**OD*mm2]) of each band are reported in Table 2. Among the primary antibodies furnished by the Acetyl-Histone Antibody Sampler Kit (Cell Signaling Technology) anti-H2A (non-acetylated form), anti-H3 (acetylated and non-acetylated forms), and anti-H4 (acetylated and non-acetylated forms) recognized respective sea bass’s epitopes. In contrast, none of the anti-H2B, anti-AcH2B, and anti Ac-H2A antibodies recognized any of the sea bass epitopes.

**Table 2:** Quantification of core histone protein expression (Adj. Vol [OD*mm2]) and H4 acetylation ratio by densitometry. (*) (**) indicate statistical significant differences between experimental groups with *P* <0.05 and *P* <0.01, respectively.

	BUTYRATE	CONTROL	
Histone	Adj. Vol [OD*mm^2^]		*t*-test
**H2A**	1.12 ± 1.10*	5.46 ± 2.62	*P* < 0.05
**H3**	5,43 ± 1.36	5.78 ± 4.64	
**AcH3**	6.42 ± 1.33	8.36 ± 1.20	
**H4**	38.23 ± 6.48**	10.18 ± 7.81	*P* < 0.01
**AcH4**	6.55 ± 3.30**	0.53 ± 0.44	*P <* 0.01
	**Acetylation ratio**		
**AcH4/H4**	0.16 ± 0.05*	0.07 ± 0.04	*P* < 0.05

Immunoblotting on hepatocyte core histone extracts (Table 2) revealed that dietary butyrate intake decreased the relative protein expression level of the H2A histone (*P*<0.05), which was poorly expressed in butyrate-treated fish but was detected at high amounts (fivefold more) in control fish. Screening of the principal acetylation sites of core histones revealed that butyrate treatment caused hyperacetylation of histone H4. Indeed, the addition of sodium butyrate to the diet significantly increased the ratio of AcH4/H4 at lysine 8 (*P*<0.05), leading to an approximately twofold increase in comparison to the control group (no butyrate) (Table 2). In contrast, the acetylation state of histone H3 at Lysine 9 was not significantly influenced by butyrate dietary intake. Interestingly, two different isoforms of histone H3 were separated on in the immunoblots, which could correspond to the H3.1 and H3.2 isoforms previously found in chicken [7].

### Genes Related to Epigenetic Regulatory Mechanisms

**Fig 3:**
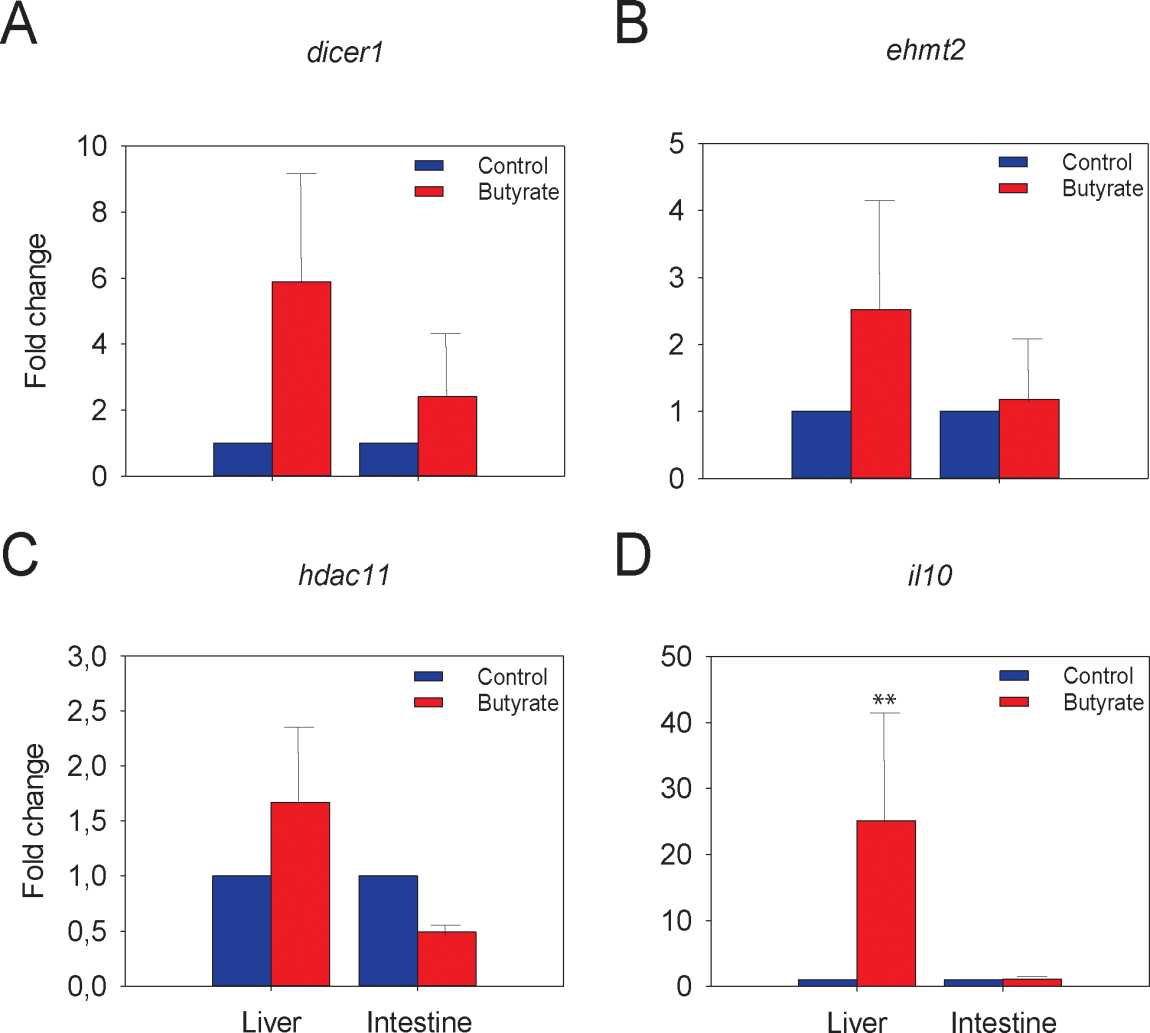
Effects of dietary butyrate on gene expression in two tissues of the European sea bass: liver and intestine, as determined by qRT-PCR analysis. Only those genes that showed statistical differences for the interaction between tissue and treatment (A: *dicer1*, B: *ehmt2* and C: *hdac11*), or differences in expression solely due to the treatment (D: *il10*) are depicted. Fish were fed for 8 weeks two different diets, a control diet, similar to feed commercially available for growing European seabass, and the experimental diet, which was the control diet supplemented with 2 g/kg (0.2%) of Na-butyrate. The means of six animals in each group are shown.

Regardless of treatment, a two-way ANOVA showed that the differences between hepatic and intestinal levels of expression of five target genes related to epigenetic regulatory mechanisms were statistically significant (*P*<0.05) or highly significant (*P*<0.01; *P*<0.001) (Table 3 and S3 Table), being in general higher in the intestine. However, pairwise individual comparisons between control and treated fish for each tissue and gene analyzed by a Student’s t-test showed no differences in any case, despite fold-change ranges of 0.49 to 2.66 in the intestine and of 1.67 to 14.74 in the liver. This could be due to the high variability observed between fish. Furthermore, regardless of tissue, *ehmt2* showed significant differences due to butyrate treatment (*P* = 0.002), with significant differences (*P* = 0.010) for the interaction between tissue and treatment, too. Similarly, *dicer1* and *hdac11* showed statistically significant differences due to the interaction between tissue and treatment (*P* = 0.050 and *P* = 0.038, respectively). Fold-change differences in the expression of genes that reached significance due to tissue, treatment, or both are shown in Fig 3A–3C.

**Table 3:** Two-way ANOVA statistical analysis of the expression of genes involved in epigenetic regulatory mechanisms.

Gene	2- way ANOVA	
*dicer 1*	F (Ts)	14.661 (*P* = 0.001)***
	F (Tr)	0.025 (*P* = 0.875)
	F (Ts x Tr)	2.219 (*P* = 0.050)*
*ehmt2*	F (Ts)	61.878 (*P* = 0.000)***
	F (Tr)	13.426 (*P* = 0.002)**
	F (Ts x Tr)	8.093 (*P* = 0.010)**
*pcgf2*	F (Ts)	7.211 (*P* = 0.014)*
	F (Tr)	0.003 (*P* = 0.096)
	F (Ts x Tr)	0.024 (*P* = 0.878)
*jarid2a*	F (Ts)	6.159 (*P* = 0.022)*
	F (Tr)	0.825 (*P* = 0.374)
	F (Ts x Tr)	0.385 (*P* = 0.542)
*hdac11*	F (Ts)	45.051 (*P* = 0.000)***
	F (Tr)	0.002 (*P* = 0.969)
	F (Ts x Tr)	4.843 (*P* = 0.038)*

*Note*: Asterisks mark statistical differences (*P<0.05; ** *P*<0.01; *** *P*<0.001). Ts = Tissue, Tr = Treatment, Ts x Tr denotes de interaction between Tissue and Treatment.

### Genes Related to Mucosal Protection and Inflammatory Response

Statistical analysis by two-way ANOVA revealed that the expression of four (*il1 β*, *il8*, *irf1*, and *tnf*α) out of seven target genes related to inflammatory response and immune system was significantly different (*P*<0.05) between the two analyzed tissues (liver and intestine) but only the *il10* gene showed differences in expression (*P* = 0.003) due to the butyrate treatment (Table 4 and S4 Table). This effect was also demonstrated with pairwise comparisons using Student’s *t*-test (*P* = 0.002). In contrast to what was observed with the epigenetic regulatory mechanism-related genes and with the exception of *il10* in the liver (fold change 25.09±17.18; Fig 3D), the magnitude of fold change in the other two genes (*il6*, *muc2*) was lower (range 0.01–4.74). Furthermore, in contrast to the epigenetic regulatory mechanism-related genes, the interaction effect between tissue and treatment did not reach statistical significance for any of the seven target genes related to the inflammatory response and mucosal protection.

**Table 4:** Two-way ANOVA statistical analysis of the expression of genes involved in inflammatory response, mucosal protection, and immune homeostasis.

Gene	2- way ANOVA	
*il1 β*	F (Ts)	11.368 (*P* = 0.003)**
	F (Tr)	0.000 (*P* = 1.000)
	F (Ts x Tr)	0.000 (*P* = 1.000)
*il6*	F (Ts)	2.068 (*P* = 0.165)
	F (Tr)	1.126 (*P* = 0.301)
	F (Ts x Tr)	0.949 (*P* = 0.341)
*il8*	F (Ts)	8.129 (*P* = 0.009)**
	F (Tr)	0.632 (*P* = 0.435)
	F (Ts x Tr)	0.660 (*P* = 0.425)
*il10*	F (Ts)	0.036 (*P* = 0.851)
	F (Tr)	10.881 (*P* = 0.003)**
	F (Ts x Tr)	1.007 (*P* = 0.326)
*irf1*	F (Ts)	48.930 (*P* = 0.000)***
	F (Tr)	2.401 (*P* = 0.136)
	F (Ts x Tr)	1.505 (*P* = 0.233)
*tnf*α	F (Ts)	55.649 (*P* = 0.000)***
	F (Tr)	0.000 (*P* = 1.000)
	F (Ts x Tr)	0.000 (*P* = 1.000)
*muc2*	F (Ts)	4.241 (*P* = 0.059)
	F (Tr)	0.148 (*P* = 0.706)
	F (Ts x Tr)	0.070 (*P* = 0.795)

*Note*: Asterisks mark statistical differences (** *P*<0.01; *** *P*<0.001). Ts = Tissue, Tr = Treatment, Ts x Tr denotes interaction between Tissue and Treatment.

## Discussion

Due to the paucity of oceanic resources utilized in the preparation of diets for cultured fish, the amount of fishmeal (FM) included in compound aquafeeds is steadily decreasing and commercial feed producers have been trying to replace FM by using alternative protein sources such as vegetable proteins meals (VMs) [45]. VMs are able to replace a substantial part of the FM. However, these products have limitations due to unbalanced amino acid profiles, high fiber content, antinutritional factors and competition with use for human consumption [46]. Therefore, to further proceed with low FM inclusion levels, fish feeds should be adequately supplemented with natural feed additives such as butyrate [47] or other organic acids, which have generated increasing interest in the industry. Currently, there is strong interest in the use of organic acids and their salts as natural feed additives since such products seem to have growth-promoting effects in livestock. Their positive effects are well documented in terrestrial livestock production [28,48,49,50], but some questions remain regarding their efficacy in fish farming, and conflicting reports exist on the subject. Indeed, growth was significantly enhanced in some fish species, such as rainbow trout (*Oncorhynchus mykiss*), when fed an organic acid blend supplement mainly consisting of formate and sorbate [51], but not in trout fed other commercial supplements such as lactic acid [52] or citric acid [52,53]. On the other hand, neither hybrid tilapia (*Oreochromis niloticus* x *O*. *aureus*) fed potassium diformate [54] nor Atlantic salmon (*S*. *salar*) fed sodium salts of acetic, propionic, and butyric acid (5:5:2 w/w/w) showed any growth enhancement [55, 31]. Species differences may thus occur. The results of our work are in accordance with the last two studies, Gislason et al., [55], and Bjerkeng et al., [31] as we did not find differences in the growth of European sea bass fed a diet supplemented with Na-butyrate. To date, literature related to the use of butyric acid or its salts in fish feed is still scarce and mainly focused on the effects of butyrate on fish growth performance, intestinal morphology, and metabolism [55,32,16,56]. Only few reports have described butyrate-induced epigenetic and transcriptional changes in intestinal and hepatic genes of farmed fish [15,56]. In view of this, the present study aims to contribute to the current understanding of the epigenetic regulatory effects of butyrate in European sea bass, which is one of the most important species in Mediterranean aquaculture.

Butyrate belongs to a well-known class of epigenetic factors known as histone deacetylase inhibitors (HDACi) [4]. Histone deacetylases (HDACs) are critical enzymes involved in epigenetic transcriptional regulation, i.e., histone acetylation associated with chromatin structure and function [57,58].There are very compelling data showing that sodium butyrate increases the quantities of acetylated H3 and H4 core histone proteins in certain cells and tissues [59–62]. However, very limited evidence can be found in the literature regarding butyrate-induced histone acetylation *in vivo*. The only data available were obtained in chicken, mice, and pigs [7,63,64,65]; hence, the present study represents the first in fish. Our results on sea bass clearly confirmed the capability of butyrate to induce histone hyperacetylation even *in vivo*. In agreement with what Mátis and colleagues [7] observed in liver of chickens fed a low dose of butyrate (0.25 g/kg body weight, BW), no significant differences were found in the acetylation state of total histone H3 at lysine 9 after the dietary administration of 2 g/kg feed of Na-butyrate in sea bass. Interestingly, a higher dose of butyrate (1.25 g/kg BW) caused, instead, a relevant increase in H3 acetylation ratio in chicken [7]. This indicated that the level of histone H3 acetylation was dose-dependent and therefore the failed hyperacetylation observed in sea bass fed butyrate could be explained by the amount of Na-butyrate in the diet (2 g/kg feed), which was perhaps not sufficient to induce histone H3 hyperacetylation. Moreover, likewise in chicken [66], two isoforms of histone H3 were separated on the immunoblots in sea bass; in mammals, in contrast, three H3 variants have been characterized (H3.1, H3.2, H3.3) [67].

Butyrate treatment undoubtedly induced an increase of histone H4 acetylation in sea bass liver. In chicken, hyperacetylation of histone H4 occurred independently of the dietary intake levels of butyrate [7]. Similarly, acetylation of histone H4 in mammals [64] seemed to be independent of the butyrate dose, since both low and high diet content of Na-butyrate increased acetylated H4 levels in mouse hippocampus. Furthermore, in functional studies such as transcription factor-binding assays or gene expression analysis, acetylation of histone H4 was often found to be inversely correlated with acetylation of H3 [68–70]. Therefore, it would not be surprising if histone H3 and H4 differ from each other in response to dietary butyrate and this could be tested in a future research.

Among all core histones, H2A has the largest number of variants. In mammalian Jurkat cells, at least thirteen H2A variants were identified [71]. According to Brower-Toland et al., [72], and Ishibashi et al., [73] acetylation of H2A is involved in conformational changes of nucleosomes, which influence some strong, specific, and key histone-DNA interactions. In contrast, Gansen et al. [70] suggested that acetylation of H2A and H2B histones did not influence nucleosome stability, but could instead affect the nucleosome entry-exit region. However, multiple studies revealed that butyrate caused hyperacetylation of H2A both *in vivo* [7] and in cell culture [60,73,74]. We could not verify in sea bass whether butyrate induced H2A hyperacetylation since the antibody we used did not recognized our species epitope. However, we found that dietary butyrate caused a significant decrease in the total amount of H2A histone in European sea bass hepatocytes.

Concerning gene transcript abundance analysis, this study clearly showed tissue-dependent differences in the expression of five target genes involved in epigenetic regulatory mechanisms [75]; the expression was in general, higher in the liver than in the intestine. As previously found in European sea bass reared in different temperatures [36], three of target genes (*dicer1*, *ehmt2*, and *hdac11*) exhibited increased expression in the liver as a consequence of butyrate treatment, suggesting that these genes are involved in physiological processes in charge of coping with external insults.

The Dicer1 family is known to participate in the innate immune response to pathogens, mainly in RNA silencing-based antiviral immunity [76,77]. Indeed, studies in the past twenty years have established a completely new RNA-based immune system against viruses that is mechanistically related to RNA silencing or RNA interference. This viral immunity begins with recognition of viral double-stranded or structured RNA by the Dicer nuclease family of host immune receptors. Moreover, *dicer1* knockdown experiments showed an increase in the interferon response against pathogens [77]. Although our results showed a slightly increase in the expression of *irf1*, a higher expression of *dicer1* was also observed in the liver, suggesting that in butyrate-treated fish dicer 1 was inhibiting an interferon response against the external insult.

The higher expression of *ehmt2* found in both tissues due to butyrate treatment could probably be related to the histone H3 dimethylation of lysine residue 10, as this is the expected effect of this enzyme. As demonstrated previously, this creates an epigenetic mark on nucleosomes associated to the *il6* promoter that may repress its expression and alter the *il6* signaling pathway [78]. A similar effect is possible in our experiment with butyrate treatment since *il6* expression was downregulated (although not significantly) in both the intestine and liver.

Finally, *hdac11* has also been related to the immune system by downregulating the expression of *il10* in antigen-presenting cells [79]. Overexpression of *hdac11* is thought to inhibit *il10* expression and activate T-cell responses. Our results in intestine showed a decrease in *hdac11* expression and a slight increase in *il10* levels. This suggests that, in butyrate-treated fish, antigen-specific T-cell responses could be impaired, which probably activates immune tolerance. This situation is known to prevent self-tissue damage [80] and the scenario fits nicely with the known anti-inflammatory effect of butyrate in the fish that received the supplemented diet.

## Conclusions

Results of the 8-week-long feeding trial showed no significant differences in weight gain and SGR of sea bass that received 0.2% sodium butyrate supplementation in the diet in comparison to control fish that received a diet without Na-butyrate.

Butyrate in the feed significantly increased the acetylation state of histone H4 at lysine 8, leading to a twofold increase in comparison to the control group, but no changes were found in the acetylation of histone H3 at Lys9. Interestingly, for histone H3 two different isoforms were separated on the immunoblots, which could correspond to H3.1 and H3.2 isoforms previously found in terrestrial animals.

Concerning gene expression, butyrate applied as a nutritional supplement caused significant changes *in vivo* in the expression of genes related to epigenetic regulatory mechanisms such as *hdac11*, *ehmt2*, and *dicer1*. Statistical analysis by two-way ANOVA for these genes showed significant differences due to the butyrate treatment (*P* = 0.002) and to the interaction between tissue and treatment (*P* = 0.010). The expression of four (*il1 β*, *il8*, *irf1*, and *tnf*α) out of seven target genes related to mucosal protection and inflammatory response was significantly different between the two analyzed tissues but only for the *il10* gene were differences observed in the expression (*P* = 0.003) due to the butyrate treatment. Thus, in this study we reveal some of the effects of butyrate supplementation. This information is essential for the development of substitution diets in the efforts to improve the sustainability of the aquaculture of carnivorous species.

## Supporting Information

S1 Fig Multiple sequence alignment between the human peptide sequence used for the production of “Acetyl-Histone H4 (Lys8) Antibody #2594”, and the ortholog sequences in European sea bass (*Dicentrarchus labrax*) and other teleost fish. (PDF)

S2 Fig Genome position of all the primers used in the study. (PDF)

S1 Table Quantitative real time PCR primer characteristics. (PDF)

S2 Table Statistical analysis by two-way ANOVA of fish growth data. (PDF)

S3 Table Quantitative real time PCR: fold changes (FC) in the expression of genes related to epigenetic regulatory mechanisms and statistical analysis. (PDF)

S4 Table Quantitative real time PCR: fold changes (FC) in the expression of genes related to inflammatory response, mucosal protection, and immune homeostasis plus statistical analysis. (PDF)

## References

[1] Bergman FN. Energy contributions of volatile fatty acids from the gastrointestinal tract in various species. Physiol Rev. 1990; 70:567–590. 2181501 10.1152/physrev.1990.70.2.567

[2] Cummings JH, Macfarlane GT. The Control and Consequences of Bacterial Fermentation in the Human Colon. Journal of Applied Bacteriology. 1991; 70(6):443–459. 1938669 10.1111/j.1365-2672.1991.tb02739.x

[3] Guilloteau P, Martin L, Eeckhaut V, Ducatelle R, Zabielski R, Van Immerseel F. From the gut to the peripheral tissues: the multiple effects of butyrate. Nutr Res Rev. 2010; 23:366–384. 10.1017/S0954422410000247 20937167

[4] Canani RB, Di Costanzo M, Leone L. The epigenetic effects of butyrate: potential therapeutic implications for clinical practice. Clinical Epigenetics. 2012; 4(1):4 10.1186/1868-7083-4-4 22414433 PMC3312834

[5] Louis P, Flint HJ. Diversity, metabolism and microbial ecology of butyrate-producing bacteria from the human large intestine. FEMS Microbiol Lett. 2009; 294:1–8. 10.1111/j.1574-6968.2009.01514.x 19222573

[6] Macfarlane S, Macfarlane GT. Regulation of short-chain fatty acid production. The Proceedings of the Nutrition Society. 2003; 62(1):67–72. 12740060 10.1079/PNS2002207

[7] Mátis G, Neogrády Z, Csikó G, Kulcsár A, Kenéz Á, Huber K. Effects of orally applied butyrate bolus on histone acetylation and cytochrome P450 enzyme activity in the liver of chicken–a randomized controlled trial. Nutrition & Metabolism. 2013; 10:12. 23336999 10.1186/1743-7075-10-12 PMC3561214

[8] Gálfi P, Neogrády S. The pH-dependent inhibitory action of n-butyrate on gastrointestinal epithelial cell division. Food Res Int. 2002; 34:581–586.

[9] Wong JMWRD, de Souza RRD, Kendall CWC, Emam A, Jenkins DJA. Colonic Health: Fermentation and Short Chain Fatty Acids. Journal of Clinical Gastroenterology. 2006; 40(3):235–243. 16633129 10.1097/00004836-200603000-00015

[10] Vinolo MA, Hatanaka E, Lambertucci RH, Curi R. Effects of short chain fatty acids on effector mechanisms of neutrophils. Cell Biochem Funct. 2009; 27(1):48–55. 10.1002/cbf.1533 19107872

[11] Toden S, Bird AR, Topping DL, Conlon MA. Dose-dependent reduction of dietary protein-induced colonocyte DNA damage by resistant starch in rats correlates more highly with caecal butyrate than with other short chain fatty acids. Cancer Biol Ther. 2007; 6(2):e1–e6. 10.4161/cbt.6.2.3627 17218781

[12] Hamer HM, Jonkers D, Venema K, Vanhoutvin S, Troost FJ, Brummer RJ. Review article: the role of butyrate on colonic function. Alimentary Pharmacology & Therapeutics (Aliment Pharmacol Ther). 2008; 27:104–119. 17973645 10.1111/j.1365-2036.2007.03562.x

[13] Canani RB, Costanzo MD, Leone L, Pedata M, Meli R, Calignano A. Potential beneficial effects of butyrate in intestinal and extraintestinal diseases. World J Gastroenterol. 2011; 17:1519–28. 10.3748/wjg.v17.i12 21472114 PMC3070119

[14] Biancotto C, Frigè G, Minucci S. Histone modification therapy of cancer. Adv Genet. 2010; 70:341–386. 10.1016/B978-0-12-380866-0.60013-7 20920755

[15] Scollo G, Terova G, Rimoldi S, Bernardini G, Antonini M, Saroglia M. Does butyrate have a role in the protection of fish intestine? Results from a preliminary study on European sea bass (*D*. *labrax*) Aquaculture Europe. Book of abstracts. Prague. 2012.

[16] Robles R, Lozano AB, Sevilla A, Márquez L, Nuez-Ortín W, Moyano FJ. Effect of partially protected butyrate used as feed additive on growth and intestinal metabolism in sea bream (*Sparus aurata*). Fish Physiol. Biochem. 2013; 39: 1567–1580. 10.1007/s10695-013-9809-3 23737146

[17] Björkholm B, Bok CM, Lundin A, Rafter J, Hibberd ML, Pettersson S. Intestinal microbiota regulate xenobiotic metabolism in the liver. PLoS ONE. 2009; 4:e6958 10.1371/journal.pone.0006958 19742318 PMC2734986

[18] Bloemen JG, Venema K, van de Poll MC, Olde Damink SW, Buurman WA, Dejong CH. Short chain fatty acids exchange across the gut and liver in humans measured at surgery. Clin Nutr. 2009; 28:657–661. 10.1016/j.clnu.2009.05.011 19523724

[19] Demigné C, Yacoub C, Rémésy C. Effects of absorption of large amounts of volatile fatty acids on rat liver metabolism. J Nutr. 1986; 116:77–86. 3003291 10.1093/jn/116.1.77

[20] Beauvieux MC, Tissier P, Gin H, Canioni P, Gallis JL. Butyrate impairs energy metabolism in isolated perfused liver of fed rats. J Nutr. 2001; 131:1986–1992. 11435518 10.1093/jn/131.7.1986

[21] Gallis JL, Tissier P, Gin H, Beauvieux MC. Decrease in oxidative phosphorylation yield in presence of butyrate in perfused liver isolated from fed rats. BMC Physiol. 2007; 7:8 17725817 10.1186/1472-6793-7-8 PMC2048500

[22] Gallis JL, Gin H, Roumes H, Beauvieux MC. A metabolic link between mitochondrial ATP synthesis and liver glycogen metabolism: NMR study in rats re-fed with butyrate and/or glucose. Nutr Metab. 2011; 8:38. 10.1186/1743-7075-8-38 PMC3141389 21676253

[23] Evans JA. The effects of diet and feeding frequency on peripheral nutrient supply and growth traits of the lamb. PhD Thesis. University of Nottingham. 1998. Available: http://eprints.nottingham.ac.uk/14420/.

[24] Topping DL, Clifton PM. Short-chain fatty acids and human colonic function: roles of resistant starch and non-starch polysaccharides. Physiol Rev. 2001; 81: 1031–64. 11427691 10.1152/physrev.2001.81.3.1031

[25] Miller SJ. Cellular and physiological effects of short-chain fatty acids. Mini Rev Med Chem. 2004; 4(8):839–845. 15544545 10.2174/1389557043403288

[26] Gálfi P, Bokori J. Feeding trial in pigs with a diet containing sodium n-butyrate. Acta Vet Hung. 1990; 38(1–2):3–17. 2100936

[27] Kotunia A, Wolinski J, Laubitz D, Jurkowska M, Rome V, Guilloteau P, Zabielski R. Effect of sodium butyrate on the small intestine development in neonatal piglets fed correction of feed by artificial sow. J Physiol Pharmacol. 2004; 55 Suppl 2:59–68. 15608361

[28] Hu Z, Guo Y. Effects of dietary sodium butyrate supplementation on the intestinal morphological structure, absorptive function and gut flora in chickens. Anim Feed Sci Tech. 2007; 132:240–249.

[29] Owen MAG, Waines P, Bradley G, Davies S. The effect of dietary supplementation of sodium butyrate on the growth and microflora of Clarias gariepinus (Burchell, 1822). XII international symposium fish nutrition and feeding. 2006 May 28–June 1; Book of abstracts: pp 149.

[30] Zheng, RG. The effect of sodium butyrate on the growth performance and intestinal mucous structure of fresh water fish. PhD thesis (2009). Available: http://www.globethesis.com/?t=2143360272979206.

[31] Bjerkeng B, Storebakken T, Wathne E. Cholesterol and short-chain fatty acids in diets for Atlantic salmon *Salmo salar* (L.): effects on growth, organ indices, macronutrient digestibility and fatty acid composition. Aquaculture Nutrition. 1999; 5:181–191.

[32] Gao Y, Storebakken T, Shearer KD, Penn M, Øverland M. Supplementation of fishmeal and plant protein-based diets for rainbow trout with a mixture of sodium formate and butyrate. Aquaculture. 2011; 311: 233–240.

[33] Locksley RM, Killeen N, Lenardo MJ. The TNF and TNF receptor superfamilies: integrating mammalian biology. Cell. 2001; 104(4):487–501. 11239407 10.1016/s0092-8674(01)00237-9

[34] Brien JD, Daffis S, Lazear HM, Cho H, Suthar MS, Gale M Jr, et al Interferon Regulatory Factor-1 (IRF-1) Shapes Both Innate and CD8+ T Cell Immune Responses against West Nile Virus Infection. PLoS Pathog. 2011; 7(9): e1002230 10.1371/journal.ppat.1002230 21909274 PMC3164650

[35] Allen A, Hutton DA, Pearson JP. The MUC2 gene product: a human intestinal mucin. Int J Biochem Cell Biol. 7 1998; 30(7):797–801. 9722984 10.1016/s1357-2725(98)00028-4

[36] Díaz N, Piferrer F. Lasting effects of early exposure to temperature on the gonadal transcriptome at the time of sex differentiation in the European sea bass, a fish with mixed genetic and environmental sex determination. BMC Genomics. 2015 9 4;16(1):679. 26338702 10.1186/s12864-015-1862-0 PMC4560065

[37] Jaskiewicz L, Filipowicz W: Role of Dicer in posttranscriptional RNA silencing. Current Topics in Microbiology and Immunology. 2008; 320:77–97. 18268840 10.1007/978-3-540-75157-1_4

[38] Nishio H, Walsh MJ. CCAAT displacement protein/cut homolog recruits G9a histone lysine methyltransferase to repress transcription. PNAS. 2004; 101:11257–11262. 15269344 10.1073/pnas.0401343101 PMC509191

[39] Duan Z, Zarebski A, Montoya-Durango D, Grimes HL, Horwitz M. Gfi1 coordinates epigenetic repression of p21Cip/WAF1 by recruitment of histone lysine methyltransferase G9a and histone deacetylase 1. Molecular and Cellular Biology. 2005; 25: 10338–10351. 16287849 10.1128/MCB.25.23.10338-10351.2005 PMC1291230

[40] Guo WJ, Datta S, Band V, Dimri GP. Mel-18, a polycomb group protein, regulates cell proliferation and senescence via transcriptional repression of Bmi-1 and c-Myc oncoproteins. Molecular Biology of the Cell. 2007; 18:536–546. 17151361 10.1091/mbc.E06-05-0447 PMC1783768

[41] Gao L, Cueto MA, Asselbergs F, Atadja P. Cloning and functional characterization of HDAC11, a novel member of the human histone deacetylase family. The Journal of Biological Chemistry. 2002; 277:25748–25755. 11948178 10.1074/jbc.M111871200

[42] Kim TG, Kraus JC, Chen J, Lee Y. JUMONJI, a critical factor for cardiac development, functions as a transcriptional repressor. The Journal of Biological Chemistry. 2003; 278: 42247–42255. 12890668 10.1074/jbc.M307386200

[43] Tine M, Kuhl H, Gagnaire P-A, Louro B, Desmarais E, Martins RS, et al European sea bass genome and its variation provide insights into adaptation to euryhalinity and speciation. Nature Commun. 2014; 5:5770 10.1038/ncomms6770 25534655 PMC4284805

[44] Schmittgen TD, Livak KJ. Analyzing real-time PCR data by the comparative CT method. Nature Protocols. 2008; 3(6):1101–1108. 18546601 10.1038/nprot.2008.73

[45] Tacon AGJ, Metian M. Global overview on the use of fish meal and fish oil in industrially compounded aquafeeds: trends and future perspectives. Aquaculture. 2008; 285: 146–158.

[46] Hardy RW. Utilization of plant proteins in fish diets: effects of global demand and supplies of fishmeal. Aquacult. Res. 2010; 41: 770–776.

[47] Benedito-Palos L, Ballester-Lozano GF, Simó P, Karalazos V, Ortiz Á, Calduch-Giner J, Pérez-Sánchez J. Lasting effects of butyrate and low FM/FO diets on growth performance, blood haematology/biochemistry and molecular growth-related markers in gilthead sea bream (Sparus aurata). Aquaculture. 2016; 454: 8–18.

[48] Øverland M, Granli T, Kjos NP, Fjetland O, Steien SH, Stokstad M. Effect of dietary formates on growth performance, carcass traits, sensory quality, intestinal microflora, and stomach alterations in growing–finishing pigs. Journal of Animal Science. 2000; 78:1875–1884. 10907830 10.2527/2000.7871875x

[49] Øverland M, Kjos NP, Borg M, Skjerve E, Sørum H. Organic acids in diets for entire male pigs: effect on skatole level, microbiota in digesta, and growth performance. Livestock Science. 2008; 115:169–178.

[50] Lückstädt C. The use of acidifiers in fish nutrition. CAB Reviews: Perspectives in Agriculture Veterinary Science. Nutr and Nat Resour. 2008; 3(044):1–8.

[51] De Wet L. Organic acids as performance enhancers. Aqua Feeds: Formulation and Beyond. 2005; 2:12–14.

[52] Pandey A, Satoh S. Effects of organic acids on growth and phosphorus utilization in rainbow trout *Oncorhynchus mykiss*. Fisheries Science. 2008; 74:867–874. 25522509

[53] Vielma J, Ruohonen K, Lall SP. Supplemental citric acid and particle size of fish bone-meal influence the availability of minerals in rainbow trout *Onchorynchus mykiss* (Walbaum). Aquaculture Nutrition. 1999; 5:65–71.

[54] Zhou Z, Liu Y, He S, Shi P, Gao X, Yao B, Ringø E. Effects of dietary potassium diformate (KDF) on growth performance, feed conversion and intestinal bacterial community of hybrid tilapia (*Oreochromis niloticus*×*O*. *aureus*). Aquaculture. 2009; 291:89–94.

[55] Gislason G, Olsen RE, RingØ E. Lack of growth-stimulating effect of lactate on Atlantic salmon, *Salmo salar* L. Aquaculture and Fisheries Management. 1994; 25:861–862.

[56] Liu W, Yang Y, Zhang J, Gatlin DM, Ringø E, Zhou Z. Effects of dietary microencapsulated sodium butyrate on growth, intestinal mucosal morphology, immune response and adhesive bacteria in juvenile common carp (*Cyprinus carpio*) pre-fed with or without oxidized oil. Br J Nutr. 2014; 112:15–29. 10.1017/S0007114514000610 24774835

[57] Dokmanovic M, Marks PA. Prospects: histone deacetylase inhibitors. J Cell Biochem. 2005; 96:293–304. 16088937 10.1002/jcb.20532

[58] Waterborg JH. Dynamics of histone acetylation *in vivo*. A function for acetylation turnover? Biochim Cell Biol. 2002; 80:363–78. 10.1139/o02-080 12123289

[59] Candido EPM, Reeves R, Davie JR. Sodium butyrate inhibits histone deacetylation in cultured cells. Cell. 1978; 14:105–113. 667927 10.1016/0092-8674(78)90305-7

[60] Mohana KB, Song HJ, Cho SK, Balasubramanian S, Choe SY, Rho GJ. Effect of histone acetylation modification with sodium butyrate, a histone deacetylase inhibitor, on cell cycle, apoptosis, ploidy and gene expression in porcine fetal fibroblasts. Journal of Reproduction and Development. 2007; 53:903–913. 17558190 10.1262/jrd.18180

[61] Koprinarova M, Markovska P, Iliev I, Anachkova B, Russev G. Sodium butyrate enhances the cytotoxic effect of cisplatin by abrogating the cisplatin imposed cell cycle arrest. BMC Molecular Biology. 2010; 11:49 10.1186/1471-2199-11-49 20576112 PMC2906439

[62] Paskova L, Trtkova KS, Fialova B, Benedikova A, Langova K, Kolar Z. Different effect of sodium butyrate on cancer and normal prostate cells. Toxicology in Vitro. 2013; 27:1489–1495. 10.1016/j.tiv.2013.03.002 23524101

[63] Shimazu T, Hirschey MD, Newman J, He W, Shirakawa K, Le Moan N, Grueter CA, Lim H, Saunders LR, Stevens RD, Newgard CB, Farese RV Jr, de Cabo R, Ulrich S, Akassoglou K, Verdin E. Suppression of oxidative stress by *b*-hydroxybutyrate, an endogenous histone deacetylase inhibitor. Science. 2013; 339: 211 10.1126/science.1227166 23223453 PMC3735349

[64] Gundersen BB, Blendy JA. Effects of the histone deacetylase inhibitor sodium butyrate in models of depression and anxiety. Neuropharmacology. 2009; 57:67–74. 10.1016/j.neuropharm.2009.04.008 19393671 PMC2702471

[65] Kien CL, Peltier CP, Mandal S, Davie JR, Blauwiekel R. Effects of the *in vivo* supply of butyrate on histone acetylation of cecum in piglets. JPEN-Parenter Enter Nutr. 2008; 32:366–384. 10.1177/014860710803200151 PMC2258555 18165447

[66] Zhang K, Tang H, Huang L, Blankenship JW, Jones PR, Xiang F, Yau PM, Burlingame AL. Identification of acetylation and methylation sites of histone H3 from chicken erythrocytes by high-accuracy matrix-assisted laser desorption ionization-time-of-flight, matrix-assisted laser desorption ionization-postsource decay, and nanoelectrospray ionization tandem mass spectrometry. Analytical Biochemistry. 2002; 306:259–269. 12123664 10.1006/abio.2002.5719

[67] Hake SB, Garcia BA, Duncan EM, Kauer M, Dellaire G, Shabanowitz J, Bazett-Jones DP, Allis CD, Hunt DF. Expression patterns and post-translational modifications associated with mammalian histone H3 variants. J Biol Chem. 2006; 281:559–568. 16267050 10.1074/jbc.M509266200

[68] Kurdistani SK, Tavazoie S, Grunstein M. Mapping global histone acetylation patterns to gene expression. Cell. 2004; 117:721–733. 15186774 10.1016/j.cell.2004.05.023

[69] Agricola E, Verdone L, Di Mauro E, Caserta M. H4 acetylation does not replace H3 acetylation in chromatin remodeling and transcription activation of Adr1-dependent genes. Mol Microbiol. 2006; 62:1433–1446. 17121596 10.1111/j.1365-2958.2006.05451.x

[70] Gansen A, Tóth K, Schwarz N, Langowski J. Opposing roles of H3- and H4-acetylation in the regulation of nucleasome structure-a FRET study. Nucleic Acid Research. 2015; 43(3):1433–1443. 10.1093/nar/gku1354 PMC4330349 25589544

[71] Bonenfant D, Coulot M, Towbin H, Schindler P, van Oostrum J. Characterization of histone H2A and H2B variants and their post-translational modifications by mass spectrometry. Mol Cell Proteomics. 2006; 5:541–552. 16319397 10.1074/mcp.M500288-MCP200

[72] Brower-Toland B, Wacker DA, Fulbright RM, Lis JT, Kraus WL, Wang MD. Specific contributions of histone tails and their acetylation to the mechanical stability of nucleosomes. J Mol Biol. 2005; 346:135–146. 15663933 10.1016/j.jmb.2004.11.056

[73] Ishibashi T, Dryhurst D, Rose KL, Shabanowitz J, Hunt DF, Ausió J. Acetylation of vertebrate H2A.Z and its effects on the structure of nucleosome. Biochemistry. 2009; 48: 5007–5017. 10.1021/bi900196c 19385636 PMC2850812

[74] Tobisawa Y, Imai Y, Fukuda M, Kawashima H. Sulfation of colonic mucins by N-acetylglucosamine 6-O-sulfotransferase-2 and its protective function in experimental colitis in mice. J Biol Chem. 2010; 285:6750–6760. 10.1074/jbc.M109.067082 20018871 PMC2825469

[75] Piferrer F. Epigenetics of sex determination and gonadogenesis. Developmental Dynamics. 2013; 242:360–370. 10.1002/dvdy.23924 23335256

[76] Aliyari R, Ding SW. RNA-based viral immunity initiated by the Dicer family of host immune receptors. Immunology Review. 2009; 227(1):176–188. 10.1111/j.1600-065X.2008.00722.x PMC2676720 19120484

[77] Chiappinelli KB, Haynes BC, Brent MR, Goodfellow PJ. Reduced DICER1 elicits an interferon response in endometrial cancer cells. Molecular Cancer Research. 2012; 10: 316–325. 10.1158/1541-7786.MCR-11-0520 22252463 PMC3307918

[78] Takahashi H, Katayama K, Sohya K, Miyamoto H, Prasad T, Matsumoto Y, Ota M, Yasuda H, Tsumoto T, Aruga J, Craig AM. Selective control of inhibitory synapse development by Slitrk3-PTPd trans-synaptic interaction. Nature Neurosci. 2012; 15: 389–400. 10.1038/nn.3040 22286174 PMC3288805

[79] Villagra A, Cheng F, Wang HW, Suarez I, Glozak M, Maurin M, Nguyen D, Wright KL, Atadja PW, Bhalla K, Pinilla-Ibarz J Seto E, Sotomayor EM. The histone deacetylase HDAC11 regulates the expression of interleukin 10 and immune tolerance. Nature Immunology. 2009; 10(1): 92–100. 10.1038/ni.1673 19011628 PMC3925685

[80] Rubtsov YP, Rasmussen JP, Chi EY, Fontenot J, Castelli L, Ye X, Treuting P, Siewe L, Roers A, Henderson WR Jr, Muller W, Rudensky AY. Regulatory T cell-derived interleukin-10 limits inflammation at environmental interfaces. Immunity. 2008; 28:546–558. 10.1016/j.immuni.2008.02.017 18387831

