# Supplementary material for: Effects of Sodium Butyrate Treatment on Histone Modifications and the Expression of Genes Related to Epigenetic Regulatory Mechanisms and Immune Response in European Sea Bass (Dicentrarchus Labrax) Fed a Plant-Based Diet
Source: PLoS One. 2016 Jul 29;11(7):e0160332. doi: 10.1371/journal.pone.0160332 (PMC4966935; doi:10.1371/journal.pone.0160332)
Supplement: S1 Fig — (PDF) [file pone.0160332.s001.pdf]

**S1 Fig.** Multiple sequence alignment between the S1human peptide sequence used for the production of "Acetyl-Histone H4 (Lys8) Antibody #2594", and the ortholog sequences in European sea bass (*Dicentrarchus labrax*) and other teleost fish.

```
>sp|P62805|H4_HUMAN Histone H4 OS=Homo sapiens GN=HIST1H4A PE=1 SV=2
MSGRGKGGKGLGKGGAKRHRKVLRDNIQGITKPAIRRLARRGGVKRISGLIYEETRGVLKVFLENVIRDAVTYTE
HAKRKTVTAMDVVYALKRQGRTLYGFGG

>Dicentrarchus labrax (European seabass) Agn_00220090 (sea bass genome
(http://seabass.mpipz.de/).
MSGRGKGGKGLGKGGAKRHRKVLRDNIQGITKPAIRRLARRGGVKRISGLIYEETRGVLKVFLENVIRDAVTYTE
HAKRKTVTAMDVVYALKRQGRTLYGFGG

>gi|528486546|ref|XP_005172457.1| PREDICTED: histone H4 [Danio rerio]
MSGRGKGGKGLGKGGAKRHRKVLRDNIQGITKPAIRRLARRGGVKRISGLIYEETRGVLKVFLENVIRDAVTYTE
HAKRKTVTAMDVVYALKRQGRTLYGFGG

>gi|64427|emb|CAA38015.1| histone H4 [Oreochromis niloticus]
MSGRGKGGKGLGKGGAKRHRKVLRDNIQGITKPAIRRLARRGGVKRISGLIYEETRGVLKVFLENVIRDAVTYTE
HAKRKTVTAMDVVYALKRQGRTLYGFGG

>gi|929326370|ref|XP_014046901.1| PREDICTED: histone H4 [Salmo salar]
MSGRGKGGKGLGKGGAKRHRKVLRDNIQGITKPAIRRLARRGGVKRISGLIYEETRGVLKVFLENVIRDAVTYTE
HAKRKTVTAMDVVYALKRQGRTLYGFGG

>gi|831567592|ref|XP_012733759.1| PREDICTED: histone H4 [Fundulus heterocli
tus]
MSGRGKGGKGLGKGGAKRHRKVLRDNIQGITKPAIRRLARRGGVKRISGLIYEETRGVLKVFLENVIRDAVTYTE
HAKRKTVTAMDVVYALKRQGRTLYGFGG
```

```
 CLUSTAL O(1.2.2) multiple sequence alignment

 H.sapiens           MSGRGKGGKGLGKGGAKRHRKVLRDNIQGITKPAIRRLARRGGVKRISGLIYEETRGVLK
 D.labrax            MSGRGKGGKGLGKGGAKRHRKVLRDNIQGITKPAIRRLARRGGVKRISGLIYEETRGVLK
 D.rerio             MSGRGKGGKGLGKGGAKRHRKVLRDNIQGITKPAIRRLARRGGVKRISGLIYEETRGVLK
 O.niloticus         MSGRGKGGKGLGKGGAKRHRKVLRDNIQGITKPAIRRLARRGGVKRISGLIYEETRGVLK
 S.salar             MSGRGKGGKGLGKGGAKRHRKVLRDNIQGITKPAIRRLARRGGVKRISGLIYEETRGVLK
 F.heteroclitus      MSGRGKGGKGLGKGGAKRHRKVLRDNIQGITKPAIRRLARRGGVKRISGLIYEETRGVLK
                     ************************************************************


 H.sapiens           VFLENVIRDAVTYTEHAKRKTVTAMDVVYALKRQGRTLYGFGG
 D.labrax            VFLENVIRDAVTYTEHAKRKTVTAMDVVYALKRQGRTLYGFGG
 D.rerio             VFLENVIRDAVTYTEHAKRKTVTAMDVVYALKRQGRTLYGFGG
 O.niloticus         VFLENVIRDAVTYTEHAKRKTVTAMDVVYALKRQGRTLYGFGG
 S.salar             VFLENVIRDAVTYTEHAKRKTVTAMDVVYALKRQGRTLYGFGG
 F.heteroclitus      VFLENVIRDAVTYTEHAKRKTVTAMDVVYALKRQGRTLYGFGG
                     *******************************************
```
