# Supplementary material for: Effects of Sodium Butyrate Treatment on Histone Modifications and the Expression of Genes Related to Epigenetic Regulatory Mechanisms and Immune Response in European Sea Bass (Dicentrarchus Labrax) Fed a Plant-Based Diet
Source: PLoS One. 2016 Jul 29;11(7):e0160332. doi: 10.1371/journal.pone.0160332 (PMC4966935; doi:10.1371/journal.pone.0160332)
Supplement: S2 Fig — (PDF) [file pone.0160332.s002.pdf]

**S2 Fig**. Genome position of all the primers used in the study

## * ***il1β*** **_European sea bass:**

>dicLab1_genemodels_DLAgn_00122200 | interleukin-1 beta | IL-1BETA | -
range=LG20:18233133-18237725 5'pad=0 3'pad=0 strand=- repeat Masking=none
CATGTGGAAAGCCATGTTGCATTTATGTATGCGTCCAGTGGGGCATCACA
ATAAAATCAGGTATAATGTGTTAATTCCACAATAGGAGATATGTTATGTT
GTTGCCTAACAACAGTTTTGGTGTAAAAAGCCTGCATATATCGGTATCGG
AAATTGAGAGTTGGACAATATCGGAATATCGGATATTGGCAGAAAAGCCA
ATATCGAGCATCCCTACTCAGTATATAATATGAATATCATTACATTACTA
CAGCCCTTCCCCCTCCAGACAGTGTAAATCCACATCCTGTGAAGCAGGTT
GACTACAACTGTGAGTGACGTAGCTTTGCTTTGAAACACAGCATGACAGG
GAAGTGAGGAAATATCCAATTCTGCCACTGTTACGCAATTATAAAATACA
AGCAAGTCTACTACGGCTGGTGGAATTTTTTCCCATGTAAAGAACCACAA
AACCCCAAAGGGCCTCTTCAAAGACGTAATATTAGGAAATAGGTGATAAG
CATTTCAAGCTGTACTTTTTGTGTATTTTTGTGTATTTCAGAGATAAACT
TTTGACTAAGCAATATCTTAAGTAATATTTATCATTATTAGACCCTTTAC
ACCAGGGGTCTCAAACTCAAATTTTAGGGCCACTGCTGGTATTGTCAGGG
CTACTTTTTTCACATACTAAACAAGAAAATGCATACAATCTATACATTCT
TATATATATATATATATATATATATATAAAGCCCTGAAACCTGCTCTCTTCT
TATCAACCATTTGCATACATTTATGAGCACGTTTGACCTTTCTTTCCATT
ATTATTTATTTATTATTATTTATTAGTAATTTATTTTAATTGTATATGAA
GGGGTAGTTCAAGCAGAAACTTCAAGCAGATATTGCAGTATTTCATCATC
ACGTTGTACGCAGCAGAGAGGACCATCCCACTTCCTGGGGAAATCCCTTC
TTGACAGTAATGTGTACAAAAACCCTGCAGGTTAGCACAGACAGGCAGAT
CAACATTGACAGAGCAACTGTTGGGATCTTAACCAAACACACTAAAGCAA
CTTCTAATCTACTGACCTTAACAGGTAAAATGACTTTTCTTATGTTTTCT
TTTTTTCTGGAGCAGATTTTAAACACGATCTTTTGCTTTTTTAAGTTACT
TTGAGTATAGTTGTGGATTGAATCTTGTTACAGGACAGTTTCTTTAACAT
TTACAGCCTGTTGTGTCATTAAAAACTGCTAGAAGATCTATTCTGTCGGA
TGAAAACCTTTTTACTATGATATGGCAGCTGTACAAGCACCTTGCAGTCA
CATGCTGATACAGTAGTGAGTCATGAATCACTGCAGCAGCAGACATAGAT
GTAGCCTTTACAACAGAGAGTAAAAGGATAAATAATTATATTCAAGGAAA
GGAAATGTAATAACAATGGAAACAGGCTAATAAACAGGTAGAGTTAAATT
TGAAAATCTAGCTACATTGCCTGAAGTTGCTTTCTGTCTTCCTACAGAAA
CATGGAATCTGAGATGAAATGCAACATGAGCGAGATGTGGAGATCCAAGA
TGCCCCAGGGACTGGACTTGGAGATTACCCACCACCCACTGACAATGAGG
CGCGTGGTCAACCTCATCATTGCCATGGAGAGACTGAAGGGCTTCAGTTC
AGAGACACTGATGAGCACTGAGTTCAGAGATGAAAACCTGCTAAACATCA
TGCTGGAGAGCATAGTGGAAGGTAATCACACAGACAAGACTTAAACGCTA
TTGCATTAAAAAGCCCTGGACTGTTTTCTCTGTAACCCATCCTGCCGTAA
GTCCTCCTAAAGGAACTGTGGGAAGTTGAAGGGAAGTGAACAGTTCAGTT
ACAGATCCTGTTAGTCTAACTTTAAAGTTTATTATTTTCTGTAACTGGAA
ACCAATTGAACTAACTGTAACACCTGTGAGTGAAGCCCTTATGTCCCACA
CTCAGTCAGACTGTATATTGCTATATCTCTATACATCTCTATTATTGTCC
TGTTGTCTGCTGTAACTAATATGTGGGTGTTTAGGGTTTGTTAAAAGGAC
TTTTACTGTTCCATAAACTAAAATATTCTGTATATCTCCAGTGGGGTGAT
GAAGTTGTTGATAAACAGCAGTAAATTACTCAGATTTTTGGCCCTTCAAG
AATCAATTGCGCATGAAGAGACACAGTCTATGGCTGTTATTGCGATTCCT
GCTGCTTGTTGATAAAAAAGAAAACATACTCTGAAAAACATAACTCAACA
GATGTCAATTTTTCTTTGCCCCCATCAGAGAAAATTGTGTTTGAGCGCGG
AACAACTCCAACAGCGCAGTACAGCAAGCGACGCGAGGTCCAATGCAGCG
TGACCGACAGCGAAAAGAGGAGCTTAGTTCTGGTCCCGAACAGCATGGAG
CTCCACGCAGTGATGCTGCAGGGAG**GCTCGGACAGATGCAAAGGT**AACTA
ACTGCATGTGACAGATAGCTCAGATGGTTGGAAAATCATCCTCTGGGTCA
TGGTAGATGTTTGCAATAAGGGTTGACAGGTTTGGTTTTAGTTTATCTGA
CAATTGTTGGCTTGAGATTTTAAGCTGAAGCTGTTTAATGGTGATAATTT

ATTTATTTTT**CAAATGTAGGCCTACACCTA**AAATGTCCCCAAAATTCAGT
GTTTTCACCACTACTGCAGGATGGAAAACACACAGTAACACTAATACCGA
GCTTGTCTTGGCTCTGTTACATAGACACAGATGTGAACTTTTAGTGAAGT
GCACAGAAGACAGAGGTTGACATTGTCTTTCAGTTTTCATGAAAATGAAC
CCTTATACTCTGCTTCTCTCCTCAGTCCAACTGAACATGTCAACCTACTT
GGACCGTACACCCAGTGCTGAGGCTCAGACTGTGGCTCTGGGCATCAAGG
GCACAAATTACTACCTGTCCTGCCACAAGGATGGAGAGGAGCCAACCTTG
CATCTGGAGGTAAAAACTGTGACTCATTACACAAATCTCACATAAAGATT
ACACACTTTGTAGTCCTGCTGATTTGATCTACCTGGTCGTCTGACATGTC
ATCTTTTCACACCTCAGGTGGTGGACAAAGCCAGTCTGGCGAACATCACC
TCGGACAGCGACATGGTGCGATTTCTCTTCTACAAACAGGACTCCGGTTT
GAACATCAGCACCCTGACGTCTGTCCCCTTCAGCAACTGGTACATCAGCA
CAGCAGAGGAGAACAACCGGCCGGTGCAGATGTGCCAGGAGAGTGCCAGA
CGCCACCGAGCCTTCAACATCGACAACCTGAAAGTGGATCCAACAACTGA
GGATGAAGTTTGTCCCCTTTTGAATGGACAGTAAAATACACTGCACTTCA
GATATTTTGTATTGTGTTCAAAAAAATCAGCAACAAAGATATCTATTTTT
GTTTTAAATCACTACCACTGTTGACTAGAACGACTTCTGCAAGTTTTACA
GATTGTTTATTTACTGTATGTACCAAGTACAAAAAGTAAGATGCTTAATG
TATTGACATTGTCAAGTATCACCACAAGGTGGCATTATTGTGCTGCCTGG
TGAGCACCTTTTGTATTTATTGTGCTGAATGACATGAAACGCTATTATTG
CTTTTATCTATTAATTTATTTTATTTTAATTATTTATTAACTAATTAATA
TAATATTTAATATATATGTATCAANNNNNNNNNNNNNNNNNNNNNNNNNN
NNNNNNNNNNNNNNNNNNNNNNNNNNNNNNNNNNNNNNNNNNNNNNNNNN
NNNNNNNNNNNNNNNNNNNNNNNNTAAACTCTCAAACATGTATCTGCATA
AGTCTAGTCTAGTTTAGCCACCTGTTTATTTTATCTATAAATTGTTTCTC
AAAATGAAATTACATATATTAAGATAGATATAAAATGTCCATATTAGAGG
ACTTTCTTGATATCAGAAACCTCCAGTTAATGGATTAATTATATTAGAAG
ATCGTAATAGAATTTAAAATCATTTTGGCCAGTGTTAGCAACTACAAGAC
GTTCATTCAGCTGGTCAAATTGTGACATTTTCTTTGCAATAATAAGCTAA
TAAATTCAACACAAACAACCAATGTGGTAACCCTACTGTAATTGATGAGC
AGCTTGAGCACCAATACTAGCATTTTGCTCGACCTCTTAGAGCCTAACAG
CTCAGTTTTTATCATCAGTGACAAGCCAAAGCAGCAGAGAATTTGGTTGT
GCAAACAGCCGTTCAGCTCTGATAGCTGACTGGGTTTTTAATTTCAGTGT
GCTTCTGAGAGAAAGATCACTGTGTCTAACGATAAGAAAGATGTTTACAT
TGCATTATGGCTGTTCAAATGCTCAGAACCCATGTAATTAGCAGTTTCAT
GTATCTGTGTCTGTAGAACAAAAGAAATTGCAGTTCTGATATAAAAAAAA
ATATTATTGTTCCATCCTCCTCTGTGTAGTTCAGGTTTTATATTGTTCCT
GGCAGCTTGATTTCCCCAACACACTTGTTATGTTGTAATCCAAAATGAAT
TACTCCTTTTTGTGTTGCTGTCAAGTTTCCGCCTGTTTCTTTTATTCTAG
CCAGTGGACGAGCTGCGGACTGTGGTGTTTGAGATTCTGAAGA

**GCTCGGACAGATGCAAAGGT = forward primer (LG20:18,235,583 - 18,235,603)**
**CAAATGTAGGCCTACACCTA = reverse primer (LG20:18,235,743 - 18,235,763)**
**165bp**

## * *il10* _European sea bass:

>dicLab1_dna range=LG1A:13840252-13845522 5'pad=0 3'pad=0 strand=-repeat Masking=none
TAAATACAAAAATACACATTATATATGATTTAAAAAAATAATAAATATGG
CTTTCAAGGTTTAAAAAAAAAAAACAGTTAAAGACTCAATATGGTCAGAA
TTCTTGTCCTCAGGACAAATGTTAAATAGAAGTCATCAATGCAATCTTCT
TCCCAGGGTCACATTCAGAC**AGAGGCTGCATGGTTTCTGT**GTTGTTTAGA
AGCCATATATGTCTCAATGTAGTTGAACAGCAGACCCAGCTCGCCCATGG
CCTTATATAGACCTTTACTCTCCATCTGTGGATAAACACATGGCCCAGTT
TATTGACATCATCTGTCAAAATACAAGCATGCACACATACATGTTTTAAA
AATTTTCAGAAGTACAGAGGACATCTAGGCACTTACCT**CATCACTCTTGA**
**GCTGGTCG**GAGTGTAAGTAGAGTTTAGGGTATTTATGTCAAAATGATGCT
TGCATTTGAAGTAATGTCTCTGTAAGAAACAATAAAAAGATTATGTTAGC
AAAGGCATTACCAACTTTCTTTACACTGTCCTGCCCCTAATTAACAGCCC

CCCCTCCTCACACACTTGTAGCAAGTTAGGCAGAGCTAGAGCAACTTTGT
CAGTAGCAGCATGTCCTCACTTGTGCTTCTTCCCCTTTTGCAAGAATTTG
ATGATGTACAGTTATGCATGCTGCTGCAGTTTAGAAATCACTGACTAATG
CAAAATGATGACCTGTCCTATGAGCCATCACTGGACACACCCTTTCAGTC
GTTTGCCACATGCCAAAAAGTGCGGGGAATATTTTGAAAGTTTCCGGATG
TAGTCCAAACGAGAAGATGGTCTGGCTTGTGGCAGACCACTGTGACAGCT
AATCACGTGTATTCATGAAAAGCAAGACCAGTCGCCCCAATGTTCCTGGA
GTCCAAATATAACCTTTTAGTGCAGTGTAGCAATTGGGAAACATTTCAAA
TGTATATCCTCCAGGAGGGAATGATACACAATACGGAGGTCGATTTTAAT
TTATTTTCCTTAATAGTTATTCAACAATATCACAGCGATGCTGCACAAGG
ACAATAAAATCATCTCGTCAACGAGTGCATGTGGACAAATATGCAGCTAC
ACATTGCGCACTGGATGCTACGGAGCTCTTATGGAGCTGACTGCAGCGCG
CAGTCGGTGCGTATTTACATCTTCATTCTGCTGTTGGAGTAACACGTGTA
GCAGATACTCACACATCTGGTGAAAGATCTGCTGGATGGACTCCATGTGA
GGCTTTAAGTCCTTAGTGTCTTCCGTCACCCCGGCCATGGCTGTCGGCAG
AACCGTGCTTAGATAAAACTCCAGTATGCTGTTAATGGCGTGGCAAGCGA
ACGGGGTCTGAAAGTGGGAGGAAAGCGCCAGCCTGGTTATATGATGTCAT
TTAACTCTTTACTGGCGTGCTCGACTTCTCAGTAAATGTATGCTCTGTGC
TTCCGATTTGACTTTTAGAAGTAGCGCTTTTCTTTTTTTCCATACAGAAA
GGACTTCGTGGAGATTTAGTAGGCAAACTTACCNNNNNNNNNNNNNNNNN
NNNNNNNNNNNNNNNNNNNNNNNNNNNNNNNNNNNNNNNNNNNTTTGAAA
GAAGTGGATGATACACAGTTATACACGGTGCAGCAGTCTATGATTCATTG
ACAAAACTGGTCTTTGCTATGAGCCATCACTGAACTCTTCCCCTTCTGGT
GTGGTCAACCAGAGTTACAGAATTTGTCTCATGTCAAAGTGTGGGGAATA
TTTTGGTTTGGTTTCGATGGTCTGGCTGTTGGCAGAGTCCCAACTTAGTG
ACAGCTAATCACGTGTCTAAAAGGCATGACCACTTGGATGTTTATGTATG
CGAACAGAAACCATCTTATTTCTATTACAAAAGGAGAAATTAGGGCCAGT
ACGGCACATTGTTAATCCCTGGACTTCATATCCTCAATTTTTTGTTTTTG
TTGGCATGGGGACTACAGTATTTCTGAAATTTTGTACTTGTACTTGTATT
GATGCCCAACTACCTGCCCTCTGATCCTGGGGAACTTTTGGGACCCTCAA
GGTCTGTGGGTTCCGTGGCAGTTGCCTATTTTTCCCAGCTGGTAATCCAG
CCATGATGTATATCCCTCCTGACTCTGACGAAAGCATTGACGACAACCCC
GGAGGAGCCTGAGGAGGCAGCACAATGCCTTTGCTTCCCCTTTTGCAAGA
ACTGGATGATGCACAGTTCTGCATACTGCCGCATTTTAGGAATCACTAAC
TAAAGCAATATTAGGCTCTATCCTATGAGCCATCACTGAACTATTTCCCC
TCTGGTGTGGTCAGCCAGAGTTACAGTTTATTGACAGCTAATCATGTGTA
TACCTGCAATGCTAGTTGTTTATGTATGTTTATGTATCCAAACAGAAACC
GACTTCTTATATCTATATCATAATAGGAGCATTCAGGACCAGTACAGCTC
ATTATTATTATTATTATTATTATTATTATTATTATTATTAATTATTAATA
ATAATTATTCATCACTGGACTTCATATAAACCCAATTTAAAGATTAATTT
TACTCTTATGTTGGCATGCGGACTCCACTACAGTGTTATTTCTCCTTTCT
GGTCCTATCTGTTTTTGAGGAGTCACTTAAGTGGCTTTCAAATGTCAGTA
TTATGAAATTGGGACCGATACATACCAGAGGGAGGGGTCTGACTAGAAGA
GAAACGGTTAAACATGCTTCATACAAGTGGGGGAATTTTCTTTCCAGAAT
CATGCATTAGATATGGGCCACATAGATCTTTAGATCTTGGAAAATAATCT
GACATAGCACTTCATTTATTATCATCATCATTATATTTTTATCATCAATG
TTAGTAAAGTAGTAGTATCAGTAGTCAGTACATCAGTCCCATCTGCGGTA
CAGTAACATTGCCTTTATCTATTTTGTTTTTTTGTTAACTTATCCACAAC
CAATGTATCTCTGTATGTTCAGTTGATGACCTCTGCATAATGAAGACATC
ACGGTCTGTCCAAATAAACTTCAGGATGATTTGCCTATTCAAGCTAAGTT
CATTTACTGGATGGAGTGTGGTAGAAATTGTGATTTCCTCACTGAGAAGT
GAGCAAATAAATGATTGTAACAGTTGTATTTTGTCTATTTAATAAAATAG
AATAATAAAAGAAATAAATAGTTCAAAAGAATTACAAAAGTCAATTTGTC
TATTTCAGCTCAACAGGGTGCACACGTGGAACAGAGAGCTGTGCTGCCAA
TTTCAAGAAGACACATTCAAGACTACTTTCCAAAACTTAATTCAATTTTT
AAATGTCAAAGTATGAAAATCAAAATGAATTTTTAAAAAAGTGATCTCAT
TGTTTTGTTGACAAATTTTCCTGGCCAGCCTTGTACCTTTGTCCTCCTTT
TCGGCATCCTCTTGTAGGAAATCCAACAGGGCCCCTTTGGAGGTGTGCCT
TCTCCTTTGCGGACAGACGGAGGTGGATGGCTCATCATCTGCCTGTCTGG
TGGTGACAATACTTGTGCCAGGCTGTACAGACTCCTCCTCACCTGCCTGG
CCACTAGTTGTGACTCCACCAGGAGATGATGAGCCACTATTGCTGAAGGC

CACACCAGCCAAAATGTAAATATGCTATACTGTTTAATAAACATTCCACG
TGAGTTGGCTGACTGAAATGCTTTTGGACATGCACTGAGCTCATAGTCTA
ACCTTCTACTTTATTTTTAAATTGTCCCACTTTTTATGAAATTAGTCACT
TATCGTAAATGATCCATGTGAGGTTTTGGTTTTGAATATAGACTGTGTTT
GAATGGTATTTGCAGGCATTTAGTGGCCTTCATTTCAACCAAAACCAGAA
AACTAAAATGGTTTAGGAAAGGCTTTAAAAACAGCGATTTAATCTATGCT
TTCCTGTATTTGCCGTAGTCCATTTAGTTAGTTTTCTAATTCCAGATTCA
ACCCTGTAATGCCAAGTGTATCATATTTGATACATGAGTTTTTGAGACAT
CATCAGTGTGACCTTTTTTTTTTCTCAAAAAACCTGATGTATACAATCAG
ACACATGCAATGCACGGATAATCCACCAGGGATCAGTAATTCATGCACCA
GAGGGATTACTGGGTTAATTTACACACTTGCACCTGTTTGCTTCAAATGC
TTTGCAATGCTTAAATTTATATTTAAGCCAATACACAATCAAGTTTTATT
GTTTTATTTTAATTTCTTAATTAACATCTTTCCACATGTGTATGACCCCC
TTTGGTGTCTAGGAGCTGGGGTCTGTTTCTCAACAGAGGTTAATCTTTTA
ATTACCACGGAAGTAACTTCATCACATTTTTTGTTTCTTACTGACGTTTG
TTTGTTTTTTTTTGTTCCGATTGAAGCTTTTATCTGTTGAGGCCCCATCA
GTTTATTTCATTCAATTTTAATGTATTTTACTAAATGATTTACTATTTAA
CTGTTAACATTTAAATGTGTAAATTGTTTGCTTGGGATAGCTTTCTTTTC
ATTTTTGCCTTTTCATCAGTCTCAATCAATTATTTTTGTTTTGTTAGATG
TAGTTTGATTGTTACATGTACCGTTTTTCATGTGTTTTTGTTGCATGTGT
TTTAATCTTTCCAAACCTAAGAATGACCTCTTGGTCTCTAAATTGTGTAG
CTTCAAATTATTTTATTCCAATCCAGTCAATATAACTTATTGTTAATAGC
ATTTTTCCTGTTCTAGTAAGGTGTTTTACTATAGAATGTACAGTTTGTTT
TAAACTGCATCTTTTATTTGCATGCTGTAAATTATACAAACATACTTTAT
TTAAAATTTGACCTAAAGTATAATTTGGTAAGACATAATTTTTTTTGTAA
ACTACTCTTGCTGATATCTAAACTATAAAAATCATAGTCTCTTCCCATGC
AGTGTTAACGGCAGTGCACATTCCCCTTTTGCCCATGTGGTCTCTTCTCT
TACAACATACTTGACAAGACACACAGACAGTGACAGGACATAGCTCCTGT
GCAGAGTTAATACTACTACTACTACTACTACTACTACTACCAATAATAAT
AATAATAATAATAATAATAATAATAATAATAATAATAATATTGTGCTTTT
ACTCACATAGAAATCTTGGATCTCTGTGAAGTCTGCTCTGAGTTGCCTTA
ACATGCCAGGGAAATCCTCCACGAAATGACAGCACTGGTTGTTGCACATG
GGACTGCACCAGACAGTGCAGAAGAAAGATAAGACGACCAAGATGGACAG
GAGAAGAGACCGAGGAGTCAT

**AGAGGCTGCATGGTTTCTGT = forward primer (LG1a: 13,840,422 – 13,840,442)**
**CATCACTCTTGAGCTGGTCG = reverse primer (LG1a: 13,840,640 – 13,840,660)**
**198bp**

## * *irf1* _European sea bass:

>dicLab1_dna range=LG14:21268144-21272778 5'pad=0 3'pad=0 strand=-
repeatMasking=none
TAATTATATAGTAAAAATGTTTCAACATGCTCTTTGAAAAACAGGATTCT
TAGTCAACATGTTTTAACTTTTGTTTTCCTTGTGGGCATTTTTTTACAGT
GGGCATACATCATCGCCCAGCCGGGGAAAACCCATTGGACCCTATAAAAT
GCGCATAGATTTCTGTGAGTCTTCAGTGCCACTTCTGATGATCTAGATAG
GTGTCCAAGTGGATAACACAAAGACGAAGAGAACCTTCTTTGGAACAAAC
TTAAACTAATCGGACTTTTCTTGTGCATTTTCCTCTTTACGGATCTTACA
ACAAGCAAATTCTGTAAGTGATTTGATATTCATCTATCACTTCTACTATC
ATGGAATGCCTCATATTTAAACATTTTATTTTGCCGGGAGCTGCTTAGGA
TATATATATATATATATATCGTAAATCTTGAATACATGTACAGGAGGATA
CATTTATTTGTAGCTTCTAACATGAAACAAAATAATGTAACCAGCTGATG
CTTTTAATTTTTTTGGACAGTAAAGTTTATGTAATTATTGCACACCATGC
GTGCAGTAGGACTCTAATTTCTCGACAGATCAGTTCAACAGGGGACTATG
TGACTTTTTTGAACTTAGGTGTATCGCATGCATGCAGCAGTGGAGGAGGA
GGCGTGTGCTCTCTCCGCGCACTACTATAAATGTCACCCGCGGGAATAGG
ATCATGTTGCACGAAGAGTTTTGGATTGCAAATAGCAGTCCATGTGCATT
TTAAAACTTTTATGATGATAAAGCCATAAAAGTGAAGTCCGGGAATGATT
TTTGGTTCATTTTGACCTTCATAAGTACGCTTACTCCTACGCGTAAACG

CCCCCGATCCCCGGTCAGCCTACGTGCCCTGGCCGTGAACTCTTCTCACC
CTCCTCCTGCAGGCAGCAGGCAGCAGGCAGCAGTAAATCTTAACTGACGC
GGGAGTTCATTTGCTCTCACAACTGTATATCACGCACAGCATTCCTCATT
TATCTAAAGACTTACTCCTTTTCCTCTCTTCCTGCATATCTTAGGCAAAC
ATGCCTGTGTCAAGGATGAGGATGAGACCATGGCTGGAGAAGATGATCGA
TTCAAACGCCATCACGGGTCTGAATTGGGTGGATAAGGTGAGAGGCATAT
TTCAACAAATTGGATTGGTTCATGTAAAGTTACTGAAGTGAAGTCAGTAT
ATTTAATACTTACAGAAATAGATATTTAGAAATATAATTGTGAAACAACT
TATATATACATATTACTTTTAGAGTAGTTTGTTTGAAGTTGGGATAAATG
TTGGAGTTAATATTTTAAACCAACACTAGACACCAAAACAAAACCTTATT
TTGTGTGTGTGTTACTACTGTGGCTGCTGATACTGTACTATGCAAATAGT
ATTTTTACATAATTTTTGGCAATGCAATAGAAAACTGTTAACAGTGGCCG
GCTCAGTTTGAATGGCAGCCTCTTGCAATATATCACAATTTTTCTATGTC
TTTGTTCCTTACAGGATAAGACGATGTTCTCAATTCCCTGGAAGCATGCA
GCTCGACATGGCTGGGAGATGGAGAAGGACGCATGTCTGTTCAAAAAGTG
GGCCATCCACACAGGTGAGCCAGCATGAACACACACCTAAACTATGCAAA
TAATAACGCAGAATATTTTTGTTTTTATGGATGAAAAAGGTGTGAAACTA
AACAAAATGCATCCTGCAGGGAAATATGTGGAAGGTCAGACCAGTGACCC
AAAGACATGGAAAGCCAACTTCCGCTGTGCAATGAACTCACTGCCTGACA
TCGAGGAGGTGAAAGACAAGAGCATCAATAAAGGCCACCAAGCCATGCGC
GTCTTCAGGATGTTGCCTGCCACCCCAAAATCTAGAGGTGAGAAAACTGA
CAGGAAGTGAGCAAGCATTCTTAAAAAAAAAGAATTAACGTTGTTCTGCA
GCATCTTTCAGTTCCTGATGTTTTTTTTTCTTTTTCTGTTACTCCGTTTG
TTTTGCAATCACGCCATGAGCCGTTTAAGATGCATTAAGTAATCAAAATG
TCTTTCTTTCACCTGCACAGATAAACGAAACAAATCAAAGGACACAAAGC
CAAGGAAGAAGGTAAATACAGTACATTTTTTATGACTATTCTACTTTTAT
ATTGACTTAAGTGAAATTTATTGGTTACATAGTCAGAGGCCTATTCAAAT
AAT**GTCATGCTCACACTGACCCA**GATCATTTTAATGGCACTGGTGCAGTA
GCTAGACATATGACTGCAAATTTTGCTGTGGTTTTTTCCTAAATCTCACA
AAATGTTAATTTATTTCTGTCTCTGCAGAGCTCAATTGTCAAGATGGAAG
ACGACATGGACAGTCAGACTCCCATGGATG**AGTCAGCGCCAGAAGAAACT**
TTGTCCACTCAGGAGAACACAGTCGACAGCACAGTGAACACAGACCCGCA
AGGTGAGGAAGAAAATATGTATAGTTGAAGATTTTAGGTTACTGCATGTA
GGAAATGTTGCCTTGAATTTGCCCCTTAAAACTGGAGCACCAAGTTTATT
AATGCTATTGTTTGCCATCTTTGCAGAATTCACGTTTGTGGCTCCATCTG
AAGTTCCTGACTGGTCAGTTGACATCGAGCTTGAAGGCTATAGCAACAAC
AACTTCCACCAAAGATTTGAAGTTTCTCCTGACCACAGCCCTGGTACGAC
AACAACACATATTGCATACTTGCTAAATAAATGCTTTAGGACCAAGTGAA
ATTAAGTAAATTCAAACAGACATAGCAATATCACGATGCCATACTTATGT
ATTTCCTTTCTCTCCCATTCTATTTGCTGTGATCTTCCAGATTATGACTC
TGCCAACGACGACATTATTCAGGTAAGCATTGTCTTTGGTTCCTACTCTT
AACAGCATGCATTTTTCATTGGAAGGAATACCCATGTTTGTCTTATTCTG
CCTGTTCGGCTTGTGTAATAATACCCCAAATAATAAGACTGATTATTATA
GACTGTTTGGAGTTGGAAAACTCCAGTTGTGCAAACTATTATAGCCATAC
TGAGTCCAGTGTTGCTCTTTCAGATTTGCCAGCAACTGGAGAAAGACTCA
TTCTTGCTGTCAAGCAGTTTAGACTGCAAGGGGTTCCTGAGCAATGAAGC
ATGCACCAGTCCAGGAAGCCAGTGGAGTGAATCTTCCTCAGGTAAATGTT
GCTAGTTGATAGTTCAGATGTCTCAAAATTAATCTGCAACATAATGTCTT
AAGACACTCCATATTGGTAATTTAAGTGTAAAATCTAGGTGTAAATGTAG
CTATAGTTGAAATTAGCATGTAAGTCAGTATAGATCTATGTCTTAAACAC
TATTAAACATGTCTGTCTCTGCATGTAGATAATGTACTGGTGTGTAAAGG
ATATTGCCTTTATAGAGTATTCATTCCAAAATTTTTGAAGTTGAGTAAAG
TCAAGTAAAGCAGCTCTAACTATTAAAGAGCTACTCCAGTGCAATAGTAT
TTTACTTTAATAAAGTTAAACAACTCACAAGAGACAGGTTTACAAAAGAG
GGGTCAACATTAATGCAGCAGAGGCTGGGGTATCATGGCAGGTAGCATGT
TTTATTAGACTCTGTCAGCCTGATAAATGTCCACATCAAAGTCCAAGTTC
ATAACACAAACCGAGTCTTGAGACAACCCTTATTACATAACAATGACATC
GTGAAAGGTATTTGCTCTCTCATTTTTAAGACTCCCCCCTTGAAATTTGT
CTATTGTTGAGGTGTTTCATTTTTCAGGTTAGCAATACATGTTCTTCATT
ACCATCCTGACCTTGTATTTCCTCTGTTTGTTTTGCAGATGAAATAGACG
ACATGCCACCTCAGTACCTTACTTTGGGCACAGAGTTCCAAAATCCCACA

GACAACCTCTGGAACAGCTTTTGCCAACAGATCCCCTCAGTCCTGTACTG
AAAGCTTCAGGACAGGACAGTGCAGTGACACTTTGGACTTTTAAAAGGGC
TCTTCCTGCCCACAATTCTTCCCATCTGACATCTCTTCTCATCCATTATC
ATCCCTCCAGTCTGTGATGGCCCGGGTTCCAAACATTAAGCTAAACCCCC
CCCCCCTACCATATGGTCTGACGTTCTGAGACCTTTCCTCATCTCCTTAA
ACCTTTCTGTGAAGTTATGAAGAGAAGCGATGAAGTGCGCTGCCTTGTTA
GAGGAACTTACATGCTTTTCATTCTCTATGTTTTTGGCTGGTTCAAGACA
TTTTGGTGCTCAGTTTTTTGTGACAATGAGTTGCAATAGCACATAAATCT
GTTTTGCTCAGGCAGTCCATGAAGATATACTCATACTAAGCCATGAATAC
ATATGAGGTAGCCAATACTTCAAAATGCAAATTGGATTATATTTAGTGTG
CTCAACATTTTGCATCATTGTTAATGTTTAGTATCATCCCATAAAATATA
TATTTTTTTCTCCGTTATGTGCAAAAGTTATTTTACTTTAAGGGATAAAA
ATGCATCGTAAACCTCTTCTGGTGAAAGCTCTACTTCAAAGTCATACGTT
TATAAGACAATTGTAAATTACTGTATATAGTGTTTTGTTTGTGTGTTTTT
TATAAAAGAAATAAAAAATCTAAATGTGAATGAAT

**GTCATGCTCACACTGACCCA = forward primer (LG14: 21,270,348-21,270,347)**
**AGTTTCTTCTGGCGCTGACT = reverse primer (LG14: 21,270,428-21,270,447)**
**157bp**

*** *muc2*_European sea bass:**

>dicLab1_dna range=LG6:12932125-12947821 5'pad=0 3'pad=0 strand=+
repeatMasking=none
AACAGAGTTGCATATTTTTATTTGAATTGATAAATAATCTCTTTACAATG
GTAATTACTTTGTAAGAGATGTAATGATTGATAAGACAATGTGGGAAGAA
ACATTTACATCCCACATCCATTAATGTTACATATTTCTTTATCACACCAG
TGTGAAGCTTCGCCTCCTGCGAGCAGGTTGTCCAGCCGGTCTGGTGCAGT
CTGTGTCCCCACAGCCGCACTCCTCCACATGGGTGTATGTGTAGGGAACC
ACATCTCCATTCAAGCAGTGCAAGTCAACAGTGCGATTGCTGGTGCGTGT
CTCCTTACAGCAGGAGCAAGAATGCAGCACAGAAGCTGCTGCTTCAGAGT
ACCTGATGAAAGCAGAGGCTGCACTCAAAATCTGCACACATTATACCTTC
ACATGTTTATATTTATTAACTAACAGGTTTGTGACTTTTGAGAAATGAAG
ATATAGCAAAAGTTCAGTCAGGAAGAGTATTCTTCGGCATAGAGGCCTGT
AGTTAAGTTCTCTTTCCATCCTCACATTCACTCCTCTTGTGCATATTTCC
TTGCTGTCATTAACTGGGACTGTCCATTGAGAGAATAGCTGTTCTCATCT
ATCCAATAGCACAGCGATACACTTGCATTTTTATCATTTTTATCATCTTG
CTGCTGTCTTTTTAAATATTATTCTCTCTCTCTGACTTCAGTACATTCAC
GCTGCCATTGATGTGCTCTCCCTAATACCGCCCAGTCTTTGCAGATTCAT
CAACTTCATTATCTATCAGCCTATCAGCCTCAGCAGAGTCATCACCCACC
ACCAACAGTGTCTGGACTGGTGCTGCTCAGGGCATACGCCCCTCAATCAC
AAAAATCTCCCTGTAGAGTCTAAGCTTAGACTTTCAGTCAAGATTTAATC
ATCTCATGTATGTTATACTAAATACAATTATCATTCACTTGTCTCACTTG
TCCTGATTGAAATATCTTACTTACTTGGTGAAAGTGTTGCAGGATCCTTC
ACAATACGGCATTTCGATCTCCTGGTAAGACTGACAGTTCTTGTGTGTAA
TGCGGGTTTTCATGGATAGTACCTTGCAGCCCTTCTCCCTCTCCACACCT
ATTTAAAAGAAACATGCAATTAGCTTCTTTTCAATTAAGTACAGTCTACC
CAAAAGTAAAGAAAATTTCATACTCACATATTTTACAACAGCCATTTGCT
GCAGTCTGAATTGTGTCCTATGGAGGAAAATATACAACATCATAAAAAGT
TTTGTTTTAACTCTAGTTCTCAAAGACACAGTAAAGTAATTTGTTGGAGG
TGTACTTACAGGTTGGCAGTTGCTCTGCTGGAATGGCGGGCAGACAATAT
GTGAACTGACTGTTACTAAAATCTCACCGGTCTTAATACAGGTGTGATGC
TCACACTTACTCTCAGGTGGTGACCAGGTGTGTCCTAGCTGGAGAGTGAA
ATGGACGTACACACACACACACACACACACACACACACACATTAGCCGGGAT
ACACACCAACATCAAAATACTGAGGTCCTTCAGGCTTATTAAACATGAAC
ATGTGTGCCAACATAAATGATCCAACGTACCTCCAAGAGCTGCTTGGTAC
CATTAACATTGAGGACACAGTGTGTCTGTACACACTTCCCACAGCATTCA
TCAAAATTAGTTTGCACGTATTGATATCCCTGAGAGAAAGGGGGGAGTTA
AGTTGCCAAGTGCAGTTTTACAGGTTCACTCTCCCAGTCAACATATTCTG
ATGAAAACACTGCATTGTATGTACTGCATGTTGCTAGTATAACAGGTATA
ATTTCTCTTACCATGTCACAGTTTTCTTGACATTGCTGAAACTCACAAGA

364 TATCTGGAATAGGCCATTACTGGGGTCGACCTTATTTGTACAGGTACAAT
365 CCTGACAAACATTCCCAGGAACTAAAGAACCAGGCTAGTAAAATAAAAGA
366 AGAAATGTATTCATACTTCTGTTTTCAGATATACAAAACTGTAGCAGTGC
367 AGCAGAAATCTTTACATGACAAAAGTGATATATTAATAAATTATGTGACA
368 AATCATCATCACATTTCCATTTCTTATTTACCTGGTATTCCACATTCTTG
369 TGGACACAAACTCTTTTAGGCTCTAGAAAAGAGTAAAAGAGTGGAAATTA
370 CGTTAAAGTGCCATTGTTGTCATTAGGTTTGATATAAATTAAAATGAATG
371 AACTCACCACATTTATGTTCTGGACAGCATTTTCCCTCAGGGACACTGAC
372 AACTGGTATATATCCAACCGGACAGTTCATGTTGCTGTATGGGCAAGCGT
373 TGCTTTGACATTCTTTGAAGATGATAAAATGGTGATAATTTTAGATATGA
374 GCCATCCTCAAGAATTGTTATCAATGCATAGTTTTCAACAAATGTGCATC
375 AAAGCGAAGCATCTTACTGCAAACAAAGGTAGTACAGCAGCGGTCTGATG
376 GATTAGTTTGGTTGACAAGCACAAACCCTGGACCAGTGCAGTTTGTTAGA
377 GGTGGTGTTGGGCACACCTTCGGCTTGCATGTCACAGTCTTGGTGGACTC
378 CTCACAGATGCAATTTTGGCATTTGTACTCAAACATCTCATTAAACTGAA
379 AGAATAAACCACAGCAATGACTTGGTTGTCTCTTGCAAGTAAACAACACA
380 TGTCAGGATAGCAGTGAACATGAGCAGAAAACTTGGCTTACCTCACGAGG
381 AATGCCCTCAGGATCAAGACATCCTGTTGAGAAATTAGCAACTCTGTAGG
382 CACAACTGATACCATATATTCAGCTCAATGATAAGACCTGAAGATGTACA
383 CCACACTTACCACACTTATCAACACATATACCAGACTCTTTGTTGAAGAG
384 TTTCATTCCCTCAGGACAAAAGCAGCCCTCGGTAGTGTAGGTCATCGATG
385 GTTCATTAGGACTGTAATGTTGAATCGTCAAGAAAGAACAGTTAGTTTAT
386 GTTATTTCTTCATGCGTTATAAATGATGAATTCAGTATTTATTCATGCTT
387 AACTAATGCTTAATAGTGTGTACTCATTATAAAGTGTTACCAAACAGGGT
388 ATTTTAATACACTTCTTTTGCCGCTTTGCTATTAAAAATATATATTTAAT
389 GCCCCAATTAATTATGATCCTGGACTCAGGAAATGCTATAGTGCACAGAG
390 AACTGTAAAAAGAATACACTTTTTGTTGAAACTTACTTGTCTTCACAGGT
391 CGGTTGTTCTACAGGACCACATGGCTTGTAAACTTTGTTTGATGGGCAGT
392 CAATTGCTGTTAGTGAAGAAAGGTTTTTTTCAAAAAATACATACTGTCAA
393 CCTAAAGTTTATAATATGTGCAGCAGTCTGTAAAATGTGCAACAATAGTA
394 AAACAAATGAGTTCCCAGCCTATACAGTAAAATAATGAGGGTTTGGTTAC
395 CACACAGTTTGGTGTGGTTCCTCCAGTGAAGACAAACTCCAGCCTGAGCA
396 CAGGCAGCAGCATAAGTCTGCAAACTTGTGCACTCTACTACTGGGTTGGA
397 AACATGGCAGCTATCAAAAACACAACCTTGGTAGAATTTGTCAGGAGAGA
398 CAAAGGGATGGCATTCTGCAAATACACTGTGAAAGTCAAACACAAACAAA
399 AACACAAATAAAAAAGTGACTGTGTCTGAAGTGCAGTATGTCAAAGTTAT
400 GGGGATGAATATGTTACTACAGTGGATTATATAATGTCTACCTGCTCTTA
401 AGCATATCACACATGGAGTCGGGACTGCATGGAACAAAGGTTGGTGGAGG
402 TTCAGGTGGACTGGTGGGCAGTACAGATGGTATCTCGCAGTTGGGTTGGT
403 AAATGTGTTTTGCAGGCCAATAGTCAGCCATCACAGCACAATTTCCCACC
404 AGCTGGCCTCCAGGCAACATGCAGTCATCCTCCTGGTTGTTATTGCATGT
405 TCCTGACAGCAGAGTCACAAAGCAGTTAATATTACCATCTAATGTCAGTT
406 AAATTATTTAACAATATGGCCACTGGAGAGAGAGAGATGTTCTCACCACAAT
407 GGCCCTGTGTGTTTTTGCCAAAGTGTTGATATGGAAGGGTGACACTAAAG
408 CCAGTGATTCCAAATGTAATGACCACTTTTAGATGAGGGATCTCCAAAAT
409 CAAGTTAATGCCAGAATCCAAGACCTTAACACCGCCTTTTGAATAAGGTA
410 GCCTCAAACGGACTCCATCTTTTAGTGCCTATAGGGAAAAAAGTAAATAC
411 ACAATGTGACAAGAAAATATACAGTACACTATATACTAATCATACTGACA
412 GTGATAATATACTTGAAGTATATGAATTTATAATAATTGTTTCATATTCA
413 TATATACCTCTAACTGGGCTGCTCCAATAAGGTTATGATTCATGAGGGTG
414 ATAACTTGCAATCCATATGAAATAATTATTGATCGTGGACAAGACACATC
415 TTCAGTGGGATCACAATAGACATTGTCAATATAAATTTTCAAGTTATAAA
416 TTGGTAATCTCTCTTCAAACAAGACATAAGTACAGTTTCCTTGATAACTG
417 TAGTACAATCCATCAAATGTGATGTAATGAGGATCTCCCCAGCCTTCACA
418 TACACCTGAAAAGAGAGAACAAAAAAAGGTTCAACATTTAAAATGAATTT
419 AACAGTGGGGTCAGTCATTCTGGAGAGAGAAAAGTTGATAGTTGCAATGA
420 GACTCTGAACGACAGAACGGCTTACAGTCACAAGCATAATGTTGGCAGCA
421 GTGGTAGGAATCATACACAAGAACTGGTTTCTTTCCATTGGTACAAGTGA
422 TGTTCTCAAGGGGTGGACATTCATATGGAATTATTTCAATTGTATTGTTC
423 TCAATGCATCTGGCCATGGTGCAGTTGCACAAGTAGAAGGTCTCATTTTG
424 CTGATGTAAAGAGAAACAATTGGGAAAATCAGTAGTACTTTTTAGTATTT

TGCCAAAATTTCAAGAAAGCTTGTTTTCCTTATGTTTCCAGAGAATAACA
TAATATATGTAATGTGCTAGATACATAAAGATCCTTACTGTTACATTCCA
TTCAGGGCAAGTAGGTACAGAAGGTTTCGTTGTTGGGGCAGGTGTGGACC
AACAATCTTCAGTCTTGTTGCGAATTTCACAAATGTCAGAACATATCATT
GTGAGACATACACCTGACCCAATGTGTTCCTTGTCAAATATGAACTGCCC
TAAAGATGAGAAAAAAGATTAAGAGTGAATTAAACTATGGTAAACCTTAA
AAAGAAGCATATACAGTAGCTTGTCAATGTCAAAGACATAATTGATGTAA
TTCCTTTCTTACCAGGCCTATAATGCTTGCCATTAACTATACAGAAGCAT
TCTGTAGTGGCACTCGCCGTGACTACTGTTGTTGGCGTAGTTTCAGGTGT
TGTAAGGTTGCTGATAACTTGTCCAGTTGATGGTTGCGTTGGGGGAAGCT
CTGTGGGACCAGTTTCAACTTCTACTGATGTTGAAGTTGTGGAAACAGTG
GTTGGCCCTTCAGTTGTAGTAGTAGGTTCAGTTGGTGGGGCTGTAGGTTT
TGTGGTTGTGGTTGGTGCTTCAGTTGTTGTAGTAGGTTCAGTTGGTGGGG
TTGTAGGTTTTGTAGTTGTAGTTGGTGCTTCAGTTGTAGTAGAAGGTTCA
GGTGGTGCAGTTGTAGATTGTGTGGTTGTGGTTGGTGCTTCAGTTGTAGA
AGTAGGTTTAGGTGGTAGTGTTATAGATTGTGTGGTTGTGGTTGGTGCTT
CAGTTGTAGTAGTAGGTTCAGGTGGTGCTGTTGTAGATTGTGTGGTTGTG
GTTGGTGCTTCAGTTGTGGTAGTAGGTTTAGGGGGTGCTGTTGTAGATTG
TGTGGTTGTGGTTGGTGCTTCAGTTGTAGTAGTAGGTTTAGGTGGTGCTG
TTGTAGACTCTGTGGTTGTGGTTGGTGCTTCAGTTGTAGTAGTAGGTTCA
GGTGGTGGTGTAGATTGTGTGGTTGTGGTTGGTGCTTCAGTTGTAGTAGT
AAGTTCAGGTGGTGCTGTTGTAGATTGTGTGGTTGTGGTTGGTGCTTCAG
TTGTAGTAGTAGGTTCAGGTGGTGGTGCTGTTGTAGATCGTGTGGTTGTG
GTTGGTGCTTCAGTTGTAGTAGTAGGTTCAGGTGGTGCTGTTGTAGACTT
TGTGGTTGTGGTTGGTGCTTCAGTTGTAGTAGTAGGTTCAGGTGGTGGTG
CTGTTGTAGATTGTGTGGTTGTGGTTGGTGCTTCAGTTGTAGTAGTAGGT
TTAGGTGGTGGTGTAGATTGTGTGGTTGTGGTTGGTGCTTCAGTTGTAGT
AGTAGGTTCAGGTGTTGGTGTAGACCTTGTAGTTGAAGTTGGTGCTTCAG
TTGTAGTAATAATTTCAGGTGGTGGTGGTGTAGTCGATGGGGTTGAATGG
GTTGTGGTTGTACATATGATTACCTCACAACATACTCTGATCTTATAGTT
GAAGCACTTTCGTGGAGGTCTCAACTGGTCCTCTTGTCTACATATTAAAC
CATATCCCAAATCACAAGTAACAACTTGGCCAGTTTGACTCACAAAGTCA
TCGAAGTGCTGGTCAGAATAGTCTGCAGCTCTACAATCGATTTCTGTGCG
ATTTTTGCATATTTCTTTACCACTATTTTCAATGTTCTCATATGTTTCCC
AGTCACTTTTGTCTTTTTTTGGGTTATCAACATCATACCACTCTGACCAC
TCACATGTTTCAATACATGGAGTGGTAGGAGCTGTTGTAGACTCTGTGGT
TGTGGTTGGTGCTTCAGTTGTAGTAGTAGGTTCAGGTGGTGGTGCTGTTG
TAGATTGTGTGGTTGTGGTTGGTGCTTCAGTTGTAGTAGTAGGTTCAGGT
GGTGGTGCTGTTGTAGACTCTGTGGTTGTGGTTGGTGCTTCAGTTGTAGT
AGTAGGTTCAGGTGGTGGTGCTGTTGTAGATTGTGTGGTTGTGGTTGGTG
CTTCAGTTGTAGTAGTAGGTTCAGGTGTTGGTGTAGACCTTGTAGTTGAA
GTTGGTGCTTCAGTTGTAGTAGTAGGTTCAGGTGGTGGTGGTGTTGTAGG
ACAGGGTTCATTTCCATGTATCACTGTTGTATTAATGCAGATTGCATAGT
AGCACATTCCCAAGTTGTCAGTCACATTGTAAATAACTTCATTTTCCTCA
TATCTTGTCCCATTATAAAAGCAACCATCACATTCCTCCACACACATCTG
GGTCACCTCATCAAATATGGGCTTATTTATCGGACATCGAGGGTAACAGC
CTGTAAAAACCACAGCATAAAACATTGTTATATTACCATAAAATAATTAT
TTACATTGTATTTCAATCTTTGACACTGAAACTGACCAGGTTGTCTCTAC
CTTCATTTTTTGTCACATATAGTAAATCGGATTCATTCAACATTTAAGAT
TTTAAGCAGCACATGAATCAGAGGTGAGGTGAGACATTGTGAGTTAACCT
CTTACCTTCCAGGTTGGGTATAGGGTTGCTACAAATTCCATCTGGATTTA
AACAGGTCTTATAACAAGGTGTGTGACAAGGGCTGTAGTGCCAGTTGCAT
TCATCTGGGTTGTTGTAGTAATCACAGAAGATAGCTAGGAGGAAATGATG
AAAGGTATGTTATGTTTAAAATAAAGGAAATATTGAATAAAATAAGTAGA
TTAATCTCAATCTCAAACTTAAATTTCATCTTCTATATTCTTACATTATA
TTTATATCAGGGCTCAAGTTTACATTGAGTTTAAGCTAAATATGGGAAGT
TATCGCTCACTACAAATGGCAGAAAACTATCATAGATAATGATTAAGGGA
AAGGCTTAGGTACTTTTAAGTAACAACCTTCAAGACAATTAAAAAATCAA
TTCAATACCAAATTACAGCTGATTCAAGTATGTATTGAACTACAGGAATT
ATGTAAAGCATTGCAGTCTTAAAACTACACTTTTCACTAGGCATGCACCC
ATAGTCTCAGTAACATATATCAGCTTTAATTACATTAGGTGTCCATTCAA

486 TTAAATAAATAAACAAACAACAAATATATAAATAAATAAGTAGGGTTGAT
487 TATTGTATTTCAGTACAGAAAAGAGAAAATTAATTATTTTCCTATATACA
488 TCCCACACATCAATCTTTAAATGCTGGTATCATAAGATTTTTGGGATCTA
489 AAAACAGGATCTTGGTCAGAAAAGGCTGAAGAAAGAACCCAGTTTAATAT
490 GAAGGTAAAATTGTTGTTTACTTACGACAGATTTCCGGAGTTCTCCATGC
491 AACGCAGGAACCAGCCTCATTACAAGCTTGAGCATAAGCTGCAACTGCTG
492 AGCAGAAGCACTCACAGTCTCCTCCGGCGTCGCAGGCACATGAGTCTTTC
493 ACACAGTTGTCATAATATGGAAGGGGATTTACCTGTTATCAAAATAATAT
494 ATATTTTTTAAATGTGTTTTTCAACCATGAACGACTTTGAATAACAACAA
495 ATTAGAGACAAATCTACCATTACCTTATTATGGCAGTCTTTGAATGTTTT
496 TCCAGTTATAATGCTGCACATCATTTTTGCCCAGTGGTGTCTGTTGGGCG
497 TTGCTCCACAAGGGTCAAAGTTCATTCCTGCATCTGGGCAGGTGCTCGAC
498 ATTTTCCAACTATTTGCAAATTCTAACACATTGCTAACTATCAGCTGACC
499 CTGGGTGGTAAAGTCATTCTGTCCATCGCCATCAAAATCTCCACACAGAC
500 CACATACCTCTCCCTGAACGGCCAGAACAAAAGAACAATATTTCATATCA
501 GTTCTCTTTGGCATTATAATGAATACACATTTCTAAGGTATATATCTTTG
502 TATGTTTATAAACAGAATATCCCATTGTAATCACGAATATAAGTGGCTCA
503 CAGACTTTTTCAGTAATGTTAATATACTGTACATCAAGATGAATACAGAT
504 GAATAGACCAAATACTGTCACTCACGCTGTGCTGTGGTTCCAGGAGGACG
505 CGAACAGTTGTTTTGCGATCCCACATCACTGCCAGACCAATGGCAGATTC
506 TATCACCAGATACAAGCCGACATGCCTTATTTTGTACTGAATCTGTGTGC
507 CATGTCCCAGGTCCAGCTCTTCATATTTACCCTTCGCTAGTTTGATTTCC
508 ATTTTCTGTAATGTTAAGGAGATTTCTGTTGCTTTCATTGTGATGTAAAA
509 AAGTTTGATGTATGACAAAGGATTCTAGGCACAAAATAGATGTTGGTATG
510 AGGTCTTACCCCCAGTTGGATTCGGACAATTTTGGAGCATGTTGTGCCTG
511 TAGTTCCACACGGTACATTTTCTGTGATGACTCCGAAGTTGTCCTGAACT
512 GTTTTATTGCCACATTTGTTCTTGAAAGAGAGATATTTTGCTAGTTTTAT
513 ACATAGTAAACAGAGACACTTGATCAAAATAATCCATAATACTTTAAGAG
514 TCTTAAAGAAAAGCTCAGTAACACATAAATTAAAAGTGCAACTGATCCCC
515 AGAGACATGACATAATGTAGTTAGCTGCATCAAATATTGATTCTTTCAAG
516 TCTGATGTTCAAGATTAGCAATAAGTTTCAATTTTATAAATTTCTTGTCT
517 ATGAATTTCTGAGATTAAGCCTGAGACAACGTAAAAGTGAAGCATAACTG
518 TTGACTCCGTCCAAAATCACTGCCTGTTTACTGTATAGTGCACTACAGTA
519 TATTTACTGTCTGCCATTTTATTTGAGTGTCTGAATTCTGAATGTGTAAA
520 TTTATCCAGTAGATAGGACAAATGATTGTGTCTATAGTATCTTCACTACT
521 TTCTACTGACAATCCATCACTGCATATATAGACAACTCAAGAGCTGTCAG
522 TTACGATGTGAAGATTGATTTTGAAGTTAGAATACATTTGGATGATTTTG
523 ACGTGTATGTTTACCTTGACAGCAACATAGCCACACTTTCCTTGAAACCC
524 ATATGTTTTCTTATCAAATGTATTGTAGTGACCACTCCCATAAATAATGC
525 AAGTTCCTGGACATTTTTTCTCTGTGCACTCCCAGTTTCCACTTTTGCAG
526 GTACTAGATGTATCAAAAAAGGTTAAAATTATTAAAGTAAGTGAGTAAAA
527 CATGCAGCCAAATATGATAATATGTGTTCACTATGCTATATGATATGTCT
528 GATTAAAAACAAGGTCGTACCAGGTGTTGCATTGGTTAGGGATTTGTGAT
529 CCAGAGGCATAAATATGCCCATTATGCTGACATGGACATTCATTTTCTTT
530 CACACAGGCACCTTTGCCATCATAAAGGAGGCCACTTGGACACTGACATC
531 CAGATTCACATTCTGTGGAGTCCTGGGAAGATAGTAATATAACGGTGACA
532 GAAAACGACAAAACATTACTGATACTGTCAAAAATTTCTTTTCAAAACTT
533 GTTTCTAGAAACTACTGTAGAAACAAGTGTAACTATACACTGTGTGTTGC
534 ATGTTCAATGTAACATACTGCAGGAAATAAATATTAGATCCAGTTATTTG
535 AGACACAGTGGCAATAATCGCTCACACAATCATCGTTGTCCAGATTTAAA
536 CAAGTTCGAGCACACTGCAGTCCAAGTTCTCCTGTGGTCGCAGTGGAGCA
537 GTTGAAGTATACTTGTGGAGATGGGCATACTGTAACGGAAGTAGAAACTT
538 ATTAAATATTCATTGTATCAACCAAATGCTTAAAGAAAAAAACATAAAAC
539 AAAAAAACTTACATGATGAACGGGCTCTCCAAGAACGGCAATGAAGTATT
540 CCATTAGTGCACACGCTAGAAAAACATACTAAAGTTAAAGCAACACCAAC
541 AACGTAGTTTATGTAAATCTTATACACTGCAGACAAAATTATTCTAAATC
542 CTTAGTCTAAGATAATTAATTCCATTGTCCCCAAATTAGAAAAAACTGAC
543 TCACCAGTGCTCGTCTTTGATGCTGATAGACTTTCCGGGTTTAATGTAGA
544 CTTCGTTATGGTAGCAGGAACATTTTTCCACGGGGACGCAGATGCCGTTT
545 TCGTTTAGGTAGAGACCCTCAGAGCAGGAGCAGCCGTCCACAGGCAAGAA
546 GTCAGAAGTGCAGCTCTGCTGCTTTGAGCCCAGTGATCTGCAAGTCAACT

547 GGCATCTCTGATGCTTGTATGAGAAGACCTGAGTTGCTGGGCAGCTGTTT
548 GTGTATTTATCTGTTTTGTGCATAATGTTACAATGGTTACTTAATACTCA
549 AACAATGAAAAGGAAGATGATCATAAAACTCCAGAAATCGTTACTCAACT
550 TAACCAATGAATGAAAGAAATGCTTACCGCACACATTCTCTCTCCAGCCT
551 GTCAAAAACAGTCCCTTTGATGCACAAGCACGTGCGTAGGAGGAGAAGAC
552 GGCACACAGGCAGGCCTCACTCTTGTCACAGTTACAGCTGGCATAAGTAC
553 ATCGCTGCAGAAGACAGCAAACACTTTTAGAGAATTACAGTCCCATCACA
554 AACTAATTCAGTTAAAGACAAACCGGAAGGTTTTGCTGTACCTTATGGTA
555 CATCTCAGGATCCACCACTGAATGGCATTGAGCAAAGGTGCTGTCTGGAC
556 TGACCAGTAAGGCACACCAGTGTTTGGCGTACCACTCTGAGAGGGGAGAA
557 TCAACATTTTTATTACAAATTCTATTGAGCTGTCTAATTATATGTATCAT
558 ACATTCAACAAACAAGTAAGTTTAAAGAAGAGAATCATTTTAAGACAAAA
559 AAAACGGTGTAATAATCAACCACTGCTGTTTACCATTTTCCAAACTGAGG
560 GAACAGGGGTCATCAAGTCTTTCCTCTCTGTCTCTGCATGCGCGACTTGC
561 TTTCCACGAATTACAGAAAGTTGCTGATGTACCCTCCACAATCCCCTGAG
562 GAGTCTTCATGTCATCAGACAAGATCATGTTGTAGTTCCCACATAAACCT
563 GAAAAAAAACAAATATTTTATAAATAAATCTTTATATTCAAATTTTTTTT
564 CAGTACTTAAACACAGACAACCAACAATTGTCTGAAACCACAAAGAAAAA
565 AATGGAAGGTGTGAAATACCGTAAAACAAATTACAAAATGGATATGGTAA
566 ATCATAGGATTAGGAATTTGGTTAATGTATTTGAATTCAGATTAAATAAG
567 AAAACATATATTATACACTAAGAATTGGCGAACTGCAGTGTGGTAAGATA
568 GGGCCGGCTCATTCTTAAGTAACAAAAACACAACAATTCTTATTTTTGGG
569 TGATTGTACATTAATGAAAACACAACTATTAACATTATATTCCATTTCTG
570 CCAAAATATAATCTTAAATATTACACAATTGACCTTTAAATAAAGAGTAT
571 AACTAAATACATGTGTGTCCACATGTCCAGCACTACTTGGCCAACAAGAA
572 TTGTGAGCGGTTCATTGACAGTATGATCACCAACAGCTGGCTTCATGGAA
573 GCTTTGATAATTTTTTCCCATAAATGTGTGTATTTGTGACAGCTGTGTAT
574 GGCAAATATTCTTTCATAATACCCATACACTCATATATCAACGTTACTGC
575 TGTCATCCTCATCAGACTGTAGTAGGTAATTCCTACAGCAACTAATAATT
576 CATTGTAAATTCACTGTATTCTTTGATGATGCCAATGCTTCATGTTAAGA
577 CAAATGTAAAACTTACCACGTGTCTTTGCTCTGTAGTTCTGATCCAGACT
578 GACATAGACTTGCATGATAGGCACATGCTGGATCTGAATCTGCAAACCAA
579 AACTGGTCTGGAGCATGACGTGAAAGGATGACGCGTGGAAGATGTTGATG
580 TCACCTTTAAATAAAGAGAAATACAAAAACAGTCAGGTCATCTGCTGACG
581 TCAATCTATAAATGAAAGATGATATATTTAATTCAGAGTTCCTTATAGTC
582 TGACCCACCTGAGTGGTATGGCAAAGTGATGGTCTGCATATTCTGCTTTA
583 CTGTGCCATCAGAAGTGAACATTAACACCTGAATGAAAACAGAATTGATC
584 ATTAGAGTCTGGCAGGGATCAGTGTGAATTGATTAATGACTATAATCTTC
585 TTAACTCACATTGTTTCTGTCATTGTTCAGCACGATTTTAAGGCTCTTTA
586 GACAAGTGTCAAACTTCTGGTTTACACATGGAACCAACTGAACCAGGATG
587 GTAAACTTTGGACTTGCATCATCCTGAAATACACATATTGTGAGTGCAAT
588 TAGATAAATAACACATAACAAATTATATATATTTATATTTAACTGTACAA
589 AGCTATGTCTCACACACCTTGCTTTGCACTTTGGCTAGGGTGTAGTAACA
590 CTCTCCGTGGAAGGTGTAGGTTTTCCCATCAAAGGTCGTCACATGTGAAC
591 CCTCTTCAACTGCACATGTGGCAGGTGTCTGAAGGCTCTTACAAGCCCAT
592 CTGCCCTCAAAACATGTACTGAAACAACCCAAGTCATTTTAGTTAACGAA
593 ATATGAGATTAAAAAAATTCAATATTATGATGACATTATGAGATGTTAGA
594 CATACCATTCCTCTCTGTCCAATCGGTAAACCTCTCCGGAGTTATAGATT
595 TTGTTGTGTTTGCACTGACATTCAGATTGAGCAATACACCCTCTCATTGA
596 AATATCATCAAACACAGTGCCTGAAGGAGATATGTGACACGAACATTTAC
597 AAACTAGGGAGATGAAATCTGGAAGCATCTTTAAGGTTAATATAAAGTAA
598 AACAGGAGACTAAAATTATAGTATGTACATGTAAACAGCACTAACCAGGA
599 GGACAGAAGCAGCCGTCCATTTTGTGATCCTCACACAGGGAACTTGTGTC
600 TTGATTTGTGCATGTATCCATGCAAGGGGAACCGCTCTCTTCATAAATCA
601 TGTTGAATGGGCACTGTTTAGCTGTAAAGATATGGTACAATTGAAATAAA
602 TGGAGATAGGTTTAAATGTTTGTGAATTATTCAATAGTATATAAGGGGTG
603 GAAGAAACTCATTTGACTTTTCAGCAGCAAGTGCATCAAAATAGATTGTT
604 TTGTGTACTTATTTACTTGCACTTTTGTAATTTAACTGTATTTTGCTAC
605 ATTTTAAATCACTTACCCAGAACTTATTTTTCACCTCTTTGTGAGCGCCA
606 AAAACTCGAAAATAATATGCTATTGTATAACATACAAATGCTTTTAGAAT
607 AAAAGTAATATTCTCATTCTAGAGCTAAATTCTATAAGCTAATGTTCAGA

AAAGGTAAGTAGTACTTTTACAGGACCATTCATTTTTCACTGGGCTGATT
TCATCATAAAATAAAGATGATAACAAAACACAAAACATTACCACAGAACT
GAGGTGTCCTCCAGTTGGGAGGCTGCCCTCCCGCATGGGAACATTGTCGA
GAGAACTCCGACAGTGTGCTGCAGACACAGAAGTCATTAGTACTGTTGGT
GCAGCCACACATGTCCTGCACACAGGCCTGGATGTAAGGTTTAGGGTCAA
TCAGTTGGGTGCAGGAGCTCCAGGACGCCGAACGCAGCATCTGGTCACAG
GTGGTTTGCTAAAAGTGAAAAAAAGGCAGATAATAGAGACAAAGTTACTC
TGCTGCAAGTGCAAATGTTTATCTTATTAAATACCACTATTGCTGATACT
GTATTTATAATGAACTCACAAACTCCTTGCAGGAAACCAGCACATCATCC
ACTGATTCACCTTCCTCTTCAAAGGGGTCCTCACAATCATCGTTTGGACG
ATGGACTTTGTGTCTGTTGCCAAACTCAATGGGGCTCATTTTGCGACCTG
CAATTGTAATGTAATTATAGTGTCACAAATGTCACAGATACAGTGTATGA
TTGTTAAAATGCATTAATACATAAAGTGAATAACCATATATACCATTATG
AATTAACTCATTGTAGACAGGAACACCATTAAAGTCTCCACAAAGTCCAC
AAGTACGATTAGCATATTCAGGA**TCCATTTCCACCTACAACACAG**CAAGA
GGTTATCACAACCTTTTTCACATTATAATATTATATTATATTATAGTGTA
CTATTAAAAGCGATAAAATAAATAAAATATTTATAAGCTTTTACCATGAC
TGCATCGTCACCATTCCACATGACAGTGATGCCAACTTTGGATTGGAGCT
TGATGTAAACTGCATTTTTTTCCACTTG**TACGCCTGCGTTGTAGTATG**GC
ATTTTGATTCTGTAGGAAAAATGTTTTATTATAATGCACGAATAGAAAAG
AGAAGTTCAGTTGTTTTGCCTTTGCGGGCTGTACGGCTTGTTACTCACGG
AAGGCCGTTCACAGTGATCAGGCCCGTGGTGAGGTGGAATGAAAGGTCGT
TGATGGTGACCACCACATAACTAACTGTAGGGTCTCCATCGCTCTCCTTC
CTCTTCATGTGCACTGAAAACTCCTGGTAGGTCTCGTGGCAGTCGGAGGC
CAGGTTGTATTCACACATACCAGGGAACTGATACACATCCCCATCAAATG
TCTTGAAGTGCTCTCTGCCCCATGTGCTGCAGATGCTGCTGACATGGTTG
TGAACTGGGGGAGACATAAAGCATAGAAACCAGTTTGAGTTACTAAATGC
TCTACATAAGTGATATCAGAGACAGTGCTAGACATTATTGATGTATGTAT
ACATAATAATAATAATAATAATAATACATTTTATTTATAAGCGCTTTTCT
AAATACTCAAAGACACTTTACATAAAAACATAAAAAGCAGAATAATCAAT
TCACACTATCCTGAAAAGAGTTCCACAGTGAAACAGTCTTACCTCGTCTG
CCTTCAACATCGATGACAATTGCGAGAGACAGAGCAAGAAAACACAACGA
CACACATCTCCACCTCATTGTTGCCTCCGTGGGCTTTCTGTCACCAG

**TCCATTTCCACCTACAACACAG = forward primer (LG6:12,932,125 - 12,946,900)**
**TACGCCTGCGTTGTAGTATG = reverse primer (LG6: 12,932,332 – 12,932,352)**
**183bp**

### * *tnf-alpha*_European sea bass:

>dicLab1_dna range=LG16:3707191-3709040 5'pad=0 3'pad=0 strand=+
repeatMasking=none
ACAAAGAGAGAAGTATCACCACAGAGCACTGGAAGCACCTGAAGACACAG
CATACAGCCAAGTGTTCAGCTGGCAGATATTTCTAAACAGCAAGCAGTAA
AGACTGACATCCAAGTTGTGCACAGTATGGTGGCGTACACAACTGCACCA
GGTGATGTGGAGATCGGTCTTGAGGAGAGGACAGTGGTTATGGTAGAAAA
GAAGTC**CTCAACACAGCGGATATGGA**CGGTGTCCGGGGCCATTCTCATTG
TGGCCCTGTGTTTTGGAGGTGTCCTGCTGTTTGCTTGGCACTGGAGTGGA
AAGCCAGAAATTATGGTAAGGAGTAGCTTCAATAAGAAATCTTTTAAAAC
CCAATTTTTCTTGTAAGAAATGACATTTCTGTGTTTATATGGCTAATCTG
TTTTTTGCCCCCTCCACACAGACGCAATCAGGCCAAACCGAAGCACTAAT
CGAGAAGGACACT**GCTGAGAAAACAGGTGATTA**TATGCGTGGTCGCTCAT
TTACAAAAAATACTGAGCGGTTTTGTGAGATATATTCTTTGATATAGTCT
TTGTGGATTATTTGAGACAAGCTCACATTTGTCTTCCTCTTCTCAGATCC
CCACTACACACTGAAGCGCATCAGCAGCAAAGCCAAAGCAGCCATCCATT
TAGAAGGTGAGTCATAGCTTACCTTGATTTGGTCTCCAGCAAACAATGGG
CTCATGATCTTGGCTTTCAAAGGAGCCTTTCAGACTCTTTCTTCTACCTG
TGTGATGCTTTTTTTGTTTTAGAAGTATATTAGTCAGGAGCACTATAAGG
AAGTTGTTTCATAGGTTATTCATTCAACTCTCTCCTTTTAACATAGCTGT
TAATCATTTACCTCTGTGGAGAGAAGAATCAAGGTGTTTCAAGGCTTTAA
CAGCGCTTCTTCTTGCTCTTTTTCCAGGTAGCTACGACGACGAGAGTTTG

ACTGCCAAGCTGGAGTGGAAGGACGGTCAAGGCCAAGCGTTCGCTCAGGG
CGGCTTCCGACTGGCGAACAACCAGATTGTCATCCCACAAACCGGCCTCT
ACTTCGTCTACAGCCAGGCGTCGTTCAGAGTCTCCTGCGACGATGGTGAA
GAGGAAAGTGCGGGAAAACGCCTCACACCTCTCAGCCACAGGATCTGGAG
CTACTCAGACTCCATAGGCAACAAAGCCTCTCTGATGAGCGCGGTGAGAT
CAGCGTGCCAAAACACCGCTCAGGAGGACAGCTACAGAAGCGGACAGGGC
TGGTACAACGCCATTTATCTAGGCGCAGTGTTTCAGCTTAATAGAGGAGA
CAAACTGTGGACAGAAACTAACCAGCCATCACAGCTGGAGACCGACGAGG
GCAAGACTTTCTTTGGTGTGTTTGCACTTTAAAATGACTCTTTGCTGTTA
AACTAAAAGAGAAGATACACAGCTCTGCACAGTGCCATACGTTTTTTTGG
TTTTTTCGGTTATTCATCTTTATGGTGATGTAGATATTTTTAAATCTCAA
TGGAGATAAAGCTTGTAGCTGAACAGGCAGTGCTGATTTTTAAAAATCCT
ATAAACTGTAACAGTTTGTAACATTATTTCTATTTTAGGCACTTTTTGTA
CATTATTTATGATGACCTGAGATTGTAGTAGGCTAGACTTTCTCTGCTGT
ATCAGATGACGACACAGCTCTTCACCGGAGAGTTTAGGTTCAAGGATTAT
GAGAATTATGTACAGAACTATTATCATGTACTATGACATCCTGTATGTAT
TTATTTGTATTTATTTATATTTAAATTGTTGGGATTAGGAGAAGATTATA
TTTATATTTACTTCATGTGCACATAAACTTAAAATAAAAAAAGATAAAAA

**CTCAACACAGCGGATATGGA = forward primer (LG16:3,707,397 - 3,707,417)**
**GCTGAGAAAACAGGTGATTA = reverse primer (LG16:3,707,674 - 3,707,694)**
**257bp**

*** *r18S*_European sea bass:**

TGGTTCCTTTGATCGCTCTTAACGTTACTTGGATAACTGTGGCAATTCTAG
AGCTAATACATGCCAACGAGCTGCTGACCTCCGGGGATGCGTGCATTTATC
AGACCCAAAACCCATGCGGGGTGCCTCTCGGGGTGCCCCGG**CCGCTTTGGT**
**GACTCTAGATAACC**TCGAGCGATCGCTGGCCCTCGTGGCGGCGACGTCTCA
TTCGAATGTCTGC**CCTATCAACTTTCGATGGTACTTTCTG**AGCCTACCATG
GGACCACGGGTAACGGGGAATCAGG GTTCGATTCCGGAGA

**CCGCTTTGGTGACTCTAGATAACC = forward primer**
**CCTATCAACTTTCGATGGTACTTTCTG = reverse primer**

>dicLab1_rmsk_DLA_family-267 range=UN:96922353-96924342 5'pad=0 3'pad=0
strand=- repeatMasking=none
GGAAGGCAGTGCCTTCCTCGGGTGGTCGAGGTTGCACAGGGGGTTTTGAA
ATTTTTTTTTTTTTTGAAAATTTTTTCTAAGTATTTAACCGAGGAAGGCA
GCGCCTTCCTCGGGTGGAGACGCCAAGTCAGAAATTTTTTCTAAGTATTT
AACCGAGGAAGGCAGCGCCCTCCTCGGGTGGAGACGCCAAGTCAGAAATT
TTTCTAAGTATTTAACCGAGGAAGGCAGTGCCTTCCTCGGGTGGAGACGC
CAAGTCAGAAATTTTTCTAAGTATTTAACCGAGGAAGGCAGTGCCTTCCT
CGGGTGGTCGAGGTTGCACAGGGGTTTTGAAATTTTTTTTTTTTTTTTTG
AAATTTTTCTAAGTATTTAACCGAGGAAGGCGCTGCCTTCCTCGGTTAAA
TAGTCGCCAAGTCAGAAATTTTTCTAAGTATTTAACCGAGGAAGGCAGTG
CCTTCCTCGGGTGGTCGAGGTTGCACAGGGTTTTGCAGGGCACCATCGGA
ACGATTTCAGAGACCCAGTTTTTGAAAACAGGGGCCTCCAGAGCAGAAGG
CCGACTCTACCTGAGTCTTGTATCTGGGCCCGCGGTAGTGCATTCGGGCA
CTTTTGATGGGTTTCCCCCCTTGTCAGCTCCGGATTTTAACACTTTTACG
ATTCTGACCGACCGGCATCCGTGACCCACCATCGGAGGGCCCCCGCCGGC
CAGCCTGAAAGGCTGGCGTTCGGGCGGACGGTTCCTTCGGCCTGTGGGTT
CGCCTCTATGGCCGGGAGGGCCAATGCGTGGAGGGAAGGGCGGCACTGAA
CCCCCCCCCACCCCCGCGCTCCTCCCGGGCCGACCGCTTGGTAGCGAGAC
CGAGACGCCCCGTCTGCCCCAGTTGTCCTCCCACGGAGCCCGTCGCGGGG
TCGAGGCTCACCCGAGCCAGCCCGCTTACGCGGCGGTGCCTCCGGCAAGC
ACGATGTTTCTCAAACCCTGCCAACACTCAATGAGTTGGGAGTAAGAAGG
AGAGATGTCCCCACAAGGACCGTCTCTCGCCACTCCCCGCGTTTACGCTC
ACAAAAGCTTCGCCCCGCCGACCTCCCGGCCGGCGGTGGCCACAGCACGT

CCCTCGGCGCACGGGTTTTCGGTCGGGCGTCCCCGAGCCGATCCCCGCTG
TCCGCACAGGGCTACCTGGTTGATCCTGCCAGTAGCATATGCTTGTCTCA
AAGATTAAGCCATGCAAGTCTAAGTACACACGGCCGGTACAGTGAAACTG
CGAATGGCTCATTAAATCAGTTATGGTTCCTTTGATCGCTCTAACGTTAC
TTGGATAACTGTGGCAATTCTAGAGCTAATACATGCCAACGAGCGCTGAC
CTCCGGGGATGCGTGCATTTATCAGACCCAAAACCCATGCGGGGTGCCTC
TCGGGGTGCCCCGG**CCGCTTTGGTGACTCTAGATAACCT**CGAGCCGATCG
CTGGCCCTCGTGGCGGCGACGTCTCATTCGAATGTCTGC**CCTATCAACTT**
**TCGATGGTACTTTCTGTG**CCTACCATGGTGACCACGGGTAACGGGGAATC
AGGGTTCGATTCCGGAGAGGGAGCCTGAGAAACGGCTACCACATCCAAGG
AAGGCAGCAGGCGCGCAAATTACCCACTCCCGACTCGGGGAGGTAGTGAC
GAAAAATAACAATACAGGACTCTTTCGAGGCCCTGTAATTGGAATGAGTA
CACTTTAAATCCTTTAACGAGGATCAATTGGAGGGCAAGTCTGGTGCCAG
CAGCCGCGGTAATTCCAGCTCCAATAGCGTATCTTAAAGTTGCTGCAGTT
AAAAAGCTCGTAGTTGGATCTCGGGATCGAGCTGACGGTCCGCCGCGAGG
CGAGCTACCGTCTGTCCCAGCCCCTGCCTCTCGGCGCCCCCTCGATGCTC
TTAGCTGAGTGTCCCGCGGGGTCCGAAGCGTTTACTTGAAAAAATTAGAG
TGTCAAAGCAGGCCCGGTCGCCTGAATACCGCAGCTAGGA

**CCGCTTTGGTGACTCTAGATAACCT = forward primer (UN:96,922,353 - 96,922,380)**
**CCTATCAACTTTCGATGGTACTTTCTGTG = reverse primer (UN:96,922,440 – 96,922,469)**
**50bp**

*** *dicer1*_European sea bass:**

>dicLab1_dna range=LG12:1238921-1265767 5'pad=0 3'pad=0 strand=-
repeatMasking=none
CACCGCTCTAGTTATTTCCGTTACGGTTACTTTATATGTTAATTTGAGAC
AAGGCTAGCTTGTAAAACCATGTTGCATTGTTATCTTGTGTTGTTTTATT
GACAGTGAATCATCAAACAAAACACAACACGCTGACTGACAGTGAGATGT
TTGACTCAGGACTGATGATTCAAATAGTTGACAGCAGGCAGCTCTAGTTA
TTTGCACTTCATTAAGTGTCATAGGTGGACTGAGACACACTGGTCACTGT
TTACAATAATATTCTCCTCCCTCCTGTCATTCTCTGCTTTCTCTCACACA
GGTGCTGGTCATGACTTGCCACATATTCCTGCACATTCTGAGGAATAAAA
TCTTACAGTTGTCTAAAATCAACCTGGTGGTTTTTGATGATTGTCACCTG
GCCATCACGGACCACCCCTACTGTGAGATAATGAAGGTGAGACTCTGGTT
GCAAAGTAACATTCATGTATTTTTTCACACGTTTGTCATCTAAGGGAACT
GTGAATGACCTGGCTCTCCTGTCTGTCTGTGCAGCTGTTTGAGGGCTGCT
TGTGCAGTCCTCGTATCCTGGGTCTCACAGCTTCCATTCTGAATGGGAAG
TGTGACCCGTCAGAACTGGAGCAAAAGATCCAGAATCTGGAGAGAATCCT
GAAGAGCAACGCTGAAACTGCCACTGACCTTGTGGTCCTGGACAGGTACT
ACAAGCCATTATATATAAGCTGTACCACCCAGGGCTAAATAACTACTCTC
TGGCTACATATGTCCAGAATTGTGTTAGTTACAACTAAACATTGAAGCAA
GTAATTTCACTGATTCAGACAGATGCTGAAAAAATAGTATGTAAAACTCA
ACAGACTGACAGAGCAATAACAAAGACTTCTAGTGTAACCTGCGCACTGT
GTGTTATTTCAGATACGCCTCTCAGCCAAGAGAAGTGGTGCTGGACTGCG
GCCCCTACATGGATAAGAGCGGCCTCTCCTCCCGACTACAGGCAGAACTG
GACGAAGCTCTCCACTTCCTGCATGACTGCAACATATCTGTTGCCAGAGA
GGACCGTGATCCCACATTCATCTCCAAACAGGTTGGGGCTAGAAAGATTT
TCTTGGTTGATTCAAAGTGTGTGTGTGTGTGTGTGTGTGTGTGACAAACA
GGAATTTTCTTTCGGCGTGTACTTTTTACTGCATAGAAATTGAACAACAG
GTGTTTGATAAAAAATCTTTCTTATTACTGATTTTATTTTTATTTATTGTG
ATTTGATAAATTTGGTAAATAATGGTGGTGCTTGGAGATGTAATGTGAA
AGGAGTCTTTTGTAGTTACAAACCCACAGAGAATTATCACCAGCTCTAGA
GCATATTAGCTGATTGTAGTTAATTCTTTTCCACCCTGCAAACTCGGCTC
TCATCAGACTTGTTTCAGGCAGCAGCTGGTTTCAGTGAAAAAGCTCCGAA
CCTGCTGTCCACACTCTCTCTGTCCTCTCCAGGTCCTGAGTGACTGCTGG
GCCGTGCTGCAGGTGCTGGGACCCTGGTGCGCGGACAAGGCGGCGGGCAT
CATGGTGCGGGAGCTCCAGAAATACATCAAACACGAGCAGGAGGAGCTCA
GCCGCAAGTTTCTGCTCTTCACCGACACCATCCTGCGCAAAGTGCATGCC

789 CTCTGCGAGGAGCACTTCTCCCCCGCCTCGCTGGACCTCAAGTTTGTCAC
790 CCCCAAGGTCCTCCGGCTGCTTGAGATCCTACACGAGTACAAACCCTTTG
791 AGCGGCAGCAGTTTGAAAGTGTGGAGTGGTACAACAACCGCAACCAAGAT
792 AACTATGTGTCCTGGAGCGACTCTGAAGATGAAGACGAAGATGAGGAGGT
793 GGAGGCCAAAGAGAGGCCCGAAGCCAACTTTCCCTCACCGTTCACCAACA
794 TCCTGTGTGGGATCATCTTTGTAGAGAGGCGTTACACAGCTGTCGTCCTC
795 AATCGGTAGGGCTCTGTGATCTCCATGGTGTGAGAGCTGTACTTCACACT
796 GTAAACCAAAAACAATCCAGATTACTGACTTTCTATTGGTTTTTTGTTAT
797 TTATTATACAGTCATACAGGGTTGTTTTGTGGTCTCTAGTGTGTACTAAA
798 TGGCTCTTAAAAAACTAGAACTACCTTTTTAACTAGGAATGACCATGTTG
799 AAAACATGACATGCTACTAATATATTTGCAGAATTGCAGAAATTACATTT
800 TGACTTCTGGAAATTAGGGCTGCACAAGTAATCATAATATTATTGAAATT
801 GCAATATGGCCAAGGGCAATATAAGCACAGAAGTTGCAATTTGTTGATAA
802 ATGTTACAAACAGTAACATAATAATATTAAATATTATAATACATATGTAT
803 TATAATGAATTACTGTAGTGCTGCAGAGATGCTTTAAGCAGCAAATGTTG
804 TTCTCCAGATGTAGGAAAACATGTTTATTTGGTACAGACTCCAAGTAGTG
805 AAAATGAAAAATGTGATGCAAAAATGATCATTCCCACTAATCGTGAATCA
806 TATTGTAATATCTGTCAGAATGTAATTTTTTTTACTATATCGTGCAGCCC
807 TAGTGGAAATTCCTTTCTTGAGATCAAAAATTGTATTTCAGCCAATACCA
808 GATATTGTACATAGTATAATTGTTACTAGCAGGAATATCGTTTTCACCTG
809 TACACAATTGTATTTCAGATATCTGAAACGACATTCTTATAATTTGCTTA
810 AAATTCCAAGCTGAAATATCAATAATGGAATTCAGACTAGTCAGAATTGG
811 ATTTTAGATATCTATTATTCCATTTATGATCTCTGTAATGTAAGAACAAT
812 TGTAGATATTTCCACTATTAAGAATTGATTTATTGATATCATTTTTTTGA
813 CAGTTGTAGACATCAATAGAATTGAACTAGTCAAATGTACATTATGGATA
814 TGTCTTGGATGATTTGGAATTATGTTAAAAATTAATTGTAGATGTCTAAT
815 TGAGTTTGAATAGAAGTCAATGGTAAAAGCGGACTTGTTGTAATTCATTT
816 ACAGATATCTACAATGAACATTGCCATTGGTAAAAGTTTAATTGTTGATG
817 TCAAAGATTAAAGCTGCACTTTGTTTTGTATAAAAATAACTTTTTGTCAT
818 AGTTGCTGTGACATTAGTAGTACATGAGACAGATAACCTGTGAAAAAATC
819 TGGTTCCTCTGGCTCCTCCTAGTGCTCCTAGACTCCAGGCCTGGTGTTAC
820 CTAATGCACCTTTAAACTTTTCAATTTTAGATCCATAATTACATTATGCA
821 TATTCTGGCTTGGAATTCTAACTAGTCAAAAAGCAGTTGTTGACATCTAC
822 AATGGCTACTACCTCTACTAATTCAATGTAAAAAGGGCTTGACCTAGAGC
823 CACTGCAGCTGAGAGAAAGTGTAACATTGATGTGTCTTCCTCAGTCTGAT
824 CAAAGAGGCGGGGAAGCAGGACCCGGAGCTGGCTTACATCAGCAGCAACT
825 TCATCACTGGCCACAGCATCGGCAAGAACCAGCCGCGGAACAAGCAGATG
826 GAGGTGGAGTTCAGGAAACAAGAGGAGGTACACAGACGGCAATCATGTGG
827 ATGTAGACAGTAAACTAGGACAGTAAGCTGGAGACATTACGTATCTTGTT
828 CATTTGGCACATCCAGCTACCACAGGGCCTTTTTTTTTTTTCTTTTTCAG
829 AACTGCCTTTGACTGCTTTGACATTATCAAAGCAGTATTAAGACTCGATT
830 CTGTTCTGCCCTCCCTTTTTCTCTCACAGGTCCTCCGCAAATTCCGGGCT
831 CATGAAACTAACCTGCTGATCGCCACCAGCATCGTGGAGGAAGGCGTGGA
832 TATTCCAAAGTGTAACCTGGTGGTGCGCTTCGACCTGCCCACAGAGTACA
833 GGTCCTATGTCCAGTCCAAAGGCAGAGCTCGGGCACCCGTCTCCAACTAC
834 ATCATGCTGGCTGACAGTGAGAGGACCAAAGCCTTCGAGGAAGACCTCAC
835 TACCTACAAGGCCATAGAGAAGGTAACCTTTTAAATGAGGTAATAGGATA
836 AGAAAACTCCTACCATTATTCAAATAATCAACTGTCTCATCAAGGAGGTA
837 TGTTAGAAGAAATGATTTGAAGTGAAGCAGAATGAGTTGGTGATGAGCAT
838 CTCACTTTACTTTTGAGCAGATCTTAAGGAACAAGTGCTCCAAGTCAGTG
839 GAGGTAAGTGAGTTTGAAGTAGAGCAGGTGCTGGACGATGACAACATCCT
840 CCCACCCTACGTGCTTCGGTCTGAGGATGGAGGTCCCCGGGTCACCATCA
841 ACACAGCCATTGGACACATCAACAGGTAGGGAGATGTTTTTGCAAAGTCG
842 TAAGTGTCCCTATAAAGTCAAAAATCAGTTTAAAGTGTTTGTATCATTTG
843 TCTGCTGCTATACATCACCTATTCTAGTAAGGTCTGAATTTAAAGGAAAC
844 ACATTTATATAATAATAGCACCCGCTAGCCCTTTCATAGGAAGTTTAAAT
845 CCTCAGTTCATAAAGGAAATGACACAAACAAACAATAGCATTCCTTAGTG
846 TCTCTGGTGCTCTCATGCTCAGGTATTGTGCCCGGCTTCCCAGTGACCCA
847 TTCACCCACTTAGCACCAAAGTGTAAAACCGTGGAGAAGCATGATGGACG
848 CTTCCAGTCCACACTCTACTTACCCATCAACTCCCCTCTGCGAGTTCCTG
849 TTAAGGTAAGGAATTTTGACGCAGTGTACTTTTTCTGGGATTTAGAAAGA

850 ATGAATCATATTACAACTTCTTCAGGTTCATTTATTTTATTGATGTGTCA
851 CTGTGTATACAGGGTCCTACAATGAACTGTACCAGACTGGCAGAGAAAGC
852 AGTTGCACTAGTATGCTGTGAGAAACTCCACAAAATAGGTAAAACCAGAA
853 ATTAGATTTTGCAGACGCCTGTGTAGAACTTTTGCCTGCATCTGTCTTAG
854 CTTGTTTATCTGGCTTTCTTCCCACCCAGGTGAGCTGGATGATCACCTGA
855 TGCCGGTGGGGAAGGAGACAGTGAAGTACGAGGAGGAGCTTGACCTTCAT
856 GATGAGGAGGAGACCAGCGTACCAGGCCGGCCCGGCTCTACCAAGAGGAG
857 GCAGTGCTACCCTAAAGCCGTAAGAAAGTCTCTGCTGGAGTTCACTGACT
858 CTAGAATCTCAAAGCAAAAACAGTTTTCATTTGTTTCATTGAATGAATAC
859 TAAAAATACTTTGGACTGTCTGACTGACCTTTTCGTTTTATTGCTGATAT
860 ATTCTTATTAAAACAACTTCAGAGATAGAATTACTGTAGTAAAAACATTA
861 TCATTTTAAGTAATGCCAGGGCTATACTTAACTTTATTTGCACATAGCAT
862 TGGTGCTACAGAGGTTGAAAGTTTTTAGTACAAAACTTCCCCTACCATG
863 TCCAAAAACAACCAGTGCAACATTTTGCCCATAATTAAAGTCATTTGCAG
864 GGGCCACTTGTAATACATCCATGTATTACACTTGGATGTATTATACAGCA
865 GCAAGTGTATAGTACACTTAATACATTTATATATACACTCAATATGAAAG
866 CTAGATTTCAAAAGATTTACCAGAATGCAACAGACTGTGAGTCGGCAGAT
867 TGCCTCCAACTGAATACTACTTGCCTCACCCTCCTGTTCTAAGGGTGTAG
868 GAGAAGTAGTCTTCAGGTAACGTAAAAACATAAAAACTCAAGCCAATGTT
869 TGGTTTGTCCGTTCTCGGCTACTGTAAAAACATGGCAGCTCAACATGGTG
870 AACTCGGATTCCATTTCTGCCAATAGATCCTAAACCCTACACATTGGAGC
871 TTTTTAATGTCTGTTTATTTATGTGTCAGAAACAATGGGCATTTGAAATG
872 CTTGTGCATAATTGTGTACATCGTTTCTCTCCTTTTTGTCCCCTCCAGAT
873 ACCAGAGTGTCTGCGGGACAGTTAC**GCAGTACCGGAGCAGACTTAC**TACT
874 TATATGTGATTGGGATGGTCCTCACCACCCCTCTCCCGGATGAACTCAAC
875 TTCCGAAGGAGGAAGCTCTACCCACCGGAGGACACCACCAGGTGCTTCGG
876 CATCCTGACTGCCAAACCCATACCTCGAGTGCGTTGCCTGGTTAAATCCT
877 AAATTCTCTCACACACATCTATACTTCTATAAATGTTGAGCACCGTCCTC
878 TCGTCATTGGTTTTGGATGATA**AATGATGCTTTCTTTCTGTCC**AGATCCC
879 CAATTTTCCGGTGTACACTCGCTCGGGTGAGGTGACCATCTCTATCGAGC
880 TGCAGAAGTCCGGCTTCGCACTCACCGCCGCCCAGCTCGACCTCATCACC
881 CGCCTGCACCAGTACATCTTCTCCCACATCCTCCGCCTGGAAAAACCTGC
882 CCTGGAGTTCAAGCCCACACTGGCTGACTCGGCGTACTGCGTTCTACCTC
883 TGAATGTTGGTGAGTAGAGTCATTGGCAGGCAAGCTTGGTGGATTACTTT
884 TCCAGATGTGTGGAGATGCTGATTGGTTTCCTTCCATAATCCTCTGCTTC
885 TTTAGTTGGGGACTTGAACACACTAGATATGGACTTTAAATTCATGGAGG
886 ACATTGAGAAGTCTGAGGCCCGCACTGGCATTCCTACCACTCAGTACACC
887 AAGCAGAACCCCTTCACCTTCAAGCTTGAGGACTACCAGGATGCTGTCAT
888 CATTCCAAGGTACCATATGAACTCCTTTATGAGATAATGGCAGAGCGGTT
889 TTACTGTGTGTAAATGCTTCCAAGGAACAAACTGACACGGACTGCTCCCC
890 TCCCAGGTATCGCAACTTCGACCAGCCTCATCGCTTCTATGTCGCTGATG
891 TGTACACAGACCTGACGCCGCTTAGCAAGTTTCCTTCACCAGAGTATGAG
892 ACATTTGCTGAGTACTACAAAACCAAGTACAACCTTGACCTGTCCAACCT
893 GAACCAGCCGCTTCTGGACGTAGACCACACCTCCTCCAGGTCAGCAGCAT
894 GGGGATCATCAGGGGGTCTTAAAAAGTCTAAGAATATTATCGCAACTGCA
895 ATATGCAAATCACAGAAGCTGCAATCTTTTGATGAAGGTAAAATTGGTGA
896 CAAAGCAGCATCATAATGAAATACTCTAGTGCTGCAGAGATGTCCTGGCC
897 TATACATTTTATTCTCCAGATTTAAAAAAAGATGTTTGCTTATTACAGAC
898 TGCCAAAACGTTCACATCATAATTTTTTTATGTTTTTTGTTTTCTTTTTG
899 TAAAAATAAAATCTGTCAAAATAAGTGCAATATGATATTTTTACCTTATC
900 TAGCCATAATTGGCATATAATAGGTCTTAAGAAGGCCTTAAAAAGTACAT
901 TTTGACCTGTTGAAACCTGCAGAAACCCTGGATCATGACAGTTAAATATA
902 ACTGTAAACCACTATCCACTTCTACACTTCACAGTGTGTTTTCAGGTTTG
903 GGGAGTACTACTACATTTGTTACAAAAGTTGCATAAACCCTTTTGTGTCT
904 CCAGAAGGAGCAGTGCGTCTGATAAATTGCCTCAAGTAATCTCACTTGAG
905 TGATGTCACTTGAGTCAATGTCAGTTTGCGCTGAAGACGGCAAGATAGAA
906 AATGAAAAAAAAAAACTGCTGGTGTGGAGCCCCCTCATCCTTTCTGCTTG
907 AGGCCCTGGCCTACATTAGCCACTACTAGCACGATGCACCCATATCTCAG
908 ACAGAACTGACAGCAGTTGAGTTGCATTGTGGATAATTAAGGCACTAGGT
909 ATTGTCATGGAAGGAGAATGTGTGGAATATAATTTAAAAGAATAAAGAGT
910 GCAGTTTTTAGAATATTCTGGCTTAAATCATGGTCTTATAATGTAATTAG

911 CAACAGCCAAATGTGAAAGCACAGTGTCATAAAGTCACAGTGTTAGCATG
912 CCCCTGCATTCTTTTTTTCTGATGGGTATACAGTTGACACTCTTGTTTTC
913 TTGCGCTCTCAGACTGAACCTGCTAACCCCTCGGCACCTGAACCAGAAGG
914 GGAAAGCCCTGCCCCTCAGCAGCGCTGAGAAGAGGAAGGCCAAGTGGGAG
915 AGTCTGCAGAACAAACAGGTAACGGCTGCTTCTGTTGTCTTACAGTGAGA
916 ACAATGTGTGAAACCACAGGGTCCCCAGGCAAAACGTTTGCTGCAGTGCA
917 ATGCAGTGTCATTCAATAATAAACGGCATATACCTTGTGCTTGTTGAGAA
918 GGTGTATAAATGTTTTTTGCTGTGATAACTGACCAGATTTGCTGTGCAGT
919 GTATTTGGTGTCTGATAATATGGTTCCCTGTCTAAAATGCGTGATTCTAT
920 AGATACATGCAGGTCCCTCAAATAGCATTGTTCATTTGTCGGTCCATTGT
921 ACTGCTCTTTATTTATTATAAAGTTGTAATATAGTTTGTCATAAATCTTT
922 ATTTCAAAATTTCCTTTTTCATTTTTGCATTTTCATCATGGAAGTCAGTT
923 TAATCCTTGTGAATATTTACTATGGAGTGATCATTTACTGTGTATGTGCT
924 CTGATCCAGTATAGTCACTGGTTGAAAATACCTGGTTTTCCTTACACAAA
925 GCACTGCTGTTGGTATACATTTAGACATGGTGTGTGAGCTTGAGTGTGAG
926 CATCACTACAGAAGTGAGTTTCAGCTTCATAACCTTACGTTGGTTCTGTA
927 CTGCAACAGTCCTGGTCTAATGGTGTCTCGTGTTTATAAGATCCTGGTTC
928 CAGAGCTGTGCGCCATCCACCCCATCCCCGCCTCCCTGTGGAGGAAAGCA
929 GTGTGTCTACCCAGCATCCTCTACCGCCTCCACTGCCTCCTGACCGCCGA
930 GGAGCTCCGAGCCCAGACAGCCAGTGAAGCTGGAGTGGGAGCCCAAACCC
931 TGCCCCCTGACTTCAGGTCCCCTTTACATACTCTACACAACATATGAAAT
932 TATCCACTTTCAGTTATTCCATTTGCTGAATCAGAACCTTCTCTTCAGAT
933 TTGGATCATTTGTGTTTAACCGCACAGTTTCCCAGCATTCTAATGTATAG
934 ATGTGTATGTGTGTGGCAGGTATCCAAACCTGGACTTTGGCTGGAAGAGA
935 TCTATTGACAGCAAAACGTTCATCTCCTGCCCGGAGTCCTGTGGCGAGGA
936 CGGAGAGGGTCACTGTAAGCACCAAGAGACTGTAACCCCTGACCCCAACT
937 CCCTAACACATCCCAGTAGTCACCACTCTCCCCGGCCCCGTGCAGCCCTC
938 GAGCCCGGAGAAGCCACCTGCACGAAGAACCTAATCAATGGCACTGCCCT
939 AGTCGCCAACTGTGACGACGAAGGCCACCAAGATGAGCACCTTCACCAAC
940 ATGACAATTGCCAACGCTCCCAGCCCAGCTCCCCTGGGCTGCAGAGCCCT
941 GAATCAATACAAACCACTACCTCAGTTCCTGTGCAGCCCTCTCACAGCTT
942 GAAGAAGCCCAGCTCCAGTCCTCCGCAGCCTAGTGATGAATGTACACCAG
943 GGAGGACCTCAGACCACAAAAATAAAGCTACCTCAGTCTGCAGCCGGGCA
944 GCCACGGGTCCCAATGCTCCCACAGCACCTTCTCCCGACCTCGCCATTGT
945 CCCCCAGCTTGGAGCCCAGGCGGGAGACTCCCCCAAAAGCCTGGGGCCCA
946 ACCCGGGCCTCATCCTGCAGGCCTTGACCCTATCCAACGCCAGCGATGGC
947 TTCAACCTGGAGCGGCTGGAGATGCTTGGAGACTCTTTCCTCAAACACGC
948 CATCACCACATACCTGTTCTGCACCTACCCCGACGCTCATGAGGGTCGAC
949 TCAGCTACATGCGCAGCAAGAAGGTAAGACAGGTCACGACTTGTGGCTTC
950 ATAAGGGGAATGACAGTTGTGGTCAGAATCAACAATTTACTTATCAGACT
951 TTATTGCTTCTCTGAATGAATCTTATAAATACTAATGTACTGTTAGAAAT
952 ATTTTCTGTGCTGTGGTATTGAATGTTGTTGCACAGCTTCACAAACACTT
953 AGATGGGCAGTTTAGTCAGGGATATGCTGTAACCAGATATGCACACACTA
954 CTACTCATGCACTTTGAGTCAAGATATAATTTGTTTGTATCATCAATTAC
955 TCCGTTGATCATTTTCTTGATTAAACCTGTAGTAGGTAACTTTCCTGAAA
956 AATAGTTGTTTGTCATATTTGCTGAACCTGTCACTATTCTAACAGTAGTA
957 CATGGGACAGATGAATTGTGGAGGAGGAGGAGGAGGAGCCAGAGGAACCA
958 GATTTTGTTTCACAGATTATCTGTGTCATGTACTACTGTCAGAATATAGT
959 GAAGGTTTCTGCAGATATGACAAATATGAATGTTTTTTTTTATTTTTTAT
960 AAAAGTTACCTGTAGCTTTAAAGGTCATGAAAACACGGAAGTCGGAAGAA
961 TGTGTTCCCAATCCCAACTCTGGAAACATCTATTAGTATCCAGACAACAA
962 TTGTACCTGACTGTTGTATTAAAGCTGCCTTAGGCTACAGTATGTTACCG
963 ATGATGCAGGCATCGACTCATCCTATTTGGTCTGCCCATGCCCAGGTGAG
964 CAACTGTAACCTGTACCGTCTGGGGAAGAAGAAGGGGCTCCCCAGCAGGA
965 TGGTGGTGTCCATCTTCGACCCCCCTGTTAACTGGCTGCCCCCTGGCTAT
966 GTGGTCAACCAGGACAAGAGCAGCACAGACAAACTGGATTCAGAGGAGGT
967 CAGTGAACGTCCTGCAGCTCTGACTTTACGCACTCTGTTGCCCCTTGTTT
968 CTTCTGTGTGACTGTGTGTGCTGAATTGTACAACTGCAGAGTGACAACAT
969 GTTTCTTAGTCTAACCTCTTCAAGGTGCTGGATTTGGTTTTGGGATAAAG
970 ACAGGAATTAGCAATAGGTATTATTTGTAAGGGTTAGACCTAGGATTTAG
971 AGAATGACTTTAGAGAATACATTATTTTATTCATTTGTCATAGATGTTGT

972 TAGTGTTAAGATATAGTCAGGCCACTAGAGGGCAGCCCTTTACCTTGTCT
973 GGCAACACACCATGCTTGTGTTTTGGTTTAAAGTCCCATAGCCTTTAACT
974 CTAGGCTCTACTCCTACATATCATATAATAATGTTGACAGTGTCTCACAA
975 CAATTTCTTATTATGTTTCATTTATTATAACCCAGTCTTGGATCAAACCG
976 CTCACTGAACAACACCTCAGTCATTTAGTAATATACGTTTATCAACTGAC
977 TGATTTCGTTTTTTGACATATTTGCTGAAACTTTCACTATATCCTGAGCA
978 GTTAGCTTCAAGCCACCAACCAAGTGTCTTTCTGTCTTTGCCCACTCTGA
979 TTGGTTGTTCTCTCCTGCTCTTTGTGTACATCATCTAGGCAAAAGAGGAG
980 CTTCTGGCCAACGGGCAGTCTGGAAATGACTATGATGAGGACGATGAGGA
981 GGGCGAGGAGGTGGACGAGGATGGTGAGCTGATGCTGAAGGACGAGCCAA
982 AGGATGAAGTCAACATGGAGGACGATCTGGAGTATTACAAGGAGCACATC
983 AAATTCATTGACAACATGTTGCTGGGATCTGGTGCTTTTGGTAAGAAGAT
984 CTCTCTGAGCGGCTTCCCTCCCTCTCTCACCCCGGCCTCCGGATCCTCCC
985 CTGCCATGGAGTCTCCGTACGAGTGGAAAGCGCCCAAGAAACCCCTCCAT
986 CCCACCCCGGCCCACTACCCCTCAGAGCCGGTGCACAGCGGGCCGTCTGC
987 AGAGGAGTTTGACTACAGCTCGTGGGATGCCATGTGCTACCTGGATCCCA
988 GCAAGGCCGGAGAAGAGGACGACTTTGTGGTGGGTTTCTGGAATCCATCG
989 GAGGAGAACTGTGGTGCCGAGCTCGGCAAGCAGTCCATCTCCTACGACCT
990 GCACACAGAGCAGTGCATCGCCGACAAGAGCATCGCTGACTGTGTGGAGG
991 CTCTGCTGGGCTGCTACCTGACCAGCTGTGGAGAGCGAGCCGCCCAGATG
992 TTCCTCTGCTCGCTGGGCCTCAAGGTGAGCTAGGACCTCCCACACTGTAG
993 CAGGATTTAGAGATTATAGCTTGGGTCAGAGTACAGCATAATATATTGAC
994 AAGTCAAGCCACTTGAGGTTATTCACGATTATCACCGTAATCACGATGGA
995 TTTTATTGCGATAATATAAGTATCCCTATATGTGACCCATCCTGGGCTGG
996 AAACGCCCACACTGTTTGTTATAGGGTCACACAGTCAGTGGAGCACGGTG
997 CATCCCTTTCAAGTGTATACCATGTGTTGCCAGAGGCTTTCCCCCCTTTA
998 TATGTAATTGTTTTGAATTTAGACATTTTAGCTTGACATTTTTTAACCAT
999 GTTTGAGATAGCTACTATCTAGCAGTAGGCTACCACTTGCTGTAATGTTA
1000 ACTGTCTATTTCAGAAGCAGAATCAGCAATACTTATTGGGGAATTTGTCT
1001 GTGACAATTGCTCATGGTGCTCACACAATGTTAAAGAATAGAAATACTTA
1002 TTTTATAACACAATTTACAATGTCTAACACAATATATATAGAATAGAAAT
1003 CAATAGGTGTATACATATCAGTAAAAATGGAAATAGAAATGTGATAACGG
1004 CCTTAAATAGACAGCTCTGAAGCCAAAACGAGGTACCAACATCTGCATTG
1005 TGATGTGATCCAGCACCATATTGGCACCAGCTTTCAGTACCTAGCAGGTA
1006 GCCTGCCGTTAGCTAGTACATAAACACTGTTAAAATGCAGATTTACAGTC
1007 TAAAACGTGACTGCATCTGACTCGAAAGGATTCCAACTGTGGGTGGGAGT
1008 TTCAAAGTGTAAAGAACAGACATACTTGTCTGTCCATAAGGAAATGCTAT
1009 TACACTGTCCTAAAGCTAGACTTTTTTATTTTATTTTTAGCCTAGCAGCT
1010 GGAGGCTGGACCAATCAGGAGCGTGAAAAATGAAACATTTAGTTGGTAAC
1011 TGCTTGTAGTTTGAGTGTGAAATTTGAAACCCAAGTCCAGTGGCAGGCTA
1012 GTCAGAGGTACATCCTTCCAACCAGTTGTTGGAAGTTCACATCTGTCCCA
1013 AGATAAATTGAAAATAACAAAACAAATGCAACAAATTGCAGTCAGTTAGG
1014 AGAAAGCCACAAGACAAGCTTTGGGGAGGCATCAAAAGGGGACGCTGACT
1015 TTCCCTGCTTGACATCTTTGCGCAGGAAGTTTCACAGCTGAGACCTGGAC
1016 AGCAAGGCCCTGTCACCCTTAGTGACCAGGCAAGATTTTGGAACTACTGC
1017 CAGAAGATCTCAAGTAGCCATCAGACTCATAGGGTGTTAAAGATGTGTGC
1018 AGGAGCCAGACCATGCTAGGTGCTCGTCCAAGACAAACTATCACTGGGGG
1019 TTTACAGAGATATTAACAGTTGTTTTTGAAAACAGGTGTTACCATTGGAG
1020 AGAGGGACCTTGACAGGAGCAGTGGTGCAGAGCCGCCAGATCGCTGCCAT
1021 CGACCTGTGCTACGGCTGGCTGAAGATCCCACCCCGCTGCATGCTGGACC
1022 ATCCCGATGCAGAGCGGACTCTGAACCACCTCATCTCTGGCTTTGAGAAC
1023 TTCGAAAAAAAGATCAACTACACGTTTCAGAACAAGGCTTACCTCCTGCA
1024 AGCCTTCACGCACGCTTCTTACCATTACAACACCATTACAGGTCAGACTG
1025 CACCACATAGAAGCAGGACAGTTTTACAAAACAAGTAAATAATACTATGT
1026 GTTAGTGCTGTCAGTTTAACGTGTTATTAACGGCGTTGACACAAACCCAT
1027 TTTAACGGCCTCAATTTTGTTATCGCGAGATTTACGCCTAGCACACTTTG
1028 TAGTTTTTTTCACATGCTGGTGCAACAACTAGTAACGTTAGAAAAACTAC
1029 AACACCACACCGGATCTAGCTAGACCGGAAACAAAACAACAGGTACGCCG
1030 CACACACTTGTTTAAGTGTAGTAGATGTTACGTTTTGAGTGGATGGTGAG
1031 CGCGAGACGCCGAAATGGATGGATTCCTTGTTGTCTTAAAATGTCAACAG
1032 GCTTGTCTGTTTGAGTAACTGGTTCAAAGCAAAGAAGTAGTAGGCTTACC

1033 TTTTATTGGTAACCGTTACTGTTCATTCTTTCTGAAATAAGAGGCTGGGT
1034 TGAGCCTATGGTGAATAAAGAGCAGTAGCCTGACTGCTATGTTCAAAGCA
1035 AACTTGAGAAAAGTATTAAGCCATGGTTTAACTGCACTATAGGCTGAGTC
1036 CTAGTTTACCTTACATGTGCACTTTGTAATTTTATTTTGTATCGCCCTGT
1037 TTTGATCCCTTAGAAAGGGCTGTTGAAGGGGCTGTTTGTGTGACAAAGTA
1038 TTTTTTTCACCCTTTTATTGATGGACAGTGTTACATTGTGTGAGAGATTA
1039 TTTGCTCCAAGTGAGAATGTTAGTGTGAAATTGAACACTACAGTATATGC
1040 CAATGTTGTTTTCAATAAAAACAACATTTGCACAAAGCAAGCCGATCCAC
1041 TTTTCCAATTTACAGACCAAACAATTATTATTAATAATAATAATAATAAT
1042 ACATTTTCAAAAAAACTCAAAGACACTTCGCATAAGTTAAAAAGGACAAG
1043 AAAAGGGTAATAAAAACAACACTAAGAAATTATAGCTATATCTACAACAC
1044 TCAACAATGAATTAATCGTTAAAAGCACTTCTGAAAAGGTGGGTTTAATC
1045 TGCAGATTATTTGAGAATAAAAAATAAATACCTGTCTGTGTTCACCAGCT
1046 AGTCTCTAACTGTGTCTGCCGAGCAGGTAGACTTAATTATCAGGTAGAAA
1047 ATACACTAAAAAGCCTACAACACAATGAGAGCTACAACAAATCAAAGTTG
1048 TATTCCACCTACCCACATATCTGTCTTTGGTTTTATTGTGGCTTCTCTTC
1049 AGATTGTTACCAGCGGCTGGAGTTCCTTGGTGATGCAATTCTAGACTACC
1050 TCATAACAAAGCACCTTTATGAAGACCCGCGGCAGCACTCTCCTGGCGTG
1051 CTGACCGACCTGCGCTCAGCACTCGTCAACAACACCATCTTTGCCTCTCT
1052 GGCTGTCAAGTACGACTACCACAAGTACTTCAAGGCCATCTCGCCCGAGC
1053 TTTTCCACGTCATAGACGACTTTGTGCAGTTTCAGCTGGAAAAGAACGAG
1054 ATGCAAGGCATGGACTCCGAGGTGAGTCCCTTTTCCTACTAGAAACGCAG
1055 CTAGCAGCAGTACTGAACAACAAAGTCCTGTTGGAAGAAACCTAATCTTT
1056 ACTGTTGTAGTTTTTTACCCCCCCTAAAATAATAGTTGGCATTCAAAAAG
1057 ATCTGCCCGAATAACAAGGCTGTCAGCTTCTATGAATCTGTTTCATATTG
1058 CAATGATTTCAGTACTGAAGAGAAAATGCAGTTGGATTTAATTTGCCTGG
1059 AAATGCCTATCTTGGCACCAGATCTCCTCTCATTCTGCTTGAGAGGTACT
1060 TCTATGCCTTGGGACAGCAGGCACATAGACAAGCGTACTGAACAGCTGAA
1061 AGAAATAAAGAAATGCACTGCAGTAATATGGTACCACTCCGCTGCTCACT
1062 ATTCATCAAATAAGACTAACAATTACATGATGAAGCTGCTAATGTGGCAT
1063 GCTTTATACTGTTAGATGTAGTGTCAGTACATTAACAGACAGAGTCTGTC
1064 AGTGATGTATGCAGTTAAGAAAGTCTTTAGGCTCATAAATAAAGGCTAAA
1065 GGGCAACTTAACATGTTGTTTTGCCTTGAACACACCCCCCTTAGACAACCT
1066 AGATATAAATCTGGTGCTCATTCAGAATTCATAGAACCGTAGTTGCATTG
1067 ATAACCTTGATTAAACGGCTACAGCAGCCAGTAGCCACTTGCATTTGTGT
1068 CATAGGAAGCAGTGACAGGGATTCACCTAGACTCAAGGTGACACCTTCAC
1069 GGATGCCCCAGGTGGTGGTGGTGGTTCAGAGCCGAATCACTAGTTTAGCA
1070 CTTCAGCAGCAGAGGCAATGAAGAGTGACACTGTGGGATGTGTATGGGTG
1071 GGAAGTATAAATGTGTTAGACTGACATCGCCAGCGGATTACTGTGCAACC
1072 TCTCCAAGGCTGTTATCGCTAGGCGTACACCAAGACCTCTGGATGCAATT
1073 GGATGGATCCACAACCAATCAGGGCAACGAAACGTGTGACATAGCGCTAA
1074 GAAAATGCCTCAACGCTGCTCCTCTGTATCAAACCCACCATGTCATTGTA
1075 TCAAACCCACCCAGTATTACATAAACTGTTGTTAGGGGGCCAGACAAATG
1076 CCAGAGCAAATCAAATACAAGCTTGCACATTTGCCTGGTTATAAATCTGC
1077 GATGTGGTTAAAATCCATAATCGCAAGTGGTGTAATTTAATATTAACATA
1078 TATATTTATACATTTTCTAGTAAATCAACTGAGACACTGTTTGTGGATAA
1079 AAAAGCAAATGTCCCGGCATCAGCTAACAGCTTTGTGTGTTTTCGCTATC
1080 AGCTGCGACGCTCTGAAGAAGACGAGGAGAAGGAGGAGGACATCGAGGTC
1081 CCTAAGGCCATGGGGGATATCTTTGAGTCGCTAGCGGGAGCAATTTACAT
1082 GGACAGCAGGATGTCACTGGAGACGGTGTGGCAAGTGTATTATCCTATGA
1083 TGAGGCCACTTATAGGTGAGGAAGACGGAAAGCACATTTATTTATTTATT
1084 TCTGTCTGCAGGGAACTTGAGTGATCAGAAACATTCAGGATATTTACCAT
1085 TTGTGTTGACATGAAATATTGAGGGTCTGAGGATTTTTAATTTTTTTTAC
1086 CTCACAACCCCCTGTCCCTTAACCACTTTCAGAGAAATTCTCTGCCAATG
1087 TGCCACGATCTCCTGTGAGGGAGCTGCTCGAGATGGAACCAGAAACTGCC
1088 AAGTTCAGGTAGGAGATCAGCACCTGTCTATATAAGTTCTTTGAAGTTAA
1089 ATTTCATGGTTTTAAATGAAGGTGCAGTTTAAGATAACTTAAGTTTAAGT
1090 AGAACAAATGTATTTCTTTGCACACAAAAAATTACTTTTTTTCCAATAAG
1091 AATTCATTTGGTTCATAAAGATGGGTGTGAACTGTATGATTTTAGAAATG
1092 TTGTTTAAATCAAACCCCCACTGTACGAGTAAATCGTCTTCTAAGTAAAG
1093 CTAAAACTCATGATTTAGGTGCTCACACTGTACAGTCTGATCATGCTCAC

| | |
|---|---|
| 1094 | ACTGTATGTATGTAAAATAGAAAATAATACGACAAATCGACCCCTGTGGA |
| 1095 | CTCTTCTGCCTATTTATAGTTGTCAATAGAGTTTTGTTTGAAAACTCCAA |
| 1096 | GGCAGGTATAAGTTTGTTTGCATCTGTGCCGTCAAAAATCGTAACTTAAC |
| 1097 | GTATCGCGATACAGGGGTTATGATTCAGTATATTGTGCTAAAACTGTAAG |
| 1098 | AAAGGCTACATTTTTAAAAATCACATTTTAGGAATAGTGTCTTGCTATAA |
| 1099 | AAAGAACACATGCATAAAATCTGAGTAAAGTTTACTTTATCACAAAACAA |
| 1100 | TATATTTCAATTCAGTTCAATTCAATTTTATTTGTATAGCGCCAATCTTA |
| 1101 | ACAGAAGTTATCTCAGGACACTTTTCAAATAGAGCAGGTCTAGACCGAAC |
| 1102 | TCTTTAAAACATTAATTACAGAGACCCAACAGTACCACCATGAGCAAGAA |
| 1103 | CTTGACGACAAGGGCAAGGAAAAACTGCCTGTTTAAAAGGCATAACATCA |
| 1104 | CAAAACGCGATATGATATTTTTCTTTATTTTCTTAAACCCCTAGTTGTGA |
| 1105 | TAGGAGGAGGAAGAAACACCGAAAAGGCTTGCATGTTGTTGCCACAGCTC |
| 1106 | AAGCAGCATACATATTTTTCGTCCACAGGACCCATCGTGTCGCCACGGTG |
| 1107 | CTTACAGATGGTGTGTTTGACAATTTGCGCAGGCACAGTGGGAAAAAAGG |
| 1108 | TACATACTTTGGTGAATTGGCTAGTAAGTCAGATTCAACTGCGCAAGAGC |
| 1109 | CTGAGCTGATGTCACAAATCAGCTGAACGTTCGGGAGCTTTTTTATTGGT |
| 1110 | CCTCGCTCCCCCTTTACAAAGCATGGCACACAACACCCAGCAAAGCCAAA |
| 1111 | ATGAGTTAGAAACTGGCTCAAATGTTACAGTGTATGCCAGCGTAACGTGG |
| 1112 | CTCTGTAATTGTACGGCTTGAATTAGGGATGGATCAAGACAGTTTGCATT |
| 1113 | ATATGGGAAACCGGTGAAATTAGGAACAAAGGTTTATGTCTAGCAGCCTG |
| 1114 | TTTCAAATAAAGTCCTTGCTCTCTCTGCAGTTGGGAATTTTGGGTACCTA |
| 1115 | CATTGTGAAACCCTGTAACCTTTCCTCTGTGTGCAGCCCTGCAGAGAGAA |
| 1116 | CGTACGACGGGAAGGTCCGGGTGACAGTGGAGGTCGTCGGTAAGGGCAAG |
| 1117 | TTCAAAGGAGTTGGCCGCAGCTACCGGATCGCCAAGTCGGCGGCGGCGCG |
| 1118 | ACGAGCTCTGCGCAGCCTCAAAGCCAATCAACCTCAGGTCCAAAACAACT |
| 1119 | GAGCTCAGCATTAGCACCTAAGCTATTGACGCTAACGGAGAAAAAGCAGC |
| 1120 | ATTGGATTCCCAACATAGCGCAATGCTAGCCCTGTATCTTCAGCCTGCGA |
| 1121 | TGGCACAACCAAGGCAGCCAGGGAACACTGTGTGGAGACGCAACAAAAGA |
| 1122 | AGCAGACTGATCTCACAACAGATTTCACATCGGATCTTGTACCTTTTTTT |
| 1123 | TGTTTTGTTTTGGTTTTTTTTGCGCAAACACAATCTTTACTTTCCCTCTC |
| 1124 | TGCTTTGTGTAATCACATGAGTGCTTTATTAACGTTTAGCAAAGTGTTTA |
| 1125 | AAAAAAGGTCAGGTCGCAATGTCTGTTCCTCACTGTTCTTGATTTTTCTT |
| 1126 | TTGTTTGGGTTAACCTCCATGTCAGTGAGATAAGGCTTCTGCTTATTGTG |
| 1127 | TAGTATTAGACTGTACATAGTTCAATCAAAGGTGTAAATCAAAGTATGTA |
| 1128 | GTAGGACGAAAAGAAAAAAAAAACGCTAAACTCCTCAGCCTGTGCACTAGT |
| 1129 | GCCAGTCAGTTTTAAAGCGGTTTTTAAAATGCTAACGGCAGACGTGGCTA |
| 1130 | ACAGATACCACTGGAGCAAAAACCTGGTGGCACTTCTGTCTGAAATCTGT |
| 1131 | CTTGAAATGGTATTCTTAATCATATTTGCACTTATTCCATTAGGGTCTGT |
| 1132 | TACTTTGAGAATTGTGTGGTCACATTGACACCAAAATCATATACGTAGCA |
| 1133 | AAAAATATAAATATATATAAAAAGAATAAAATACATGAATGATATTCTTG |
| 1134 | GCCTTAAATGCTAAGGCTCACCCTCATATCCTGCAAAACGGATAGGAATT |
| 1135 | TCAACATTCCCAGTTACTGTAATTTAAACTAGTTTTTAACCACACTTGAG |
| 1136 | GTTGCATGTTCTTGAAGCTTATGTATAAACCACGACAACTGGATGGCTTC |
| 1137 | TATAAATGTTCTAAATCTATGGATATATCTAGAGTGTGTGAGAGCGAGAT |
| 1138 | CAGGTGTTTACGGCATTATCTTGATGATTTGGTTTAACATGTTTTGATAC |
| 1139 | CAAGAATGGATGGAATCGAATTAGTCATTCTTGCGCACTAAATGTCTTTC |
| 1140 | CACAGAGCGAATCATCCAGTTTTGATATATGTACGAAAAGAAAGACATAT |
| 1141 | CACACATTCAATATGCTGCAGACTTTTCTACAAATTGGATACTCGTGTTA |
| 1142 | AGACACACAGAATCTTTTCAGATACAGTCTTTTGTGAAAAAGGCCAATTC |
| 1143 | ATTTCAACACACTAGTGTTCCTCACATCCCGTACCTACCAAAGCTATAAC |
| 1144 | AGATGTGACACTATAAGGTTGAAGTCAATTCCTAGGGATAAGGATACCAG |
| 1145 | CCTTTTTTTTTTTCTTTTTTGTGGTTTCTGTTCTGACGAATTTTGCTTCT |
| 1146 | CGCTAAATGTCTTTTTTCTCATCAGTAAGCCCTTGTTCACTTGAATACCT |
| 1147 | CAGTTAATTGCATGCATTTTCTTTTCTTTTTTGTTTGGTGCTATGTTTTT |
| 1148 | GTATTAAGCTTGAATTTCTCATCTTTTTTGCACTGTAACTATAATACCTC |
| 1149 | TTTATTTGACCTTTTAAAAAAAAAAAGAGAATTAATTTGATTTTTTTTTT |
| 1150 | TTTTTCTGTTTGATTTGGTTGTCCTCTTCGTCAGCCATCAAGCTGTAGAG |
| 1151 | AAGAGCTGCGTTGTCGTCTTTTCCCCCCTTCCGTACTTGAGGTTCAGACT |
| 1152 | TGATGTATTTTTTTTTTTTTTTTTTGGATTGGGGGAAGAAGAGGAGGAAG |
| 1153 | TGTAAGCAGGAGATGTATTCTCTGCACATAAGCCTATCTGCATCCCCCCT |
| 1154 | CCCCCTTTTGTTTATTAACTGTTAGTTTCATCAGTTGAGAATCTGTTTCT |

1155 GAATTCTGGGTAAAAACAAACAGAGGAAGGATTGATTGATTGATTAATTG
1156 ATCAGATGGGTAAATGTTTGTAGAGATAATCAGATTGCACTCTTAAACTT
1157 TTAATTTCTCTGACATTTACTTCAAAACATAGAAATATATGGGTTGGAAG
1158 AGACACATACCCACGTGGTTTCTGTGTTAGTATTTGAAATCAAAGTCATA
1159 GTCCTGTAATGCCACCCATAGGTGCTTCTACCTCAAGGACAGTGCGCTCA
1160 GTGTTCATTTCTGAACAAAACTGCAGTTAAAGTCAGTAACTTTCTGACTA
1161 TATTACATAATGTAGTCTTTACTGTGATACCTATACAGTCCATGTCACAT
1162 ATGTAGTAAACAACACAGGTATCTGCTTCACTGAGTCGACATTCCCTTAC
1163 AGTGATTTTTGTCTAAAATAGAAACAAACTAAAGTCAGTGCCTTCTCTGT
1164 GGGAGATCAGTGTCAGCATAGACTGGATTTAAATTCAACAAAAAATTTAA
1165 GTTCAATTAATGAATCAAAATTTGAGGTTGTATATGCGATTGTTTGTGAT
1166 TTTGTTTCTGCTTAAAAAAGTGATAAGTAAATCAGGTATTTTCAACTGAA
1167 GATTTTAACCAGGTCTTCTCTGCCACATACGATTTGTAAGTTTACCCTAC
1168 AATATATAAAAATAATAATGTAACCCATTAAACCTACCCAAATACTTTGG
1169 AACCAATTAAAGAACAAGTGACAAACAAAAGAAGACTGAACTGACCCAAC
1170 AGAAACTGAAAGTAGTTCCAGTAAATGTTTCCTGATACTTAATTAACCAT
1171 GGCCCTAGAACCACTTGGGGTCCAGGGACTCCCTGGTTCACTATGTAGCA
1172 ATGAGTTTTTCAGTTTGTTTGAAGCAGACTTGGTGCCACCTTATCAAGCC
1173 CCAAAACATAACCTAGCATTGCTGCAGGGTTACAATGTTAGCCTCTGGTT
1174 AACATGACAAAAACCTTTCATGTAGCTTGTTTGTGTTGAAAAGAAACAAA
1175 TTTTAGAATTTGGATAAAAGATAAAAACGACCTGTAGTCACAGCTTAGGT
1176 TAGGTAGCTACTGTTAGCTATTGTTATTGTAGCTGTCTTTTTGCTAACAG
1177 TATTTGTAAGCTAGTGAGTTAGCTAGTGTTTTGCTACAGACCAATAAATG
1178 GTTAACTAGCCAGCTAATCTACATCAGTTGTTAGCTAGCTAGATACATCT
1179 GAAAGAGACAGTTTAGCAAAATGCTATCCAACCAGTTTAGCATTTCTTGC
1180 TAACATTATTTAGCTTAAGATGTGACTATATGTATGCCACCTTTTTTTTT
1181 TTTTATCTTTTAACCATCTTTAAAATGTGGTTGTCTTTTCAACACAGACA
1182 AGCCTAGCATAAGCATATCTAAGTATAAGAAAGGAGTAAGGAGTTCTGAA
1183 GAGTTATTGGAGTAATCAGGGCTGGGACATTTCAGACAATACAGATTGTC
1184 CCCAGACCCAGGGTCAGCTTATCGGGGTTCAGGTCAACCCAAAAACCTCT
1185 ACTTGACAGTGAAATGAGAACAAAACTTCCAATTACAGCTCAAGTCCAAG
1186 ATATGCATTTTTAATATGTTACGTCGGGTTATGTGAAAAGAACACTTACA
1187 TTCAAATCCAGTCTTTGCTAGGACTGCTCTTTATTTTTGTTGTCTACCAA
1188 CAGCTAAACTCTCAGACCTCTGGTCGGGTGCCATAGATAACCATGTGAAT
1189 ACTGAAGTGTAGATTTGTGAAATCCCTTCGTGTTAAAAATCAATGTGAAC
1190 TCAGTGAACACCGTGAAACTTAAGTACGTCTCTGTAAAATCTGTCGCTGT
1191 TCAAAAGAGTGCAATCTGTCATTATTTCTCAGGCAGCTGTCTGACCCTCT
1192 AGTCCACCTTTTTAAACCCTTAAATCTGGGCTGTGTTTTTTGATTTCCCT
1193 GTTAGAGCGATATCGTCAGACTTAAGATTTGGAGCGGCCCTCCAGTCACA
1194 CACTTTGCAACACCTCAGTCTGTAGTGCACACACACACACACACACACAC
1195 ACACAGCCATTTCGACTTTTCCTCTGGCTCAACACACTAATTATCTGTAA
1196 GTCTCCGGGAGGGAGGAGAGGGAGATTACTGAAAAGAATGTGAAATCACT
1197 CGTAACGGTGCCATGCCTGCTCTATTTTTATCTGTGACTGATAAAAAAAA
1198 CAAAAAAAAATGAGGATCACGAATAATCTTGTCAGCCGCAAGTGGCATCA
1199 ATATCCAACAACCTTGTATGTTTTTCTTTCTTTCTTTTTAATAAGGTGGT
1200 GTGTGTGTGTGTGTGTGTGTNNNNNNNNNNNNNNNNNNNNGGGGGGGGTG
1201 CTGGAGCTGAGGTAGTCCTGTGTTATTCAGAGTGGACAATGTTTTCTCGT
1202 ACTTTTTTTTTTTTTTTTCATTTCCTTTTGTACATGTAGCACAATTGAGCA
1203 TTGCACTTGGGCGCCATACTTTACCTCATGTCAGAGTGTGAAAGGGAGTG
1204 AAAGGTGGAGAGAAAAAAAGTGTATATATGATGCCGTAATGTTAAGAGGA
1205 CTAACTTGTGGTTCAGGCTTTGCTGCTGTTTCAATTGCTGGTAATATACA
1206 AACCTATTTAAGAATAGAATAAGGGGCTATGGGAGGGGAGACAAAATGTG
1207 CCGGCAGGTCATCGTCTTGACAAATACACTGTAAAAAAGAATCTCTGCAA
1208 TTTAGGCCTAAAATGTCTGGACGTCACGTACACGGATGTTCCCGCCTTAA
1209 ACAAATGTTTGGAAATGAATTGTAAGTCAAACTACCACATCCAAATCACG
1210 GCTCAAATGATGAACTGATGCTCGGTTTCATTCTCTATTGACTACTTTGT
1211 CACCCGAGTGATGCTCCCAGCCTGTTTTTTTTTTTTTTTTTAACTCCTGCG
1212 GTGTGTTAGTTTGGAATTACAAGACAAACCTTCCCCAGCCCTTTATTGGG
1213 AACTAAAAAGGAAAAATCCACTTAAACTTGTTTCCTGTACTTTTGGCCAA
1214 TCATAGACCACAGTACCGTGTCATCACTTATTTTTAACATGCCTAATGTA
1215 CTTGTACACCAGTTTGTGAATTCCTGTGAACTGGGGCTGTCACTTTTTTT

1216 TCCTTTTCAATTTAAACTACTGTTTGTATTTAAAGAAAACAAATTAAATA
1217 TTACATTTTTATGTGTACAAAAATGATCTGTGTCCATGCAACAGGCCATG
1218 TATTGTACTACAGCAGGCAGCGTTGGATGGAAAAAAGTAAACTGGACTAT
1219 GACTAGGCACTAAAACATACTGGAATTGAAATAGTATCCCATCACGATTA
1220 TCCCAGTTATGAAAATTCACCACGATAAAAATGTTTGTTTATTGCGATAT
1221 GTGATCTATCGTCCCATCCCTATTGACCTTTCCATTAACACACAATGGAG
1222 TAAACTAGCGAGCTATAATGGAAGATAATCAGTCTTAAAAACGATGTGTG
1223 GAAGAACCCTAGATTTGACACCATGTCTGGGGGGAAAAAAAAAAAAAGGA
1224 AATTGTTGGTAGAAGTAGGACTGTGGCTTGCCCTTTAAAAATGCAACTGC
1225 CTAGTAATGTTAGCTTGACACTCAAATGATGGTGTAGTTTAAGGTTAGGT
1226 TGTATTTGAATCATTTCTAATTAGCTGTAATTTTTTTCAATTTTTTTTTT
1227 TTTTGTAGTGACAGCCCTAATGTGTGTTTGTTTGATCGGAATTTAAACTT
1228 CCTAAGCCAACTTCCTCCTTTCCTGTACTTACATATGCTATGTGCTAAGC
1229 GAGACTTAAGAAGGGTCAGTTGGGCTTAAAACGGGTAATACAAAACAGCT
1230 AGGGCTGTAAAAAGGGTTTTGAACTGCAGCCCCACTGACACCGACAAGTA
1231 TCCTAGGGGTCTTCAAGGGAAATGTTACTTTTATGTAATGTCCAGGCAGT
1232 GTTGATGGTAAATGTAGTCAATTGAAATGTTAAATTCTTTGTTCTTCAGT
1233 TTGTGCTATGTTGACCAAGAAATATTAGCAAGATGTGTTCCACCACCAGT
1234 GCTGTCATTTGACATATAGAGTGATGTAGTTAGTGTCAAGGAAACTACAG
1235 GCTGTTTCCGCTAGGAAATGGTCAAACTGCAGGAATTCATTTTCCTCGCA
1236 CATTTTTGTAGCTTTGATTAGCACCCAGCAGCTAAACTAACTTGCTCACT
1237 GAGCGTGGGAATCCTGGTACATGTAGGCACTCCGAACAGGAGAACAGGAG
1238 AACAGAGCTTTCTCGGTCAGTATAGCCCGCACTGAGGAACTTACTGTTTC
1239 AGTTGACTATATCTTTCATGAATCCTGTCTGGACATCCACATAAAGCATG
1240 GGAAATCTTCCCTTCAAGTGTCCCAAAAGCCACTGTGCACTAGGTTCTGG
1241 AGTGGACAGGAACTTGCGGTTCAGCTTAGGAACCTTCAGTCCTTTAGAGG
1242 CCACAACCTTTGACATTGGAGGCTCCTGAAGTGGGATGCTGCTAGAGTAA
1243 AGAAGTAACTTAACCTGCTCCTACGACAAGATTTATACTTTGTGTTCATC
1244 GACAAATATGAATTGTGGACCGACACTTTTGCCTTTCTTGTTTTAGATTG
1245 CTAAAGTTTTCCGTCATAGCATTGTAGCTACATTTGAAATGTACTGCCTG
1246 CATAAAGTATTCATGGGGCTTGGACTTTACTAATTTGAATATAAAATTGA
1247 ATCACTGTGGATGGCAGATATACAGATGTGATATTCTTCCAAACAAAAAC
1248 AATTATGATTGACCATGTACTCAGAACGTTTTTTTGTTTGACAGCCTTTT
1249 TTCAACAAATTTGTTCAGGATCCTTTCTTGCAACTTCTTATCCCTGATCT
1250 AGGACTGGCATAATACGTATCAAAGTCCTGCTTCACTATCTCCCAGAATT
1251 CTTAGTCACTGAGCAAACTCACCGGTGTATTCAGATCTTTTACTTACAGT
1252 AAATATTCTTTGTTACAAATTAAAGTCAAAACCCTACTTAAGTAAAAAAT
1253 AATAATAATAATAACATCATTATCAGCTAAATGTACTTCTGTAAGTATTT
1254 AAAAAAGTACTCCTTTGATAACAATTTCCTCAATATTGGATAAGCTGACA
1255 GCCTGTGGAACTGGTAGCAATCCATAGACATCTAAAACATGACAGTGGGG
1256 TCCAAATCTGACCCTGTGTTTTTGTAAGAGGTCACCCAAAGGTCTTTAGG
1257 TATTGTATTTCATAAATTTGTCAGCTGCTGTGCACAAATAATTTTAGTAA
1258 AAAGTACAAAGTTTACATAGTAGCATAAAATGGAAATGGAAGAATGTACA
1259 AGTACCTCAAAACTTTACTTCTGGTTGTACTTGGTTACTACACACTACTG
1260 CTGTGTGGTATGCAGTCGTCTTAACTGCTAATAAGAAGGGGGCCTGATGC
1261 AGACCAAGAAAGTTCTCTTCTAAGAGACCTTCTAAGACTACACGAAAGAC
1262 CTTTCTTGTTCAGACAGTTATCTTTTTTGTTACTTTGCACTCGCCTATGT
1263 GTGCCAAGAAGAATTCCTACAATGTAGTGAGTTTATTTTTAAAGTTTAGG
1264 GAATGAATGTTCCGAGTGATAGTGATGCAGGATTGTGGTAGTCAGTGCTG
1265 TCCTGGGTCAGTAAAAACAGATACTGAAATCATCATATTTTGCTTCATGA
1266 TTCTGATTCCTGAACTTTGGGTATCGGCCAATACGGACGACCAACTCTAC
1267 CTGTGACTTTTGATGTTTATGTGCTCTTTCTTAAAGCTGCAGTAGTATAA
1268 AAATAACTTTTTGTCATATTTGCTAACGTTCATTATGTCCTGACAGTAGT
1269 ACCTGGGACAGATAATCTGGCTCCTCCTAGTGCTAAATCCACTGGGCCTG
1270 AATGTGGCCTGAATGATGTGCTTAATGCTTGTCTATGTTGAAAAGACAGC
1271 CACATGAAAACAACCAATCAGAGAAAGGAAAGACAGTTCTTATTATATAA
1272 GTCTTATTCCCAGGGGTCAGATACTGATGCTTTGGCACCGTGGGAATGAG
1273 ACTCAACAATCGACTATCTTCAAGCTTCCAACACAAGCATCGTTTTATCT
1274 TTCTTGGCTCTGATTGGTTGTTTTTGTTCAGGCCCGGTGGATTCTTGCAA
1275 ATACCATTAGGAGCACTAGGAGGAGCCAGAGGAACCTGATTCTTTCACAG
1276 ATTATCTGTTTCATGTTCTACTGTCAGGATATAGTGAAAGTCTCAGCATA

TATGACAAATATTTTTTAATAAAGTTACCTACTGCAGCTTTATTTGACGT
TAAAAGCGGATCCTAATTACAATCCACAGTGATTCAAAAGTCCTTGGAGT
GCCTTCACTTTTTATCGGAACTGTATCTGGATGTAATGTCATCTACATGT
GGGCAGGTGTTCACAGGAACTGTAAAATGAGACGCAAACAAGAAAATGTT
TTTCAATAGTATTAGGTGTTTTAGGTGGATTTTTCCTTTAAGGTCCTCGG
GAAAGCATGATCAGTGTTAACAGAAATGCCTTTTGAGAAAGCGGGGTGGG
GAGGGAATTTTTTGCAGCAAGATATTGGTCTCATAAAGCCAGCATAGAGC
ACCGACCGTGACTGTTTGGGTGGGGTTGGGAGGGTGGGGAGGGCATGGTC
ACAGCAGCAGAACACCAGCAGAGTGAAGCTGAATGGATGGAGCGATTAAC
TGCTATCAGTGTGGGGAATTATGGGCCATCACAACCTGCAGCAAATGTTT
AAGGGACATTGGAATAGAAACTCCTCACTCATACTGTAATACCGTGCTGC
AGTGGTTTGCGGTCTGCACCTTATTGGATCACAAATACCTTTAATTTTTT
TTGTTTGTTTGAGTTTTTTTCTTTTTCTTTTTTTTTTAAAATAATTGTAG
CCCAGTATATCTACAATAAAAGTAATGGTATGGTCATTTATCTTTAGTCC
TGTGGGGTTCATTGAGTTGTTTGCTGATATGACAGTTTGTGATTTTGTGT
AACACTGAAGTCGTGTGCAACATTTTAGAAACGGATAAATTGAATCA

**GCAGTACCGGAGCAGACTTAC = forward primer (LG12:1,244,797 - 1,244,818)**
**AATGATGCTTTCTTTCTGTCC = reverse primer (LG12:1,245,044 - 1,245,064)**
**226 bp**

*** *ehmt2*_European sea bass:**

>dicLab1_dna range=LG9:18388269-18404152 5'pad=0 3'pad=0 strand=+
repeatMasking=none
ATCCGGGAAACATGTCGGCTTCTGAGAGAACGACAAAGGTAGAAAAAAAA
ACCCCACTGCAACACAAAAAATGGCATGCTAGCGAAATGTACGTGCGTTT
AACCTCTTGTAAGCGCATTAGCCGACGTAGCCAAGTCGTGTCACTCAGGC
TGCGGTGTGCCAGGATGCAAAATGTCTCCACTGGTCGATATTAAGAGCTC
TTTGTTGCAAGCTGTGCTACGCTGCTAGCTCACGTTAGCGTGTGAACTCA
CTGTTTTGTGTTGTTCGCTCCGGTGTTGTCGGTGTTTTGGTTATTTTCTC
GAGTTCCTTGACCGTGTGTGTCGCTACATGGTCATCGTCGGATCACGTTG
TGAGGTTTTCTTTTCTTTTTTTTTGGTATGCGTCGCTCAGTTTAGACCGG
TTTTCAAACAGAAACTGTCCTTTTATTTTAATTTTATTTGGTGCACTGTG
ATAATAATGAGGTAATCCCAGGTGATAATAAATAAAAAACCATCTACATC
CCTTCTTTATTTGACAAACTACTGTTGGCGGAAGGATGTTTGGTGTGAAG
TTAAAAGGCATGAATGGGTTGATTGACTGATGGCGGCACTTTGACGTAAC
ATCAGAGCAGATCAGCCATTGGTTGGATGGTTTATTTTGAAGAAGAGAGC
CCGGAAGTGAGTCGCGTAAACGCACCAAAGACAACTTTAGATGAGCTACC
GCATTTCCTCTCATGAAACCCTGCAGGGTTTTAATTGAAAATGAAAACAT
TCAGCATCACACAGCAAAAAAAATAAAAAATGAATGAGAACCTTGACGTC
TAAGACACTCCATTAATGGTGACCACGCTTTGTCAAATGATCCTGCCTTT
CCTCTGTTTATGTAAGTTTCACCAGTACAATACAAGAGGCCATCTCCTTC
ATATATCGAGGCTACAGCCATTTTTCTCCAAGATGAAGCTATCACCCTCC
TGGCAACTGGAGTTGGCTGTTTTGAAACCCTACTAATTTAATGTAACCTA
GAGGATGGTGTCACAAAAAAACAAAAAACAAATGATAAAGTTCAATGACA
GCCAAAACTAAAACCTGAATCACTAAGTTTGTCTTAACTTTCCTGGGAAA
TGTGTTTTCACTAGCGGTGTGTTCTTGGTTGCTGTTTCACCAGTTTTTAA
AATATGCTTGAAAACTACTGCTAATGTATAACTGCTGTGGTTTCAGATAA
TAAAGACTATAAACAAGAGTTATGTCTTTATTAGTTATCCATTGTTATCC
TGTTAAGTTTTATAAAATGTTACGTTACTACTTGACAAGCCAGTGACAGT
TAAATATTTTGTAACATAATATTTCCTGCTATTTGTTGTTTCTCTGCCAG
GAGCCTCCTGAAGGAAATGACTCAGAAACACCAGCAGAGTCATTAGCTGG
ACCAAGTAATGTAAAGAAAGGTATGTCTGCAGCTCATTTATGCAGGGAAA
CATGCCTATTTTTGAAATCTGTACATTTTAGGTATGCAAACAAACTGCTT
GTGAAAACTAACGGGGAATTCTAACTAACTTTTCCAGATAATGCAGCCTC
CACCTCAGCCACTGTTGCCAAGAAGACTGAGCCTATGGGTGGTATGCCAT
CAATGTTGTCATCGCAGCAGCCGGGGAAGGGCGCTTCCAGAGCTGTAGAG
GAGGGAGAGAAATGGTCACCTGCCTCCTCCCCAAACAAACTTGCTGCAGG
TAAGACTCGGACCCAGTCAGCTCTCATCAATGTTGTTAAAGTGGTGACTC

1338 TTTTTTTGAGTTACCTAACTTGATCTTTCTCAGATTTAACAAGGAGATGA
1339 TTCGATCCTGAAGCACAAATTCAGCAGGAAATAATAAAATAAGGCTTGAT
1340 TGATACGATTCTAATAAGCTTTAAGCATTTAAGCTTTTAAAGCATCTTTA
1341 AAGCAATTCACTGTATGTGATTACCAATGGAGACAAAGGGCACGATAGGT
1342 GGGTGTGGGGGTTTGGTCAAAGGCAAGAATACACAATTAGTATACAAAGA
1343 TCAAAGGGGGGCAGAACTGGCTCTCAGATATTACTTGGTCAGTAAGTCTG
1344 CTAAATGTGGATTTTCTTCATCACTGGTTATACCTCCCTCTTACAGGACA
1345 CGCAGCAAAGTCCCTTCCATCAATGTCCTCCTCTTCGTTATCCTCCTCTC
1346 CTTCTACGTCTTCCACGTTGTCCCCTGGCCGAGCAAAGATGAGTGTTTCT
1347 GGGCCTGGTAGCAGTAAATCCGTCCCGGCACCTGCTCCTTCCTCCTCCTC
1348 CTCCTCCTCTTCGTCCTCTTCCTCCTCCTCTTCCTCCTCTGTCACAGCTT
1349 CCTCATCTCCTTCGGTCTCTCCTTCCCCACCCAAGATCCACCGGGCCCGC
1350 AAGACTATGAACAGGCCTCCACCAGGGCAGGTGAGGCAGTCAGCAATAGC
1351 TATATCAGCCAGTAGTGTGAAATGATAAACATCTTTCAGTTATTGACAAA
1352 ACGCACACATTATAGCAGCAGTTCAGTTCTTTGTTGCAACCTTTTTATGA
1353 CCATTTGAGTCACTGACTCTATTTTTGTTTCCCAGATAAGCCACATTGAG
1354 ACACCTGTGACACCTTCAGCATCTTCTGCCTGCCTTTCCTCCGACTCTGA
1355 AACTGGTTAGTGGCTGTAGAATACAGTGACTTTGAGGAAAATGAGCTAGA
1356 GTGCCTTCATTTTTAATTCTGCATTGTCATGTTTCCAATAGTTGCAAAAA
1357 GGAGAAAATTGGGTCATTTATCTGACGGCACACCTGCGAAGTCTGAGGAC
1358 GTTCCAGACAATGTTCAAACTGTGGTAAGATTATGTTTTTGCAGGTATAT
1359 TATTTACATTTTAGTAACTTAATTACATCTGTTGTAAAAGTTGTCTGTTA
1360 CCAATCTCTTTCTTCATTTCTTTCAAAACTCAGGAAGAAACATTATTCCA
1361 TAAAAAAGTGGAACCTGTTACCAAGACTGCACCAGAGAAGAGCAACGGCA
1362 GTGTGGGGAAGTTACAGGAGGAAACAATTCAGGTTTCCAATCCGTCCATG
1363 CAAGATTCAGAAAAGGTGTGAGCCTTAGAATAACTTATTGTCCATTATTG
1364 ACAGTGCAATTAATTCGATGCCTGTGCACACAAGGCCGGTTAAGACACAT
1365 CTGCAGGCAGCCATGTCATTGTGGTTACAGTGGTGCTCCTTCTTTTTTCG
1366 TTTTAGGAAGGAGGTTAGTTTGGAGCCCTGTACAATATCTCTCAAGCATT
1367 TATTTATATATATATATATATATATATATATAGGCCACGATGTTGAGCCGCC
1368 ATGTTTGAACAGTAGTCCGTAACAGACAAACCAAACACTTTCTCTAGACA
1369 GGGCCTTTTAGGAGTTTTGCCGCCATCGTAGTTTCTCCTACACACTTTGA
1370 ATGCGAGTGGTTCCAGTTAGTCACAATCTGCAACTTCACCACTAGATGGC
1371 ACTAAATCCTACACACTGGTCACATTATGAAATCTACCTTAAGTCATCAG
1372 CTGCCCTAATCTCAAAACACTGGCATCGGCCATTGAAGAACCCATATCAG
1373 TCGAACACTGTTGTCCATTAAGGGATCACTTATTGTCAACTAATGTACAT
1374 ATTATTGAGAAAAATGTTATAGCTTGTTCTGCAGGTAATTTTTCTATTGT
1375 TAAACATATTTTGTTTTTCTTTTATAGTTTGAAGATAGTGGGGCCGAAAC
1376 AAAGCAGCACACTGAAGACATAGAGGGGTCAGTGAACATGTCAGATCATG
1377 TGAGTGATTCTATTTTACTAGCTTGATAATTACTTTCTTATCTTGTTACA
1378 GCTACAGTCATCTCTAACTGTCTTATAACATTATAAACACTGTTTAAATC
1379 TGTAGATGTCACTCTAGCCAACTAGTCTTATTAGCCTGACAGTACCAGCC
1380 CACAACTGTTGTATCATCCTGTACTTGCTTGAACATACAAGATGTATTAT
1381 AGATAACCCAATTCTTTTTTTTTCAATAACACAACTCAAACTTGGTTGCT
1382 CGAAAGTCTTTTCTTCCAATTGTCAACATTGAAAAAGATGTTATTCTTAA
1383 ATTGTTTTGTAAATTTCTTATTAACTCTTTACCGAAACTAAAACATGAGA
1384 CAAAATGAGCATTGCTGTTGCAGAATCATGCTTCATTTTCTGGAACTCCA
1385 GAAATAGTTTATGAACTAGTCACGACACAATGATTCACAATGATTTGTCC
1386 CATCAGAAATCTGCTGAGGCTAAATTTATTTTGAGCTGCTAGCCTGCAGA
1387 TATACTGCATAAGGAAAAATATCTTTGAATTCTTAAGGGGCATCTAATGG
1388 GTTTAATCTTAAGCTCAAAGTTGTAGTTGGATTATATTTAAGTACTTGTA
1389 GGGTGAAGATAGTGCTGGCAGAGATGATACTCTACTAGAATACAGCAGTA
1390 GATGCAACACAATGAGCAAAGATTACAAGTAATTAATTTGTGTTTAAAGG
1391 TGCCCTGTGGAGTTCTCTTGTAAACAAACTTGTTTACATTTAGTGTTACC
1392 CCCCCTCCCCCCCGGACGTGTATTTATCTTTAAGGCCTGACACATGAGTG
1393 AGGTGCATTTCCTTCCTCATAAAACATTTGCATAGCTTGGTTTATATCCT
1394 TGTTTGTTAGCTTGCAGTGTTCTTCTTTTTCTTTCCTTTCCTGGTGCATT
1395 ACCACGTAACCAACTGCAGCATAGTGGATTGTGTATAAATAGTGCGTTCT
1396 TGTGCACATTTACGACAAGAAATCCGTCAGACAATAACCACCCAGGATTT
1397 TCACCCTTCTTTCTACCCTTCACAGATGTCACATTTTTATATTCAGACAG
1398 AATTTCTTCCTTGCAGAGTTCAGAGGTTGAAACGCAGAAAGCCGCTCAGA

1399 GTAGAAATGAGAGCGAGGAGTCTTCATGGCCGCAGTCAGACCAGGACAAA
1400 GAGAGAAACACAGTTGATGTGGTGGAGACAGTGGGAGGTGAGTGACGTTA
1401 CCTGGTGGGTTTTCTAAATCTCGTTTCCCATAACAACCATGTTAGATATC
1402 AACAGAAGTATATTTAATGTTGAGGTGAAACAGATCTCGACAACTTAATT
1403 AAGACTGAAGAATAGCAAAGTGCCCTTTACTGTGTCTTAGCCATATCTGA
1404 TTTTTTTTTTTTATGAGAGTCTGAACAGCACAAATCGGATTCTTTCCCTT
1405 CGTATGTGGTCCTAAGTCGGATACATATCTCATGTTTTTCAAAGTGACCT
1406 CAGTCTGAACGGTCAAATTTAAATAATAATGTGAACAACAATGCAAAAAA
1407 ATCGGAATTGAGCATTAAGACCTGCAGTGTGAACATAGCCTAAATAACCC
1408 ATCTAAATCACAGCTGGTGCAACCAGTTGAGGCTGACTAGCCAAGTCACA
1409 TAATAGCTGAATGAAGATCACCTGTGTCTAATCAAGGTGTGATTAGAGAC
1410 TTAAATCCTCTTGCCTGGAAACTTCTTGGCAATTTCCTGCCAAAAAAACA
1411 ACATAATGAAGACAAAGAAACGACAAGACCAGATTATTGAAAAGTACCAA
1412 TCAGGGTACGGGTACAAAAAAATTTCCAGGTTGCTGAATATCTCTCGGAG
1413 TACAGTTAGGACAGTCATCAAAAAATGGAAAGAATATGGTACGGCTGTAA
1414 ATCTGCCGAGAGCAGGTTGTCGTCAAAAACTACACGTCTGTGCAAAAAAG
1415 GCACTCGTGGTGGAGACCGCTACAGAGTCTGTTGGAACTCTGGAGGAGAT
1416 GCAAAGCTGGAGAATGGAGAATTTTTGTAAGGTTGATTTAAAGGTGGTGA
1417 ATATTCGTGCAATTACTTTTATGGTAGAATTTTTATTTTATTTTAACATT
1418 CTACTTCTGTTGATTAGTGATTAATACCTTTTTGATTTAGTGTTGTAAAA
1419 CAATAATAGGTATAAGAGTAGTTATTTGCTTTTTACCTCTATTAACTGCA
1420 TCATTTGCTAGCATATATCTAGCATGTACGATGTATTTTCTAGCATTGGC
1421 AGTGCTTATAGTTTGTCATGTGGTTTGTCCTCAGCTGGTAGGCCCACTGA
1422 GTACACAGAGGTTCCTTTGGGTGCTCTGGACATCGCTGCTGCTGACAGTC
1423 TGACACTTTCTCCTCATCATGGTAGGTTTATGATAGACACAGATGGGTGA
1424 CAAATTAAAGGGAAAAAGAGAATATTTGGCTTTTTTTTACCTTAAAAATT
1425 ACTTATCAAAACAAGTTGCAGGTTGATTTTCTGTTGCAACAATTAATCGT
1426 CTTCAAATCATTTCAGCTGTAATAGAAATCATATTCATTCAGGATTGGTG
1427 ACCATCAATGTCTCCTTCAACACAAAATGGACAGTTACCATAAGACCATA
1428 AACTAACATCATAATCAACTCTCTCTCTTCTCTACAGAAGGAAGCGATGC
1429 CGGAGACACAGAGCGGCTGGAAGAGCTTCCTCTGTGTAGCTGCAGGATGG
1430 AGGCTCCTCGAGTCGACAGCTCCAGCCACCGCGTCAGCAGACAGTGTATG
1431 GCAACTGAGAGCATCAATGGAGAGGTTTGTCTCTGCCTTGTGCTTTTGTT
1432 TGTATTTTGAATTAAGTACCATCATGTGTATTGTTAATGGGTATTTACAT
1433 GTAGTCAAATTTGTAAAGATTTAGTTTTACATTCCTGAATTGTAGAACAA
1434 AAAAATAAATGAAAATAAAAGCTCAAATTAAACCTTCCTTTAAGCCACAT
1435 GTCTAACTCTTGTCTCTCTTGCGCTGTTTGTCAGCTGAGGGCTTGTACCA
1436 GTCATACGATAAAAGGGGAGACCATGCGGCCATCAAGTCGGGTGTCTCTC
1437 ATGGTGCTTTGTGACGTCCATCGCTCACACATGGTCAAACACCACTGCTG
1438 TCCTGGATGTGGATACTTCTGCATAGCGGTGAGGGTTTCAATCACCAAAT
1439 TGTCATGATTAGAAATACATAGTGTATTTAATGTTGTTGAGTTGGTACAT
1440 TGGGACATTGTTTCATGGCTGTACATGTTTTCCAATTTTATTGATGCTTT
1441 AGAGTTTTACAAGCTATAACTTTAATGTAGTTTAAAATTATTGTTAATGC
1442 TTTTTCCTCCTCAATATCCTACTTTCTCTCCAATATCTTACTTTTAATGG
1443 TCTTCCTCCTTCTGCATATCCTACTTTCTCGTCCTCAATGCCCGACCTTC
1444 TTCTCCTCTTTCAGGGCACGTTTCTTGAGTGTTGCCCAGACCAGCGCATC
1445 GCTCACCGTTTCCACCGGGGTTGTGTGACGGTGCTGGGCGGCGGGCGCAG
1446 CAGAGCAAACGGTGGCGGTATGCTTTTCTGTCCCCACTGCGGTGAAGATG
1447 CCTCCGAGGCCCAGGAGGTCACCATCCCCTCCTTCAGCTCGGCCACAACC
1448 TCCGCAACCACTGTCGTCACCATGTCGGCCTCGTCCACCACCACCCCATC
1449 TCTGCCGCCGTCCACCCCCTCAGCGCCCTCTCTTACAGCCTCAACGGGCG
1450 GAATGAAGGACGGGAAAATGCCCGAAAGACCTGTCAGGTAAGATTTGTGA
1451 ATGTTTTTGTGGATGACTTGTGAGAGAATTATTGGCTCCTGTAATCTATA
1452 TAAATAATGTTTTTCTTACTTGCGTTCTCCTTCACCACCAGCGCACGTAT
1453 GCGTAGCCATGGTTTGATAAAACTGGCAGTGGAGCAGCAGCCCCCGCAAC
1454 CTGTAGCTTCTGCTGCAACTGTGGCCACTGTGCCCCCAGCAGAGGAGGGT
1455 GTGGACAGCGTGGGGCCCTCTCTCTGTATGCCAAATGGGAAACCCGTCAG
1456 CCCCAGTGCTCTTCCACCTGGGCCCAGCAGGGCGTCGCTACAGAAGGCCA
1457 TTCTCACACAGGAAACTGAGAGGTTGGCGTTTGGATTTAGATTTGACTAT
1458 TTTCAGTCACAGTAGGTTAAAGGATTGAACAGAGGACTAGATACAGTAAT
1459 TGAAATATGTAAGGGTATTGTCTAAATCAGTGGTTCTCAACCTGTTTGGT

1460 GTCAAGGATCCCCCAAATAGTCTCAGAGATCCCCCTTTCATAGTATTTAG
1461 TTATTCGATAACCGTCTGTACTGTAGATTAACAGATAAACTGTGAAGATA
1462 GTGATTTGTCATTATTTTACAATTTCTCATTGGGCTAACTTCTAACCAAT
1463 GAAATAAAAGTGAAATGAAATTATTTCCCATTTTTCTGGGTAACCCAATG
1464 AACCCCCTCAAGGTCTCCTGTTGGATGTTTTATGTTTAGTTTCGACATTG
1465 TTTAAATATGAGCTTTCAATTGAAAGTCTAATTTGTTTTCTTACAGTACG
1466 TGCAACGAGTAAGTAATTTTTGTGCGGCAGAACATTTTTTATTTTATTTT
1467 TTGATGAAGTTTTTGTGCTAGCAGTCCAAAACTCTATACTAAAACTACTA
1468 TACAATATCTGATATGCAGCAGCGGACAATTTACAAATAAAATCTGCTCA
1469 TGAATCCTAGTAAAAGCATCTCCACTATATCTGTTGAAATCTGCAGCCAC
1470 AACAAAATTGCATGTTGCAGGGAAATTCTCTAAGCAAGACAGAGTATTCC
1471 AGTTTTGTTTGCTTTAGCTCTTCCAAGGGAAATTACATAGCTTCCAGTGT
1472 ACCAGAGAACATCAGTCAAGTTGAGACCAAATTGTTGTTTCTGATTTGCC
1473 AGGAAGAAGAAACTGAGGTTCCACCCCCGCCAGCTTTATCCTGCTGCTAA
1474 GCAAGGAGAGGTGCAGAGAGTTCTGCTCATGCTGAGTGAGTGAATACCTT
1475 GCACTTTTCATTTATGGCTGAGTGAATAAATATAAAAAAGCTCCAGGATA
1476 TATCTTATTTTGAAAGTACTGTCTGGGTTTGATCACACACAGAGAAGTGT
1477 TGACCATTTGTGCACGTCCTCTTGGCTGCTAATCCCAATATGAGATCATG
1478 GTAAATTTATACAGATCTGTGCATTAAAAGTGTGTGTGTGTCTGTGTGTA
1479 TGTATCACAGTGGAGGGCATAGATCCAACGTACCAGCCTGACTCTCAGAA
1480 CAGACGCTCTGCTCTACATGCTGCAGCTCAGAGAGGTCTGCTGGAAGTCT
1481 GCTACATGCTCGTACAGGTGAGAAGTCCAATATTAGCATCTGTCTTGTAG
1482 GTTTCTTTTCTCTGATTAGTACACATACTGTAAAGAGGTTATAATCACTG
1483 TGAGGGGTTGCTCACAGTAATGAACCTACAGAGAATTATCACCTTAAGCT
1484 GCAGGTCATTGTTTAGCTGTCTGGCCCGCAGCTTTACTGTCTTGGTTCAC
1485 TCTCCCTGCTCATTTTTGGCCACAGCAGGCAGTTGGGTGAAAAGGTCTAA
1486 AAGCCCACCCTACAGTACCTGCTCAGCACAAAACTGCGGACAGAAACAGT
1487 TAGAGACTAGCTGATGAACATAGTGGAGCAATTGGCGCCTAAAGAGTCAG
1488 ATATTTCCCGTCATTTTTATGTGTCTCTGCTGCCACAAGTGGCCAGAAAA
1489 AGCTGTTATTTTAGCATCAGTTTTAAAATAATGTGAACAAAACATTGGAA
1490 GATAATACATTGGATCTAAATCTGAGGGTCACTGCTGTAGTGTTAATTGC
1491 AAAAACTATGACGACTATGATGTGTTATGTAAATTACTTTTTTTTCCCAA
1492 GACGATGACAAGCTAAAAATAGAACTTTAATTAATAATAGCTATAAGGAA
1493 ATGTATGTTTTTTCAGTGACAAGACAGGACTAAAATGTTAGCAGTTGAAT
1494 CAGAAATTCAAAAGAATGCATTTTTTCCCCAATAACTAAGAGACCCGACA
1495 CCCGAACCCGCCAGTTTTATGCATTGTAGTAGCACAATTGTCAGGACTAA
1496 GCGCAGTGCTGCTCTCCAAGGGACTGGTCCTGCTGCCAGGAGCAGCTAGG
1497 GAGCTGTCCAGAGCACTGTGTCTCTTGACCCGCCCGTCCAAACCTGTGAT
1498 ATTCTCTAGTAAGTTGAGCCGAACCCGCCCCACCTACGGGTCCCGCATCA
1499 CTATTACACAAGTAAAAGTGTTAACTACGAGAATGAAATGTTGCTTTACG
1500 AATTTCAACATTACTGTCACCTTGATTCTAATTTCCAATACCTGTATTAG
1501 CATAATTTAATTAGTTTAAAAATGGCTAAAATTGTGACTACCACTAATAA
1502 GATTGACAGCTATATAGATTTAGCCTAGTCTACAAAAATGCTTAACACTG
1503 CACTGGTGACCTTATTTAAAGCAAAATGCATCAGAGCTAATTATTCTGTG
1504 TGTTTATTTTCCCTCTTAAAATCTCTCCTGGTCTTGTGCGTTAAATTAAA
1505 GATTAGATTTGTTTGTGTACTGACAGGCTGGTGCTCAAGTGGATGCCCAG
1506 GACAAAGACCTGAGGACCCCTCTGTTGGAGGCGATCATCAACAATCACAT
1507 TGAGGTGGCTCACTACCTGGTCCAGAATGGCGCCTGTGTCTATCATGTTG
1508 TGAGTGTCTTTTTAGGTCCTAAAATAAAAACAGAAATGTGAACTTTGAGG
1509 GGTTTGGTTGATATGGATTTCAATCCTTGTTAATGTCCTTCAGGAGGAGG
1510 ATGGATATACTGGCCTCCACCACGCAGCCAAGCTTGGAAACCTGGAAACT
1511 GTTAACATGCTTCTGGAGACGGGGCAGGTGGATGTAAATGCACAGGTAAA
1512 GGATGAGTCAAAATGTCAGTTATATAGGGACATTATTATAGGTCCTTATA
1513 AGGTTGTTTATCTCATTTTATTTACATAATTTCTTTCAGCTATGTGCAGC
1514 TGATAGGGTTTTGTTTTTTAGGTTAAGAGATAGCTACAATCAACCACTGT
1515 ACCATTCTCTGATATTTATGTGATACATGTGATCATTTAAGCAAAGTGTA
1516 TTGTATCTGTCTTACTGGCGGTGGATCACACAGACTGATACGAGCCCACT
1517 GTGACCTCCATGTGTTTCTTCCTGTTGGTTCTAGGACAGCGGTGGCTGGA
1518 CGCCGATCATCTGGGCTGCAGAGCACAAACACGTAGACGTGATAAAAGCA
1519 CTGCTGAACAGAGGAGCTGATGTCACCATTAATGATAAAGTAAGTCTCTA
1520 CTAGTGAACACTGAGGTTTATTAAATGTGTTGCATAAAAAAATTTCATCT

1521 ACATGAATAATATACATTTTATTTCTATAGAATTTTGCTATTATCACAAT
1522 GTTATTTCATAAATACTGTTAAATATCTTTTCCTTTTCTGGACAATGTTG
1523 TTTTTTGTAAAATCTTATTTTAACTGAAGGAAGAAAACATTCTTTTGTCA
1524 TAATATCGATAATATTGTAATACAATCAACAGTAATGCTTTGATTACTGA
1525 AGAATATTTTTTACTGAACAATTAATTATACTTCAGTAAGAAATCACTAC
1526 ATTTACAACACTGAAGATAATGTTTAAAAGGATAAAAATAAATGTTTCAA
1527 ATACGAATCGGGGGGGGGGGGGCACTTTGCACCTCTACTGTCCTGTATCA
1528 ACTGGTGATAGAGAAAAAATGTATGTAGTTGCATGAAAATATTTCAGGTT
1529 ATTACTTTAAACAGTCCAATCTTTCCCTTCAGGAGCTGAATGTGTGTCTC
1530 CACTGGGCGGCGTATGCAGGCAATGTGGATATAGCAGAGCTGGTGTTGAA
1531 CTCCGGCTGCTCCCTCACGTCAGTTAACATGCACGGAGACACGCCGCTCC
1532 ACATCGCCGCCAGAGAGGGCTACTTGGAGTGTGTTACGTGAGTCAATATT
1533 AATCACAAGGTGTAGCCTTATTTAATGTGAGTCACACAGTGTCATTTTAC
1534 TTTCCCAGCAACAAAAGTGAGGAACGCATGAAATTTGGTTGTTTTTATTT
1535 ATTACACACAGACATGATATACATATCTGTGGATACACTGCAAACAGGAA
1536 TTGCTAATAGCTGATTTGGGATATTTTTAATAGTGCTAATTTACCAAAAT
1537 GTAATTGAATAATTGTTTAATTGTTATAGTTTTAACTGGACTGTGGACTT
1538 TTTTTCTGCAGGTTGTTCCTCTCCAGAGGGGCCGACATCGACATTATGAA
1539 CAGGGAGGGAGACACGCCTCTCACCCTCGCACGGGCCGACACGCCGGTGT
1540 GGGTGGCACTCCAGATCAACAGGAAGCTAAGAAGAGGAATAACCAATCGC
1541 ATGCTTCGGACTGAAAGGATAATTTGCAGGTAGGTGTTGATTTAATGGCA
1542 TATAGCAGTGTAATTGCAGATTGCACCTCCATCGCTTCACAGCCTTGTCT
1543 TCAAATTAAAAACGTGTTGTTCTTAACCAGATAATTTTTCCTCTTGCTGT
1544 TTTTCTGATGTCCATCTTCCTGCTTTCCGTTCCAGGGACGTTGCACAGGG
1545 CTATGAGAACGTACCGATTCCTTGTGTGAATGCAGTGGATGACGAGGGCT
1546 GTCCTTCAGACTACAAATATGTTTCAGAAAACTGTGAAACTTCAGCAATG
1547 AACATAGACCGCAATATTACACACTTACAGGTAAAAAATATATTGTTTTG
1548 TCTTTCTGTTCACACAATTGTATTATCTGCAATGAAAAATACATCTCATG
1549 CCTTTTTAATTATCTTTACTGCAGCACTGTAGCTGCACTGATGACTGCTC
1550 ATCTAGTAACTGCCTCTGCGGACAGCTCAGTATCCGCTGCTGGTATGACA
1551 AGGTAAGGTGAAATGTTGCTTTACAGCCACACCTTTAAAATCAGCTGCTC
1552 AGAAAGCTTATTGGGATATAAAGAAGTGAAACAATCATTCAACAAAAAAT
1553 GACAAGCTTGTGTCAAGAATATCTTTTTTCCATTTGGTCAATAAATATCT
1554 GTATAATCTTTAAGTATTAGCAGTCATTTTTAAAGAGGTTTAGTTTGAAA
1555 GTCAGTGAGATCTACAAGAGTCAGAGTCAATGACTAGAATTAATTAATTA
1556 CAGGACGAACTAAAGGTGTGAAATTATTCAACTCAACTTGTTCTGTAATT
1557 TTTATAGGACCAGCGACTGCTCCAGGAGTTCAACAAAATTGAACCTCCAC
1558 TTATATTTGAATGCAACATGGCGTGTTCCTGCTACCGAACATGCAAGAAC
1559 AGGGTGGTACAAGCAGGCATCAAGTAAGAGCTTCATATATAACTCCTCTT
1560 AACATAATATTTCTCAACCAAAACCGCAGCTTTTTTAACTAATTATACAG
1561 CAACAGATTTCACTAATGAGCAAACGTTAAATTACCTTGTGAAGTTACTG
1562 GCTGGGAGGAAACCTCAAACAGTCTGACCCCCTGATAATTTCCCCCTCAG
1563 AGTTCGTCTTCAGCTGTACAGGACAGAGAAGATGGGCTGGGGAGTCCGAG
1564 CTCTGCAGGATATTCCCCAGGGAAGCTTCATCTGCGAGTAAGAGAGACCT
1565 GTCCACAAGGCACACACAACAGGCTGCTTTTATGGGAAAAGTCAGAGTTT
1566 AGAAATGACGTTTCTTCCCTGTCCACACAGATATGTTGGGGAGCTGATCT
1567 CCGATGCAGAGGCAGATGTTAGAGAAGATGACTCCTACCTGTTTGACCTG
1568 GACAACAAGGTGAACCTTTTTAAAATGTGTTTTGTTTTTATGTTTCTGCT
1569 AATATAGAAACAGATAAAACATGAGAGAGACAGTAGATGGCATATTTCCA
1570 GTATATCCAATAATTAATAACTATTCAAGTAAATAACTAGCCAGACCTTA
1571 ACCCATGCACAAAGTACACAACTGCTGTGCTAGACAACAACTCCCAGTGT
1572 TTTAGCTTGCAGCAGACTTTTAAGGTAACATTTTCTTGGGAAACTGCAGT
1573 TTTAAAACTCAGTCAGGTGGCAAATACTTCTTCATTATGTTACTTGTAAG
1574 TTTGGGGTAAAAAAAAAAAAACTTCCTCTAATCGTCCAGTTTCTGCAAGT
1575 TTCCCCAGCCTTTTTGATATTTCATCACGTCCTGAATTTTGAGACCCAGA
1576 CAAACAGACCATTCAAGACTATCCAAAGAAGTTACCAACCTTTGCAGGCC
1577 TTTAAAATAAGTTTTACATTGTGTGCTATGGAGTCAAATTTGATCTATCT
1578 GATACTATTTTGTTGTGAAATACTGAACTGAAAAAAACAAAAAAACGAAA
1579 AGTTGTATTCAATGGTTATCTGTTGCTGTATTTTGAAAGAGATGTTATAA
1580 ATATGCAGATTAAAACATTTTCCATCATTAAAATAGCAAAGTTGGTGCAT
1581 TATGAAATATAAGCAGTGGTATGCAGCATTTATTTCTTTATTTGAATAAA

ACTATATATAACAAGTGTCTGCAGCCATGCTAGCAGCTCTTTGACACCGT
ATTTGGATATAGCTGACCTTTAAGCTCAATGCTAAAGTCTAACATGCTGA
TGCCAAGCAGGTGTAATGTTTAGTATCTTAGTTTAGTGTGTTCGCATGCT
GGCATTTGCTTATTAGCAGTAAACACAAAGTAAAGCTAAGGCTGATGGAA
ATGTCGTTAGTTTTGCAGGTATTTCTTCATAAAACAAAGTATTGGACAAA
TTACCATTTTGACCTGATGATGGCGTTAGAGGAAAAGTTAAAAGATCACA
ATTATCCTGACGGGGGGGGGGGACGTGAATGTCTGCTACTAGTTTTCATC
GCAATCCATCCATAGAGCTAAAAACTATTTAAAGTCGCACAGTATCAATA
TAAGAAAAGGCAGAACAAGCCAAACATGAAGATGATAAAACAACCACAGT
GTATAATAGTGACACTAATGTAGTCGAATAAATCTGACCATGTTTGTCAG
ATATCAGGATCTGTTTATCGTGCATAATCCGTGTGAATAGCTTTAGCTCT
CCAACACATTTTTTCCTCTTGTGTAAAGGACGGGGAGGTGTATTGTATCG
ACGCCCGCTACTATGGAAACATCAGCCGCTTTATCAACCACCTGTGTGAC
CCAAACCTCATCCCAGTGCGTGTGTTCATGCTGCACCAGGACTTGAGGTT
CCCCCGCATCGCCTTCTTCAGCTCCAGGGACATCCTCAGCGGGCAAGAGC
TAGGGTGAGAAGTTGAGACGTTTACGGTCACCAGTTTATTTCTTTCTTTT
CCTCCTTTTTATAGCCTCCTATCTGCCTTATTCATCCTCTTTTCTTTCTT
TAATCCAGTATTTTGCATTGAATCATCTTGTTTTGGGCAGAGAGGCATGA
AAGTTTTTTATTTATATATGTGCCTGAAGCCCTTTCCACTATACAAACAC
TTTCAACTTGACCTGGCCTTGTCTGTTTCATTAGGTTCGACTATGGAGAC
CGCTTTTGGGACATTAAGAGCAAGTATTTCACCTGTCAGTGTGGATCAGA
GAAATGCAAACACTCAGCTGAGGCCATCGCCTTGGAGCAGAACAGACTGG
CTCGACTGGAAGCTTGTCCAGAATCGGGAGCTGACTGTGGGATGACCATG
CTGGGAAACTCTTAAAAACATTTACCCACGGGGGCCTTTGGATTAACCAC
ATCCATTTCACATGTACTCGCAGTGGAAATACACCTTGAGGGTTTCATCA
TGTTAAAAATTCTGTTC**TGTGTTTGATGCATGGTGCT**GTTTTTTTTTCTA
CTTGAAATGTCCCTTTTTCTTTTCATACTTGCCACCTCTTTTAAAGTTGA
TCATTTTGGATATGGGCCTTAACATGTTTTAAACTGTGGGTATTTTGAAG
GAAACAGGTGATGATCCTGTACATACAGTATGTTTACTGTATATTTTAAG
ATCTCCTTT**TGCCCTGACACATGAAGTCT**GTGCATTAGGAGTCTGCCATA
ATGCAACTGTCATAAATATATATTTTCTGTCGTGCCAACAACCGCAATGC
ATTTTTACACAGTACAGTAATGGGGTCAACAGTTAGCCCATACTGGACTC
AATAAGGACCTGAGGGATAGCGTTGTTTTTGGATGATGGCGTTAGCTTTA
TTTTCTTTATTTTTGTACGTATTGTTCTTTCTTTATCACATTTAGTGTAA
TAAATTAGCTCATGAGTTGATCTTTGGGCTCTTGGATTAAGTATGACATT
CTCTCTTTAAAAATCTGCGCTCACTGGCTATCAATGGACCTTTTTCACAG
CAGCTATTGTGACATGAAACAGTACATTAAACACAGGTGTTACATATGAT
ACCGGTTAAGGTTCTGTTGCATTCAGGTAAAACAGAGTCAGGATGCTGCT
ATTGTGCATGTTGGCTCACTGGAACGGCTCTGCC

**TGTGTTTGATGCATGGTGCT = forward primer (LG9:18,403,519 - 18,403,539)**
**TGCCCTGACACATGAAGTCT = reverse primer (LG9:18,403,711 - 18,403,731)**
**172 bp**

*** *hdac11*_European sea bass:**

>dicLab1_dna range=LG13:22906518-22909221 5'pad=0 3'pad=0 strand=-
repeatMasking=none
CACACATCCGTCAACGAACTGGACACCTTCAGCGTGTTTGTGGTGTCATT
TCATAACATCGAACAACGCAGCACTTAGCACCCCCCTGTTTTATGTAAGT
GAGTCATTTGTTAGCATTAAGATCAGTGAGAGAAATGACCTACATTAAAC
CGATCTTTTCTAAAAGACCTCGCTTAAAGGTCGCACGGCTGCTCGGTGCA
GCTTTCAGCTCCTGCAGATGTTTCCATGCAGAACTAGTAAGAACTTGTGT
ATTGTTGTGAACTTTACACACACATTGCATTGCATTAAGACTGACCTCAC
AGTCAGCCTTTTCACCTCGCAGGTGGGACAGGAATCCAGCGGACTTCCAA
TAGTTCATCACAGCAAGTATGTGTGTGACCTCCCACCCAACCACAGGTTC
CCTATGGGCAAGTTCTCCCGGGTTCTCCACTTTCTGATCAAAGATCAAGT
CATTACAGAGAAACAGGTGAGTGGCTCATTGACATCATCAACTAAATAAC
ACAATTGAGATTAAAACCTTTGTTATTTAGTGCTGAAATGTCATCAATAT
AATCGATTAGTCAACTGATTATTATGATGATTTGATTATAAAGTTTGATA
TCACTCTAAATCGATTATTTTATATTTTTTTATTTTATTTATATTTTTTG

GTAGGACAAACCATAAAGACATTACCCTTTGCACTAGTTATTTTTGAAGA
ACAAGAGATGAATCACTGATAAAAACTTCTGATTGATTAATTGATAGTGA
AAAGCATTGTTAGTTGCAGGAATACT**ACAGCACTACTGGAAGCACT**TTCA
CATTTTCCTGTTAATGTGAATTTCCTTCCTCGCAGTAGGTGTGGGTCCCT
GAAATTGCCTCTATAGATTTACTGAGCTGTGTGCACACGGAGGAATACCT
GAGCAACTTCATAAATGGGAAAATAAATGAGCAGGAGCAAAGGAGGACAG
GTTTCCCCTGGAGCGAAGGCATAGTGAGACGCTGTCGATATGA**AACGGGT**
**GAGAAGAACGTCT**CTCTTGATGACTTAACAGCTGAACAGCCTGAAAAAAA
AACCCTGCACAAGCTTCTCACCTTCTCTCTTGTTGACAGGTGGGACTGTT
CTAGCTGCCGAGGTAGCTCTACAGAGGGGAATGGCCTGCAGCACTGCAGG
AGGAACCCATCATGCATTTCCAAGCTATGGTTCGGGGTATTGTCTCCTCA
ATGACTTGGCAGTTGCAGCCAAATACCTGATCTCCTCACCCAAAAGGAAG
GTTCTGATTGTGGATCTAGACGTGCATCAGGTAGCGTCAAAGCTGTCTCT
GCTACTAATAATGCCGTGGGGTTATACCAATATGTCCTAATTACAGCATG
TGTCTTTGTTCCTTGTTGATTAACCCAGCTTTTTGTCATTAGTAACATCT
TTTGCATCCAACAGGGTGATGGCACTGCTTTCATTTTTAAAGATGAGTCG
TGTGTGTTTACGTTCTCGGTGCATTGTGGGAAAAACTTCCCCCTCCGTAA
ACAACAGAGTGACCTAGATATCAGCGTGGAGGATGGGCTGGAAGACAAGG
AGTATCTCTCCACAGGCACGTTAACAAAAGGCCATTATAATACACGCAGG
ATGTTTAGATCCAAACAGAGTTGCAGTCTCAATTTCACTACTGAAGTATT
TTAGACATTTATACATACATTTCTGTATTTCTGTTTTCAGAAGAAGCTTG
ATGTGAACAAGCTAAATGAAAATCACAACAGAAAACTATTGTAATTAGTA
GTGTTAACAGACATTTTAATGAAGAGTTTGTTTCCAACAGTGGAGGCTCA
CCTTCCCTGGGTGCTGGAGACATTTCGTCCAGACCTGGTCCTGTATGATG
CAGGGGTCGACCCTCATTGGGAAGATGAACTCGGGAGGCTCAGTCTGACT
GACCAAGGTCAGTGATAACTTTGACACCAAACTATCTCCACCTACAAATC
AGATCTTGACTGTTTTTGAATGACTGTGGTGTCTGCCAGGGCTGTATCAG
AGAGATCTCTACGTGATGAAGACCGTGGTGAGTAGAGGCGTTCCTGTCGC
TGCCGTTATTGGAGGAGGATACTCGAGAGACATTGATAAACTGGCTATCA
GACACTCCATTGTCCACAGAGCAGCGACTCAGGTACAGGACACATAAGTG
TTGTTGGATGACTCATCATTTACATCAACAGTTTAACTGAATGTTCTTCA
TATTTCAGGTTTGGAGGGAGTGTGGGATGTAAAACCTTTATGCTGTATAT
CAAGAGGATCCTCAGAGCCTAACTGATACTTGACCTTTAGGAGCCAATGA
CAAGATTGATATGTGGGAATAAAGACATCTGGTTATGCTACACAAATGCT
TGTTTTTATACAGTATATTTATATGTACATGCAACAGTTTTTTTCAAAAA
TTATGAAAAATATATTGGGAGACAGTGGAGCCTTAACAAATTCCACCAAC
TTCTCAGAATATCGTAGAATATTTTATTCTGCTGGACAAATGACGTAATG
TAACTAACCTTTTTTGGTATATTGGACAGGATATTTTTAAATGGAGAGTT
GGTGTATGTTTGCTGAAGGGTGATTTAAGAAGACATTCAACTTTGTTTAC
CTCATCAAGATGCTGCATCTCTGCAGTAGTAACTATCAGTACAACTATTA
AATATTGTGTAATAGTGTGTGGTTGTCACTGAATAAAGCTTTACTAAATA
ATTA

**ACAGCACTACTGGAAGCACT = forward primer (LG13:22,907,294 - 22,907,314)**
**AACGGGTGAGAAGAACGTCT = reverse primer (LG12:22,907,511 – 22,907,531)**
**197 bp**

*** *pcgf2*_European sea bass:**

>dicLab1_dna range=LG7:5304022-5313219 5'pad=0 3'pad=0 strand=-
repeatMasking=none
GGGTTGTGGTGCCCGGTCGGACGGCGGAGCTCCGGGGAAATGCAAACGCT
CTTTGTTGGTCCGCTGGTCGAGGAGCTGAGCTGATGCACAAGATGAGGCC
ACACAGGGGCGATTCAATAGTCGGCTGCTGTGTACCACCACACAGGCTGC
ATTTAGCTGCACGGCTCCTGCTCTCCTCACGTTATTTACCGCTACAGCAT
GTCATCCCGGTTGTTTTTTGTTGGCCATCAGCTCTCTTTGTTGTGCTTTC
ACAAGCTGCTGTCCCAAGGACGAGCTGCAGACATTGTCTGCGGGAGCAGC
CACTCATTGACCATATGAGAAAGGACTAATAGTCTCAACAAGCATTGTGC
ACATTTACACGGACTCCTGTGTGTTGCAGTGTAACAAGAAGCTCTCATGT
TGTTGTCTGTTTCCTCTGCAGATCTGTCCAGATGGATAAAATGCAGCCCA
ACCGGATTAAAATCACAGATCTGAATCCACAACTCACATGTCCACTGTGC

GCTGGGTACTTAATTGATGCTACAACCATCGTAGAGTGCCTGCACTCCTG
TAAGTCCCTCACATGAAAGCATCACCTTACATGCATGCACAGTGTTACCA
TTAACCCCAGTGTGACCTTAGCATTACAATCCTCTGTATATTGAGCAGAT
CTCTCCTGCTCATGTTGGAGGTGAGCATGATGTTGAGTCATATACTGTGA
CTCCATCTTAATGATCATTTTTTAAACCAGTCCTGTGGGAGGTTTACTTC
AGGGAATGTCTGTGTGCACAATAACAATGTAATGCATGTGAAAGCATTTG
TGTTGACAGTGTGTGAGGTTCAGCTCCATGGTAGCTGTGGAGGCTGGTGA
TGGACTGTTAAGGGGTTTCAGTCCTTCGCTTAGCAAAAATGTAACAAAAG
CAGCACTGAATTATTCTGTTATTTTGAATTGCAAGTGTCCACTCTGTGAG
GTGAACACACAGTTGTGTTTCAGCATCTGTCTGTGTCCTCTCTCTCTGCA
CAGTTTGTAAAACTTGTATCGTTGCCTTCCTGGAGACAAATAAGTTCTGC
CCTCGATGTGACGTCCAGGTTCACAAAACGTGTCCACAGCTCAGCATCAG
GTCAGTTATGATGCTTTTATAGACATCGGCTGTTTTTAAAAACACTTTAA
AAAAACATGCGCTTACCAACATGAAAACCTTTCTTTTCCAGAGCGGACAA
AACTCTACAAGATATTGTTTATAAGCTTGTTCCAGGGTTATTCAAAGGTA
AGACCATTGTTTTGTGAGGCTGTAGAGCAACATTTTCCCATTTTATGATT
CATTTCTTCACCAATGGATGCTCATTTTCCCTCGTGTGCTGCTTTCAGAT
GAGATGAAACGGAGGCGGGACTTCTATGCAGAGAATCGTGTGCTGGAACC
GGGGGAAGTGGTGGAGACATTTAACATAGCAGAAGATGAAATCATCAGTC
TGTCCATACAGTTCTATGAGAGGAACAAGTGAGTCAGTTTTTTCTCATTT
AAAAAAAAACAACACAAAAACACTGAATTTTTCATGTTCGGGTCTGTTTA
CTGGAAACTTGCTGCTGAGACAAAAACAACGAAACACAATTATTTGATCA
TTATCAATAGAGTGGAGTTACTTAAATATGCAAGTAAGGCCCATATTAAA
TAGAAGTGAAATTAGAGCGAGGCTGTTTGAACCTGCTGTAAGTCTCTCTG
AATATGGTTTTCTGTGTGCGTGGAAAGAATGGTTAATTATTGTTTAACAC
CTTTTCTTTTTGTTGAATATTTGCCTGCAGAAATAATGAGAGGCAGCGCT
CAGAGATGGAAGGAGACAAGGTAAAGCTGGATTTTAGATAAAAAAGCAAA
GTTGGGTAAAGCAGTAGTTGAAGCCTAATGTGTTTTTCTGGTCTTCACCC
TGGATTTACGCCCCCCCCTCTCCACTCTCCTCTCTGTTCTCAGTCCAATG
GTAAACGCTTCCTACAGTGCCCAGCAGCCATGTCCGTCATGCACTTAGCG
AAGTTCCTCCGAAGCAAGATGGATATTCCCAACAACTATCGGGTAATTAG
ACACTCTCGCTGTATTGATTACGTAGCATACCTCAGGGTTTAGACGCAGT
AATATAAATTCTTGGTGTAAAATGACTTTTAACTCTAAAGTAACGTAATC
CCAGTCGTGGCTGGGTCATATGAGGTTGCATTCATTTGTCAGCTGTCAAC
ACAGGATGATCACAAACAGAGCTCCAAACAGACAAAATCATATGTGCCCA
AACTGAGGGACATTGCTCGAAAAACGAATTAAAGCTATTTTTAACATTGG
ATCAAATGACTGGGTTTGCAAGTGATCCCTTACACATTAATTACACAGAG
CATTTTAGTGTTTTTTAGCGCATTGTTTTGATTTTACAGCCTGTAACTTC
ACTGTTTTTTTTCACTTCACTGCTGTCATCGGCCTTGTTTTCAGCTGTAA
CAGGCACACTTCCTGCTGAGCATCCAAAGGAAAGTCAGCCAACATATTGG
AGCATTGAGAAGTTTAAGACTTTAAACGGACAGATATTTTTCTCAGGAGT
AAAGAAGAGTAAAAAGAGAGTAAATATTGGATTTTTATTCATCAGGTAAA
CACAGAAAATACCCCCAATAAATGCTAATGTTGCTCCATATCTGCTGGAT
GTGTAAATACTGGTAGGCAACTGTTGTAACAACATTGCATTTTTGCTATC
CCTGTTATGTTTACATCTTCTATTTTTATATTGATGTTCTTAATGAGCAT
TAATGAATGTGCTCTGCCCCTATCAAACAAACAAACAAATAAAATTCTGT
GGCAGCGTCGCAGTGAGCCAGCATTCACAGTAATGGTCATTATAATTATT
TGATTAATTGCACCTATGCTTTTACTGCTTTCACTAGTCAAAATGTCTGC
TATAAAAAAAAAAAAATATGATCCATCTTTTGCTAATTTGTCAGTATTCA
ACAATTGTTGGGAATGTTAACATTATTCCAAGATCTGTACCACCACTGCT
AGTTAGCAGCTTTCTAACAAGTGAAACCACATACAAAGACCGTATAGCTT
TTAATGAGGTAAATCGTTTGTTTTTGTGTTAATAACATTGTTTTTTATTT
AAATGTAACTGGCCGTGATAGTGGCAGCCATCTTGCCACTGTTTGTTTGA
TACTTTGAGTTTTTACCAAAGGGGCAGATTTTGTAGCTGGCTGAAATTCT
TACTAGCTATTAATAATGGTCTGTACGGTATGATGTGAGTGTGGCATTAG
ACACAGATGCACTGTCAAATCCTAATCCGGTGTAGACATCAAGTATGTCA
TTGCAGCTACATAGCATGCATGGCAAACAATGGTTATAATGTAGCTGTAG
CAGGACCCGGTCCCTACGAGCCATTCAGCAGCAAGCAAGATTATACAACA
AGGGTTTCACTGTCATCTAACCCTGCCTTTTGTATT**CACTTCCACGGAAT**
**GAGACG**GACCGAAGTTAGCGAGGGGAGAGATTTATTTTATTGTTCATGAC
TTTAGTGACTTGAAGTACTGTCAGGACAGTTCTTCACCTGAACAGTGTTT

ACAGGTAGTAAAGTGGTTATTTCGGTAATTATTAGACATGAACCCAGCAA
AAATTATGCTGTGACATTTAAAAATGTTATTTATTTGTGCATCTGTCCAC
C**GGTTTGTCTCTCAGTGCAGC**TTTTACAACACTTTTTATTGTGGTCTTTT
CCTCTCTGTCCCTTTTTATAAAGCTCTTTGATCTAAACCTTTCAGAAAGT
GATGTTATTGAATACTGCGGAAATAAGTGCAGCCCATGCTGAAATGATGA
GTTACTCGCTGTACAGCAGCACCATGTTTTTAGCCTTTCAACTTCTATGT
AAAAGTAATTGAGGCCAGTGGAATAAAAGCATGCCTGGAAGCAGGACGGG
ACTTCAGCTGTAGCAGTAGGTTTGCCAATAATGGGCCAGTTTTCAAGTTC
AAGATTAAGTTTGATTTCAGTGGAATTTTTACACTGTATTAGTACTAAAA
TGTTTTATTTTACTTCAGTTTCAGTCTAATAATACTGATATCAGCCTTGG
GAAAAATCCATACAGCTGGGTAACTGAAATGCATCACTTGGTTTAGTAGA
ATCATGCATTCCTACACATCAGAACTCATTGCACTACAATAGATCTAATT
TTCCAAAGGAGGAAATATAAACCATGTAATGCGAAGATCTGGCAGACGGA
AGCTTTCGGCTGGTCACGTTGTGTTAACTGATATTTGACTGTGTGTTACA
GGTGGAGGTGTTGTATGGAGATGAGCCCTTGAAGGACTACTACACACTAA
TGGATATCGCCTACTTCTATGAGTGGAGACGGGTGAGTGACAGCTTTGAT
ACTAAATACTGTGGACCACAAATATTTAACAAACCTGAACTGGATCACTC
TTGTCTTTCTTGTAGTTAGTGTAATTTAATTTTTTTTGTTGTTTCAGTTA
AATGGGATGAGTAATCTGTCTTTAGGTTGGCTTGAAATGAGGAAAGTACC
AGTGCTTTAAATCATAGTGTCTGTTAAAGATTTGAACCAGAGGGAGATCA
GTCAGTCCTTCCAGGATTTCACTGGCTTTTTTGGGGAATGTTGCGGCCTG
AAATACCTGATTTCACAGCAGCTTTTCTAAAAGAAATTGTGATACATGTT
CAATGTTTTTAGGCATTTATTTTGCAATGAAATTGCTGGAGAAAGTGAAA
ATTGAAAAAAAAGAATTAGCTATTATTTTGTATAATTCATAAAAAAATTCA
AGGCAACTTGTTCCAGTGAGAATAAAACCATGAGAATAAAATTGGACAGG
GCAAATGTGAGACATTCATGGGACCCATGTCTGCATCTTTACGACCAGAA
ACAAGGAGGTGAGTTTGGCTCAATTGTTTTCTTGGGAAACTACAATGGTT
GGTAAAATTTGCAGAAAGGTTGCGGTGATTGGACAGAATTGGAAGGTTGT
GCAGAATTCACAGGGATTGGTTGATTGTTGCAATCACAACTTCCTGGAAG
GAATGCTCAGTAATGAAGCTACATGTTTAGAAGTTCGTTAACCTTCAGGG
TTTAGGGAGGTAGTCTGCTTTGTGCAGTTATGTTCAGGTGTTTGGATTCT
GATAAACACAGTCGCCTCAGGACACTACCATCACTCCAGGATCCAGTGGA
CTTTACAGAACACCTAGAACTGGCCAGTTGTTTGTCGGCTTTCTGCAGAA
ACAGAAGAAGCACTGTGGTCTCCCTGATTTTCCCCTCTTTGCAGTGTTTC
CAGGACTAACATACTACAAATCACTCGAGTTCACTGCTGACAATTCTCTT
GTGCATGCTTTTATGTTTTCAGATTGATTACATAATAAGGAGCCAATAAG
AGCTCATAATCTATCTATCTAGCATTCTATTCCTGTTAACATTTGGATGG
CTGCTACTCCAGCATTAATGTTTGGTGCATTCAAATGTTTGTAGGAAACT
GCAGCTCCAAGAGTCAGCAGCCAAATAGTAACTTTTTTTGTGCATGAAAT
GATCAAAGTCCCATGATCCCTAGTTTTTTGTCAATGTCAGATTGTAGTTT
GTCTTGTGAGGTTGATTTTCTCACAATGTAGCTTCTCAGTGTTTGTCTAG
ATTTCAAACTAGTCCCTGCTCTCATCTGTACAGCAACGTGACAATAACGC
AGCTGTCATTAAAAGATGAAAATAAAGCTGACTCATAGTTTCCCCACATA
AACTAAAACCAATGACTGACTGATGTTTTTGGAGGGAGCTCCTCTAAAAC
ACAATTTGTATCAACAGAAAGCAATAACTATAGTCCTTCATCATGCTGAC
ATTGTGGAAAACACGTTTACATAATGCCACAGTCATTTTAACTTATTGCT
GCTGTTTTGTTTATTTTTGTTTATTTTTGTTTTGGTTATATGTTTATAGT
TCATGTTGCTGCTGCGTTAGTGAAACATGCCAACAAAAATCAATAGTATC
ATCTTATCGAGTATGTTGCTCATTGAGAGTCCATATTCACGTGGTTGGCC
ATTTGCTATAAACTCCCTTTTCATATGGACGCACTCACAGCGGAGTTGGG
GTATCCTGGGTGGACAGAGTGAGCTGATAACCCTCTTTTTGAGATGGTTT
AACTGAGATTGATATTATAAAAAATGACATGAATTGAGTAGTTCCTACTA
ATATCAATATCCTTTTTGTCAATTGTCCTTAGGTAACAAACAACATTGTT
ATATTAAAGGATGGGGCTGGCGATCTGATCTGATGAAAAGACCAAAATAA
AATACCTCTCCATCCTACCCTTTCTTTGCCTCTCAGCCCAGGCTTATTCA
TTCCTAATAAAGATGTGATTCTTTAACAACTGGTAACAAAACAGCAGTGG
GAACTGTAGTTTTTGGCAAATATTACTTTAATAGTGCAGAGTATTGACAG
CAGCAGGACAGTGTATTTGGGACTAAGTCTAAATAAACGTGTGTGTGTGT
GTGTGTGTGTGATGATAATTAAGGAATATGTCACCCAGTGCAATAGTTTT
TGGACAACAATGGGACACAGATAATACTTAATAGGATCTATTTAGTGATG
GGTCTGGTCTTTTCACTAGATTTGTTGACAATTTTTCACCAGCCTTATCA

TTAAACTGTTAAAGACAGCAAGTTCTGTCAATAAATGTGCCCACATATTG
GTGAATTATAAAACCTGAGCTCTTATCCACAGTCCTAACAATGTGTGTAC
CTCTGTGCAGACTGGACCCATCCCATTGCAGTATCTGGTCAAACCCACCC
GGAAGCGCAGGAGGCCGTCCCAGTCTGCCGCCCAGGGCCACTCCGATGGC
GTCAACACCAGTCCGACGTCAGAGAGCGACTCCCAGAGTGACAAAGTCCA
CAGTCCCGCCACAGCCCAGCCGCCCCGAGCCTCCTCGCAGTCCAGCCCTG
CGTCCCATGACCTCTCTCCCGCCATCCAAAGCTCCAACGGTACCACTGTG
CCCAACAACACTCAGCGACACACTCTCGTCTCCAAACAGGGCGCCAACGC
TCGGAAGGTGACTGTCAACGGCACAAGCTCTGGGGCCGGCAAAGAGGAGG
CGAGGGGAGCCGACAAAAGCGGTTTGCCTCCGACAACCTAACGTGCATAC
GGGGGCAGTGGGGAGGACAGTACTGGAATTGCTCCCTTCTTTCTTTCTAC
AGAGTAAAAAGACGAGCTCAAGCCTTCTTCCTCCTGGACAAACCGGAGGA
AGATTACGGTCATCATCTTTCAGCAGGAAAGACTGAAAAGTGCAGCCAAA
TAGACACTTGTATGTATGTATGTGCGTGTGTGTGTTTGTGTGTTAGACTA
GAACAGTGCACACATACAAACTGTACAGTATGTATCAAATGTGAGCATGT
GTGCATACATATGTATAAACTTATCTTTTATACAAGATATTGTAATCGTT
TTGTATTGTATATGCAGTGGGTCTGTTTCATTTCCATAGTTCCATCAGCG
TTCATTTGGCTCCATTAAACTTGAGACAAAATGCTGAGAGGCATTGCAGT
GTACATCGCTGCAGGAGTTGGATGTAAAGAAGAAAAAAAAGCTAATCCTC
GCCTTTGAACAGTCATGTTAATAACTGCTGTATAAGTGTCCAGTATAGGC
AACCGCTCATCAGGTAACACAGGTACATTACATATAACTGTTGATGCACT
TTGTTGAGGTCAAATCACACAACCCAAACCAACAAGTATTTTCCGACAGG
AGAAGCATTTATATTTACAGAATACCAAGCACTACCATGCTTGCAAGCAT
TTGTTTGTCAGCAGACCACGTTCTCTCTCGCAGTCAAAGAACTCAAAAGG
GATTTGAATATTTGGCCATGTATGTTGTTGTTCCATATTTTCAACATTCC
TTTTGTTTCTTTTCAACAAACAATGATACTTTTTTATCAGCCATATGTGC
ATGTTTTAGGTCAATAAAATGTTTACTTACTAGGTTTTTCTGACCTGAGT
GTCCTGTTTATGATTCCATGAATGTAGCAGATTGTGAAGCATGCTTTTGT
GGATGTAGCACAAATGGAGAGGTAAAAAAACTAAATACATAACTCACTTG
GTAGAGCTGGTGCCCCATGTACCAAGGCTGAGTCCTTAAAGCAGCGGCCT
GTTGCCCTTTGCGGCAGCTCTCTCTCTCTCACTCATCAGGCAGTTACAGA
TTAAAAATGTAATCAGTTTAATAATTACAGGTACATAATGTAATGTCAAC
AGCTCTTTTTCAGTGCAGTTTCCTACAGTGAGCTACAGCACAAAGTGTTC
AGATCACTCATTGTGGCATAGTTCTCTGAACAGGCAAGGTTTAAATGTAT
TAATAATAGTGTGTGCGTGTACCCTAACCCAAAATGTGTCAGTAGCTCAC
GTGCAAAGCTGCTTGGAAGAAGATACAGTTAGCACTTTTCTCTGGGTCCT
ATCTGCAGGTCCGCCAGGGCCAACCCTGGTTGACATGATGAAGTTTCTGT
CAGAAGCATATAAATACATCCCCAAAAGTTAAAAAATAAATGTGGCATTT
TATATGTGGAGAGGAGGATTTATGGCCCTGACAGATATGCTACTTTACGG
AGTGAGCTGTAGGACACTGCACTATTACATCCTCCCTTCATACACACACT
TCAGTCTAAAACCTCTACACAGACAATATTCTCCTGTAAAAGCATTCTCT
AGATCTTGAGTAGTATTGAAGATAATACTGTTATATCCCATCTTTAACAT
CCTTTTCTACAGAGTGTGGTGGTAAAATCTCCACCCTCAGACAGTATATC
ATCTTATCATTCAACCCTGTTTCCCTGATAAACGACAGAAAGCAAGGGCT
TTTCATAGCCACTCTCTTCTCTGTCAAGCACTGAGAGAAATACTGATAAG
ACACAAGAATCAGACACTTGAAATGGTCAATCAGTTTACATGTGCTGTTC
AGTTCAAAGATCTCTCTGCATTCCTTTTCAAGAATCAGTTTGCTCATGTA
GCGGGGCAGATATGACGAAGTCCTGCTTGTGCGTGCCGTCACAGTAAGGC
GGATTGTGTGTGTACTTGCAACCACACAACCAAGCCGTGGCGTCTTTCTC
TGGGACAAAGCGTAGCGGGGACAGGCCTTGGGCTTTGGTTTTGTGGGCTC
CGTCGCAGAAAGGCTGAAAGATAAACATGTGATCAGGGAATATACAGGAC
GCGTACTTTAGACAGGAGTGATAGAGACTTAGACTTATTGATCCTTTT

**CACTTCCACGGAATGAGACG = forward primer (LG7:5,307,022 - 5,307,042)**
**GGTTTGTCTCTCAGTGCAGC = reverse primer (LG7:5,307,237 - 5,307,257)**
**195 bp**

*** *jarid2a*_European sea bass:**

>dicLab1_dna range=LG16:25684955-25690571 5'pad=0 3'pad=0 strand=+
repeatMasking=none

1887 TATAACCTGGGCTGGCTTGGTTCTTAAACGGCGGCTCACAGAACCGCAGT
1888 GGTGCTTGCCTTCCTGCACTGAACACCCCTGCAGGTGCCGACTTAAAGCG
1889 GCACACCGAGTCTAAAGGCTCAGTAAGGTGACTCCGGTACCGGTTGGATA
1890 TTTTACGTGACTCATTGCTTCAGGGTTTGGTAAAATGTGTGAGTCCGTGT
1891 GCCATTCCCTCAGGTTGTTGGTGTGCTCATGATGTAGGCCGGTGGTAATC
1892 CTGTTGGAGGTGGGTGATCGATTATTGTTTCATATTTTCTTTGTTCAAGG
1893 ATTAATATGACTATAATAGAGATGTCCGTTGTAATGTTTGTGTACAACAT
1894 GTGTATTCATGTAACGTTACAGCGTAGTCAGAATTGTAGACTGCGATCCA
1895 CATTTACCGGTACACCCTTCACTAATATCTGTTCAAATGTTGATGAGCGG
1896 CTCTGGCTGAGCTGGCTGTAGGCCGTTGTTCCAGATAAATCTGACTATAT
1897 TAATTTAACTGGTGGCGAATTCGTCGCTAGTTGGTTGTAGTAACGGCATA
1898 CAATTTAATGAGGAAGCTTCTTCCTGATACTTAATTAAGCACAAATGAGG
1899 ACTAATTCGAGCATAAATACGCGGCATTATGTAAATCCATTCAAATGTTG
1900 TGCTAGTGTATAGGCTATAACTGCATTTTTGTTAAATCTACATGTAACAC
1901 CTGATCTGGAGGGTTATTTGGTAATTGAATGACTTGGTCTGTAGGCCTTA
1902 AGGTTATTAACACAATCACTTTTTCCGCAGCTTGCATTATTAGTCAGTAT
1903 GTGCAGCCGAATTGAATCAGTGATTTTTTCTTTATATATATATATATATA
1904 TATATATATATATATATATATATATATGTTAACATAGATGATTATAGACA
1905 TGGGAAAACTAAGATGCAATCAATATCCTGCTGATATCCCACATCACCAG
1906 CTTCCATCTTGGTGCCCTTGAGCAAGGCTGGTTAAAGGGCAATTTCTAAT
1907 GTGAATTTCAATGAAAAGTACATGAATCAGAACTCTGCATTTGGAAAGTA
1908 TTTTTTTTTTTAACATAAGGTTATATTTCCTATTGTAAATCACATTTAGA
1909 TTTGATTATGGTTAGATATCATGATACTGACTGTACTGATGAAGAAAAAT
1910 CCCAAAACTCGCTGGCAGCTAGTTTACATTATCTACAGCTAGGTAATAGC
1911 TAGATGATGTATTTATTTTAACAAAAAAACCTCGTGTAACTTAAGGTCCA
1912 CTTACTAGCTGAACAAACTCCCAGTGGAGGGAGTTTGTTCAGTTGTCATA
1913 GAAATGGCCATGGTCATTATACTTACTTGGCTAGTAAGTGAACAAGGAGG
1914 CAAGAGTTTTTTATCAAACTGTTTTCAATTCTTGAGGCTTCCTACTTAGT
1915 TTGTGTGGACTTCTGCTTTGTTTTTACATCATGCTTTTTTCCACCATGTC
1916 TATCTGAAATTTCAAATATATTGTACAGAGCCAGTAAGGCGGTACTTGAA
1917 GGAAGTATTACCACTTTTAAAAAAAACCTAACACTATGGGACAGAATATT
1918 GATATTTTGTGTTGTGATCTTATTTTGAACACTGCTAATGTTTTCTTTCA
1919 GAAAAGAAAGCAAAACAGCCAAATTCAAAGGAACGAGGCTTCCATAATTC
1920 ATGTGCCCCTTAGTGTCTACTGGGCAAGATGAGTACAGACAGACCCAAAC
1921 GGAATATCATCAAGAAAAAATATGTAAGTGAAGTTTCTTTCTACTTTTAT
1922 TTTAAACATTATCCCTGGCTCTTCCTGTCACATGTCGAAGTATCCTTGAG
1923 CTGAACCCTAAATTGCTCCATCGGTGTATGAATGCGTTAGTTTCTGTTCG
1924 ATGGACTTTGCTATCACTGTATGAATGTGTGTGAAAGGGTGAATGTGGCA
1925 TACTGTATGAATGTGTGTGAAAGGGTGAATGTGGCATACTGTATGAATGT
1926 GTGTGAAAGGGTGAATGTGGCATGTAGTGTGCGATTTGAGTGGTCGGAAG
1927 ACTAGAAAGGCGCCATGCAAGTACAGGTCCATTTACCATTTATAGTGCCT
1928 TTCTAGTCTTCCGACCACTCAAAGCGCTTTATCACTTACATGCCACATTC
1929 TCCCACTCACACACCGATGCACAGCATCGTGAGCAATTTGGGGTTCAGTC
1930 TTTTGCTCAAGGATACTTGAGTTTCTGCGGATACCGATCCCGATCCCCGA
1931 TCCAATACTTTTCCTTAATAATACAGGTGAATAGCTCTGCTGGGCGGCTG
1932 TGGCTCAGAGGCAGAGCAGGTTGTCCACTAATTGGAAGGTTGTTGGTTCG
1933 ATCCCCGGCTCGTCCTGCCACATGTCGAAGTATTCTTGAGCAAGATACTG
1934 AACCCCAAATTGCTGTGAATGTGTATGAATGTGTTTCTGTTCGATAGGGT
1935 TTGCCATCGCTGAGTCAGTGTGTGTGAAAGAGTGTATATGGCATGTAGTG
1936 TAAAAGCGCTTTGGGTGGAAGACGCGCTATACAAGCACAGGACCATTTAC
1937 CATTTTACCATTAAGAAAAAAAATTGCCTGCAACCAAGCTTGCCTCTGAC
1938 CAGG**GGCTGAGCTCATGCATACAC**ACAGCACAGTGACATAGACAGAGAGA
1939 GTTGACGGCGAAGCACTGTGCATGAAAAGATATTTTGTACATTTTACTGT
1940 AGTATGATCATGTTTGATGAAATTTATATAATTTGAGCAGAAATAATCCA
1941 CAGCTGGCAGTGTGAACAAAGCTGATGGTGTTTGTGTCTGACTCTGCAGG
1942 ACATCAGTGATGGGATGCCA**TGGTGTGAAGAACGTCTGGT**CCGGAAAGTT
1943 CTTTTCCTGTCCCTCAGGGAATTCAGAGACACACACCGCACCACACACAA
1944 TCACTCACACATTCACACACGCACACACACACACAAACGCGTGTCAAAAA
1945 ATACACTTTTACTGCAAAAGACGCAGACACTACCTAACACGCACAGACAA
1946 AAAAGCATACAGACAAGACAGAGTGTGCACACGTCACAACAAAAAGTCAC
1947 ACACAGACCACAGAATGTACACACACGAAAAAACACACACAGTGCACAAA

CTATGCACATGTCCAAACACCTACGCCCTCATGAACACAACAACAAAGGA
CAAAAAACAAACTTGTCTCAGGACTCGCACACACCAAAAAATAAATGTAC
TCACACACTACAGAACATGCAAGTAAAATCAAAAATGTGTACAGGGAAAA
TTACACACACTGAACAGCACACACACCTACAAGAAAATACACACATGTTC
CTGAAGAAGTCTGTATCAACCAGGACACGCTCACAGAAATCACAAAATAG
ACTGTCGCTGCTGAACTCGACATATATAGAAACTGCAAAACACATGCATA
GTACTCAAAACAAACACTCAGTGAGAAACACACAGACACTGCTGAGCACT
CCTGTCCCGTCCAGGACACTGAGGTCACACACTCCCACAAGCCTCAGTGG
TCAGTACCATCAATACTCCTCCTCTGTTAGTAATTAAGTAGTACTGCCGC
TACTGTAGAAGTTCCTTCTAAAGATAATTCCAGATAGTTACCTGCTCTTA
TTTTAATTTTTTTTATAAATCAGTAATCACAGCCTTGTACTCACTAAGTT
TGAGATATGGGAACATCGTGGCATAACTAAAAAGAGTCTGACGTGACATA
AAACATGACGTGTCAGATAGGTTGGAAACTAGGCAACGGTTTATCCAAAC
TATCCAAACCACTTTACTTGGAGGTCAAACAGAAAGTGAACATACTTGTA
ATTTCACTAAGAATTCCTCATTGCACAGGAAAACTGTGCATACAACTACA
TTAAGTAAATTCTGTCAGTTAAGTCAGGAAGTGAGTCTGTAAGCTGTGAT
TGAGGTTTACAATGTACAGTTGTTTTTTTTTACATTTAATTTTAACCATA
TTTTTTTCTTAAAATAAGTCTGTTTTCCAGCTTGTCTAATTGACTGCTTG
TGTTCAACAAACATACACAGCTGCAACTTAGATGTATTTGGGGCTGAAGC
TTATGTACTTCTTTGGTTTATTTGAAGTGTCCATGAAATGAGGCTTTGAT
ATTCGCTTAGCTGTTAACACCAGCTCTTTCCTGTCATTATTTTTAGTATG
TCATTTGCTGTTTTTGTTATAGTTTTATCCATGCAGATATACATTGACAT
TGTATGTGTAGATCATTTTTCAAAGTACTAAAATTGTTTCATGTAAGTAA
CTCACATTTCAACTGTTCTGTGGCTCCAGGCTCCCTGGTTAATGGCATCA
ACCGGTGTCAGTCCCTCCTGTCAGCTAGTCCAAGCTGGAGCTGGCACTCA
CGAACACACCCTCAACAGTGCTGCCCTGCTAGCATCCATCATGACAAGGA
TGACCCTGCCACCAAGAGGTCTGCTGCTGTAGTGTGTTTTTGCATGTTCA
TTCCTATGTGCTGATCAGATGACAAATGCAAATCAAGCAGTAGTCATGAT
AACTACTTGTGTCGTCCAACACAAGTGTGTGTGTGTGTGTGTGTATATAT
ATATATATATATCTATATATACATGCGCCCACACACACACACACACACAC
ACACACAAACACACACACACATACATATACATATATATAATTCCCACCAT
GAGCAAGCACTTGGCATCAGGGGCAGGGAAAAACTGTGATTGCATTTGTG
ATATTCTCTAAAAGAAAAAATTTGACCCTGCCAGTTGTAGTTTTAATAGT
CCTGCTATTAGAAAGTCTGTGGTGCTGTGCAATTGTACTGAATTAGAGAC
ACAGATGTGTTTCTCTTATTTAGCCATTGATATAAATGGGATGGACAAAA
TAATAGTTTAGTTTAGTTTAATGCAATAAACACTTTATACAGTTGAATCA
AAAACTCCATGAGGTTAGGATGTATTACAGGACTGATTCTACTAGGACGT
ATTAGATTACATTCGTTTCAGCTAGGTGTTCCTAATAAACTGATGCACAT
GCATGTGCAGACCAAGGCTGCAAGCACAGAGGAAGTTTGCCCAGTCGCCT
CCAAGCTCTCCAGGACCACCAGTGTTGATGACTTCAGCACGAAGTAACCA
CACCCACAACCTGGCTGTAGTCTCCTGTCTGACTAGACGTCGACCCAAGA
CTGAGGACTTCTTGTCTTTTCTCTGTTTGAGAGGTAAGCTGATTTACTAA
ATGGATTTAACCTCTACTCAATAATTTTATAACAAAAGTGACAAATGTAA
GCATATTAGATAAAAAAGGAGTCCTGTTTTGTTTTTTGAGGCAAGGGCGC
CGCCATCTTGTAGCACTCTGACCTACAACATCAGAGGGTTGGTTGTATAT
GTTCCACAGACCCAACATAGAGTGAAAGACAGACACACAGAAAGACAGAC
ACTGAATGACAGAGACACGGAAAGACAGACACTGAATGACAGAGACACGG
AAAGACAAAGACACTTAAAGATGGAGACTGAAGAGCCACAGCACAGAAGA
AGACAAAAGGGAGGCCACTATTGTATTGCTCCAGGCTGTAAAAATGAGTT
TTATAGGGTGAAAGCCAAGGACAAAACAGTAAATTTTCATAAAGTGCCGC
TGAAACAGAGAACGGTGCTGCTGCGCTGGCTAGCGGCACTGAAGAGAACG
AGCCTACCCATGAGCGA

**GGCTGAGCTCATGCATACAC = forward primer (LG16:25,687,509 - 25,687,529)**
**TGGTGTGAAGAACGTCTGGT = reverse primer (LG16:25,687,707 - 25,687,727)**
**198 bp**
