# Supplementary material for: Effects of Sodium Butyrate Treatment on Histone Modifications and the Expression of Genes Related to Epigenetic Regulatory Mechanisms and Immune Response in European Sea Bass (Dicentrarchus Labrax) Fed a Plant-Based Diet
Source: PLoS One. 2016 Jul 29;11(7):e0160332. doi: 10.1371/journal.pone.0160332 (PMC4966935; doi:10.1371/journal.pone.0160332)
Supplement: S1 Table — (PDF) [file pone.0160332.s003.pdf]

S1 Table. Quantitative real time PCR primer characteristics

Gene	Forward	Reverse	Product size (bp)	Efficiency	Strand	Amplified region (genome position)
il1β	GCTCGGACAGATGCAAAGGT	CAAATGTAGGCCTACACCTA	165	2.3	-	LG20:18,235,583 - LG20:18,235,763
il10	AGAGGCTGCATGGTTTCTGT	CATCACTCTTGAGCTGGTCG	198	1.8	-	LG1a: 13,840,422 – LG1a:13,840,660
irf1	GTCATGCTCACACTGACCCA	AGTTTCTTCTGGCGCTGACT	157	2.4	-	LG14: 21,270,348 - LG14: 21,270,447
muc2	TCCATTTCCACCTACAACACAG	TACGCCTGCGTTGTAGTATG	183	2.0	+	LG6:12,932,125 - LG6: 12,932,352
tnfα	CTCAACACAGCGGATATGGA	GCTGAGAAAACAGGTGATTA	257	2.0	+	LG16:3,707,397 - LG16:3,707,694
r18S	CCGCTTTGGTGACTCTAGATAACC	CCTATCAACTTTCGATGGTAC TTTCTGTG	50	2.0	+	UN:96,922,353 - UN:96,922,469
dicer1	GCAGTACCGGAGCAGACTTA	AATGATGCTTTCTTTCTGTCC	226	2.0	-	LG12:1,244,797 - LG12:1,245,064
ehmt2	TGTGTTTGATGCATGGTGCT	TGCCCTGACACATGAAGTCT	172	2.2	+	LG9:18,403,519 - LG9:18,403,731
hdac11	ACAGCACTACTGGAAGCACT	AACGGGTGAGAAGAACGTCT	197	2.2	-	LG13:22,907,294 - LG12:22,907,531
jarid2a	GGCTGAGCTCATGCATACAC	TGGTGTGAAGAACGTCTGGT	198	2.0	+	LG16:25,687,509 - LG16:25,687,727
pcgf2	CACTTCCACGGAATGAGACG	GGTTTGTCTCTCAGTGCAGC	195	2.1	-	LG7:5,307,022 - LG7:5,307,257

* The efficiency [E=10^ (-1/slope)] is the value used to correct for differences in primer efficiencies when comparing qRT-PCR expression among the groups. Efficiency has been transformed to a percentage by applying the formula [% E = (E-1)*100]. Efficiency transformation results were as follows: *il1β* (130%), *il10* (80%), *irf1* (140%), *muc2* (100%), *tnfα* (100%), *r18S* (100%), *dicer1* (100%), *ehmt2* (120%), *hdac11* (120%), *jarid2a* (100%), and *pcgf2* (110%).
