# Supplementary material for: Effects of Sodium Butyrate Treatment on Histone Modifications and the Expression of Genes Related to Epigenetic Regulatory Mechanisms and Immune Response in European Sea Bass (Dicentrarchus Labrax) Fed a Plant-Based Diet
Source: PLoS One. 2016 Jul 29;11(7):e0160332. doi: 10.1371/journal.pone.0160332 (PMC4966935; doi:10.1371/journal.pone.0160332)
Supplement: S2 Table — (PDF) [file pone.0160332.s004.pdf]

**S2 Table.** Statistical analysis by two-way ANOVA of fish growth data.

| Effect | df Effect | MS Effect | df Error | MS Error | F | p-level |
|---|---|---|---|---|---|---|
| DIET | 1 | 3.654 | 169 | 21.28411 | 0.1717 | 0.679158 |
| TIME | 2 | 3107.577 | 169 | 21.28411 | 146.0045 | **0.000000** |
| DIET*TIME | 2 | 18.360 | 169 | 21.28411 | 0.8626 | 0.423913 |
