# Supplementary material for: Effects of Sodium Butyrate Treatment on Histone Modifications and the Expression of Genes Related to Epigenetic Regulatory Mechanisms and Immune Response in European Sea Bass (Dicentrarchus Labrax) Fed a Plant-Based Diet
Source: PLoS One. 2016 Jul 29;11(7):e0160332. doi: 10.1371/journal.pone.0160332 (PMC4966935; doi:10.1371/journal.pone.0160332)
Supplement: S3 Table — (PDF) [file pone.0160332.s005.pdf]

S3 Table. Quantitative real time PCR: fold changes (FC) in the expression of genes related to epigenetic regulatory mechanisms and statistical analysis.

Gene	Intestine FC ± SEM$^+$	Intestine Student t-test		Liver FC ± SEM$^+$	Liver Student t-test	
dicer 1	2.40 ± 1.864	t	0.548	5.88 ± 3.393	t	1.068
		P-value	0.596		*P*-value	0.313
ehmt2	1.18 ± 0.869	t	0.498	2.52 ± 1.617	t	1.421
		P-value	0.629		*P*-value	0.169
pcgf2	1.29± 0.932	t	0.114	14.75 ± 9.485	t	-0.15
		P-value	0.911		*P*-value	0.886
jarid2a	2.66 ± 2.186	t	0.893	11.06 ± 4.028	t	0.898
		P-value	0.395		*P*-value	0.378
hdac11	0.49 ± 0.200	t	-1.388	1.67 ± 0.774	t	1.791
		P-value	0.19		*P*-value	0.099

Note: Asterisks mark statistical differences with $p<0.01$.
