# Supplementary material for: Effects of Sodium Butyrate Treatment on Histone Modifications and the Expression of Genes Related to Epigenetic Regulatory Mechanisms and Immune Response in European Sea Bass (Dicentrarchus Labrax) Fed a Plant-Based Diet
Source: PLoS One. 2016 Jul 29;11(7):e0160332. doi: 10.1371/journal.pone.0160332 (PMC4966935; doi:10.1371/journal.pone.0160332)
Supplement: S4 Table — (PDF) [file pone.0160332.s006.pdf]

S4 Table. Quantitative real time PCR: fold changes (FC) in the expression of genes related to inflammatory response, mucosal protection, and immune homeostasis plus statistical analysis.

Gene	Intestine FC ± SEM$^+$	Intestine Student t-test		Liver FC ± SEM$^+$	Liver Student t-test	
il1β	1.83 ± 0.693	t	1.208	0.07 ± 0.035	t	-2.155
		P-value	0.255		*P*-value	0.083
il6	0.47 ± 0.113	t	-0.109	0.01 ± 0.006	t	-1.071
		P-value	0.915		*P*-value	0.309
il8	1.77 ± 0.907	t	0.874	0.69 ± 0.342	t	-0.603
		P-value	0.411		*P*-value	0.560
il10	1.13 ± 0.242	t	1.792	25.09 ± 17.176	t	3.361
		P-value	0.098		*P*-value	0.002**
irf1	0.17 ± 0.034	t	1.398	4.74 ± 2.777	t	1.329
		P-value	0.19		*P*-value	0.211
tnfα	1.54 ± 0.303	t	1.761	0.34 ± 0.323	t	-1.144
		P-value	0.109		*P*-value	0.282
muc2	0.74 ± 0.226	t	-0.553	0.22 ± 0.146	t	-1.820
		P-value	0.591		*P*-value	0.291

Note: Asterisks mark statistical differences with $p<0.01$.
